# Identification and bioinformatic functional analysis of novel and known polymorphisms in the myostatin gene of Ukrainian Carpathian Mountain sheep

**DOI:** 10.1038/s41598-026-44326-6

**Published:** 2026-03-23

**Authors:** Tetyana Buslyk, Mykyta Peka, Artem Saienko, Mariia Kozak, Marian Yuzviak, Yuriy Salyha, Petro Stapay, Ilva Trapina, Natalia Paramonova

**Affiliations:** 1 Institute of Animal Biology, National Academy of Agrarian Sciences of Ukraine, 38 Vasyl Stus St, Lviv, 79034 Ukraine; 2 Institute of Pig Breeding and Agroindustrial Production, National Academy of Agrarian Sciences of Ukraine, 1 Shvedska Mohyla St, Poltava, 36009 Ukraine; 3 Department of Molecular Biology and Biotechnology, School of Biology, V. N. Karazin Kharkiv National University, 4 Svobody Sq, Kharkiv, 61022 Ukraine; 4 Laboratory of Genomics and Bioinformatics, Department of Pharmaceutical Sciences, Faculty of Medicine and Life Sciences, University of Latvia, 1 Jelgavas St, Riga, LV-1004 Latvia

**Keywords:** Myostatin, Ukrainian Carpathian Mountain sheep, Single-nucleotide polymorphism, Mutation, Sequencing, In silico analysis, Bioinformatic prediction, Molecular dynamics, Causality, Rational selective breeding design, Computational biology and bioinformatics, Genetics, Molecular biology

## Abstract

Myostatin (MSTN) is a well-established negative regulator of muscle growth and development in mammals. Genetic variations within *MSTN* are linked to differences in sheep musculature, particularly the double-muscle phenotype. This study represents the first comprehensive polymorphism analysis within intron 1 of the *MSTN* in the Ukrainian Carpathian Mountain (UCM) sheep breed combining molecular and bioinformatic approaches. Sequencing data of samples from UCM sheep revealed eight previously reported single-nucleotide polymorphisms (c.373+241T>C, c.373+243G>A, c.373+246T>C, c.373+249T>C, c.373+259G>T, c.373+323C>T, c.373+563G>A, c.373+607G>A) and one novel polymorphism (c.373+283T>C). Bioinformatic analysis evaluated potential functional effects of these intronic polymorphisms, including changes in pre-mRNA stability, proximity to transcription factor binding sites, and possible pre-miRNA formation, using in silico approaches, including molecular dynamics simulations of predicted pre-miRNA structures. Predictions identified c.373+607G>A and the novel c.373+283T>C polymorphisms as potential candidates for further functional investigation and association studies. This study demonstrates the value of combining molecular genetic and bioinformatic approaches for characterizing intronic polymorphisms and supporting a deeper understanding of functional genetic variation in livestock.

**Supplementary Information:**

The online version contains supplementary material available at 10.1038/s41598-026-44326-6.

## Introduction

The Ukrainian Carpathian Mountain (UCM) sheep breed is a distinctive asset of Ukrainian livestock farming. Its uniqueness is determined by a combination of biological characteristics and economic significance, particularly in the context of mountain region development. Sheep farming is an integral part of the culture, traditions, and way of life of the Hutsuls and other ethnic groups of the Carpathian region. The breed was developed by crossing local coarse-wool ewes of the “tsakel” type with rams of the Tsygai breed and was officially recognized as a new livestock breed on December 31, 1993^1^. UCM sheep breed is well adapted to mountain environments and harsh climatic factors, including cold, temperature fluctuations, strong winds, abundant precipitation, and high humidity^1,2^.

Although historically the breed was oriented toward wool and milk production, it is considered a “triple-purpose” animal, producing wool, milk, and meat. However, modern market demands have shifted the breeding emphasis from wool to meat production, while maintaining the high adaptive capacity required for mountainous environments^3^.

Marker-assisted selection based on molecular genetic markers is widely applied in global sheep breeding to accelerate improvements in meat productivity, particularly in small populations of local breeds. This approach holds significant potential for increasing the rate of genetic progress in traits related to growth and carcass quality across various sheep breeds^4,5^.

Many genes are involved in the regulation of animal growth parameters and determine final carcass weight, meat yield, and feed efficiency. The myostatin (*MSTN*) gene is considered as one of the most promising candidates for marker-assisted selection due to its significant impact on muscle development and growth^6–10^. The *MSTN* gene encodes the protein myostatin, also known as growth and differentiation factor 8, and is highly conserved among mammals and other vertebrate species^11^. This high degree of conservation suggests that the *MSTN* gene and its product have played an important role in muscle development and function throughout evolutionary history^12^. Located within evolutionarily conserved genomic region, MSTN acts as a negative regulator of muscle growth through a complex signaling pathway^11,13–15^.

Studies have identified polymorphisms in *MSTN* that affect muscle growth, carcass characteristics, and exercise endurance in various mammalian species, including humans, mice, cattle, sheep, goats, and horses^11,16,17^. In addition to its effects on muscle development, *MSTN* variation has been associated with lipid metabolism and energy-related traits, including the fatty acid composition of milk in cattle^18^. Moreover, recent studies have demonstrated that *MSTN* inactivation can alter the composition and functional potential of gut microbial communities in sheep^19^, cattle^20–22^, and pigs^23^, highlighting a broader role of *MSTN* in systemic metabolic regulation and growth performance.

The pleiotropic function of *MSTN* are linked to multiple physiological regulatory processes in mammals. In particular, *MSTN* negatively regulates muscle precursor cell proliferation by inhibiting cell cycle progression and differentiation and has also been implicated in the regulation of programmed cell death (apoptosis), glucose metabolism, adipocyte proliferation, regulation and homeostasis of skeletal muscle and cardiomyocytes, as well as bone and skeletal muscle formation and renewal^12,24–29^.

One of the key features of the *MSTN* gene is the presence of “causal” polymorphisms in sheep and cattle associated with the double-muscle phenotype, which is caused by MSTN deficiency and consequently leads to increased skeletal muscle growth^11,30–34^. Known “causal” polymorphisms are presented by frameshift indels or missense mutations, which alter the structure of MSTN protein or lead to the generation of truncated protein products. However, most polymorphisms identified in sheep are non-coding, and their molecular mechanisms of impact on phenotype remain poorly understood^11^. One notable example is a single-nucleotide polymorphism (SNP) in the 3′-UTR (g.6723G>A), which has been associated with miRNA binding^35^. Given the abundance of non-coding polymorphisms in sheep, it remains essential to clarify their effects on *MSTN* expression and muscle growth regulation. In this context, the application of in silico predictive methods appears particularly promising.

Most studies in sheep of different breeds have focused on the polymorphisms in the 3′-UTR region of the *MSTN* gene (c.*1232G>A)^36–43^. According to our previous data, the 3′-UTR region of the *MSTN*, which is polymorphic in many other sheep breeds, was completely monomorphic in UCM^44^. At the same time, intron 1 of the *MSTN* gene has attracted increasing attention in livestock research because of its potential regulatory role. Multiple polymorphisms within intron 1 of the *MSTN* gene have recently been investigated in cattle^45^, goats^46^, sheep^37^, and other animals, yielding promising results regarding their potential impact on animal productivity traits.

Variation in non-coding intronic regions can significantly affect animal phenotypes by modulating transcription, splicing, and recombination, thereby influencing the expression of genes that determine economically important traits. Sequence alterations may interfere with regulatory protein binding or reduce the efficiency of lariat formation during splicing, while changes in pre-mRNA secondary structure can alter splice site accessibility and recognition by the spliceosome^47–49^. Sjakste et al.^50^ suggested that such intronic variation may promote the formation of novel RNA structures, such as additional hairpin loops. Moreover, intronic regions can give rise to miRNAs, which regulate gene expression at the post-transcriptional level and play key roles in physiological processes, including skeletal muscle development. Several muscle-specific miRNAs involved in myogenesis have already been identified in mammals^24,51,52^. Thus, analysis of intronic polymorphisms in the sheep *MSTN* gene is of interest for testing the hypothesis that such variants may exert regulatory effects by altering primary transcript structure, transcription factor binding sites, or the formation of miRNA precursors.

Based on these considerations, intron 1 of the *MSTN* gene was selected as a target region for the search for potentially useful genetic variations. The aim of this study was to identify alleles and genotypes of the *MSTN* gene in UCM sheep breed using the observed genetic variations (polymorphisms) and to predict the molecular-level effects of the observed polymorphisms using bioinformatic approaches. Thus, in contrast to previous studies of *MSTN* intron 1 in sheep, which primarily reported polymorphism frequencies and/or trait associations, the present work additionally applies a functional in silico prioritization workflow, including molecular dynamics simulations, alongside genotyping of *MSTN* intron 1.

## Methods

### Sampling and DNA extraction

This study was designed as a population genetics analysis without experimental treatments or control groups. The experimental unit was defined as an individual animal. A total of 54 purebred UCM sheep of both sexes (23 rams and 31 ewes), bred on the “Gafynets” farm, registered as a breeding reproducer in the State Register of Breeding Entities in Animal Husbandry (registration number UA4361829031/AA №010881), in Pavshino village, Mukachevo district, Transcarpathian region, Ukraine, were selected for the study. The sample size was determined by the availability of purebred UCM sheep on the farm. Inclusion criteria required animals to belong to a single generation of the purebred UCM population, with controlled representation of both sexes. Relatedness of animals and the presence of twin pairs were monitored. Animals were selected without prior knowledge of their genotypes. None of the sampled animals had undergone previous experimental procedures.

Approximately 4 mL of blood was collected from each animal using vacutainer tubes containing EDTA as an anticoagulant. Sample collection, animal management, and care followed the recommendations of EU Directive 2010/63/EU. For genomic DNA extraction, a DNA extraction kit (Zymo Research, USA) was used according to the manufacturer’s instructions for extraction from whole blood, serum, and plasma samples. DNA quality and quantity were initially assessed using agarose gel electrophoresis and spectrophotometry. Subsequently, a NanoDrop ND-1000 spectrophotometer (Thermo Scientific, Wilmington, USA) was used to verify DNA quantity and quality. Samples were then diluted to a final concentration of 50 ng/µL with ultrapure water and stored at 4 °C until use. Laboratory staff conducting DNA extraction were aware of sample identity to minimise potential bias, but sequencing and data analysis were performed without this awareness.

### Sanger Sequencing and analysis of detected SNPs

UCM sheep breed genomic DNA fragment 1,062 bp long, containing a partial sequence of the intron 1 of the sheep *MSTN* gene (total intron 1 length: 1,833 bp), was amplified by polymerase chain reaction (PCR). The primers used for the PCR were: forward 5′-TATGCTAATGAGACTGAAAG-3′ and reverse 5′-ACACTGTCTTTAGGGTCAG-3′^53^. PCR was performed in a 50 µL reaction mixture containing 100 ng of sheep genomic DNA, 1× Taq reaction buffer, 5 nmol dNTPs, 20 pmol of each primer, and 0.25 units of Taq DNA polymerase (New England Biolabs). The thermal cycling program included an initial denaturation at 94 °C for 5 min, followed by 35 cycles of denaturation at 94 °C for 30 s, annealing at 54 °C for 30 s, and extension at 68 °C for 1 min, with a final extension at 72 °C for 10 min.

PCR products were sequenced by the Sanger method at Explogen LLC (Lviv, Ukraine). Сhromatograms were analyzed using FinchTV software (Geospiza Inc.) to identify possible mutations in the intron 1 of *MSTN*. Sequence information for the sheep *MSTN* gene is available in the Ensembl 115 database (ENSOARG00020077637)^54^. It was used for genomic region reconstruction. Multiple sequence alignments were generated using Clustal Omega^55^. The illustration of the structure of the *MSTN* gene fragment with the localization of the identified polymorphisms was made using IBS 2.0 software^56^.

### Bioinformatic prediction of SNPs’ effects

#### Prediction of SNP effects on pre-mRNA folding

To predict the molecular effects of the identified SNPs in intron 1 of the sheep *MSTN* gene, a set of bioinformatic methods was applied. They included the assessment of impacts on pre-mRNA secondary structure, identification of potential transcription factor binding sites, and evaluation of the possibility of miRNA formation from the intronic sequence.

Given the large size of the *MSTN* pre-mRNA, structural analysis was carried out on the region corresponding to intron 1. Changes in minimum free energy (MFE) and relative entropy resulting from the mutations were first evaluated using the remuRNA (version 1.0)^57^ via the MutaRNA web server (version 1.3.0)^58,59^. Subsequently, structural differences between wild-type and mutant pre-mRNA sequences were assessed using RNAsnp web server (version 1.2)^60,61^ that computed Euclidean distances with associated *p*-values. The analysis was carried out in mode 1 with default settings, including 200-bp flanking regions on either side of the SNP (folding region), 50-bp minimum length of the sequence interval (local region), and a base-pair probability cut-off of 0.01. In addition, RNAsnp was used to predict optimal secondary structures of pre-mRNA regions containing SNPs and to assess mutation-induced changes in MFE^60,61^.

#### Prediction of SNP effects on transcription factor binding

RegNetwork 2025^62,63^ and TRRUST (version 2)^64^ were used to identify transcription factors potentially involved in the regulation of *MSTN* expression. Then motif profiles for these transcription factors were retrieved from the JASPAR 2024 database^65^ in the MEME format of letter probability matrices. The search for transcription factor binding motifs in the intron 1 of sheep *MSTN* gene, including SNP-containing regions, was performed using the FIMO tool^66^ within the MEME Suite (version 5.5.8)^67^, applying a significance threshold of *p* < 0.001.

To assess sequence conservation across species within the *MSTN* intron 1 region containing the studied polymorphisms, a cross-species multiple sequence alignment was obtained using Ensembl.

#### Prediction of miRNA formation and dynamics

The potential formation of miRNA hairpin structures within intron 1 was evaluated via miRNAFold^68,69^ under default parameters (sliding window size of 150; percentage of verified features, 70%). Predicted pre-miRNA candidates were subsequently classified as true or pseudo using miRBoost^70^ with cross-species dataset and classifier weakness parameter set to 0.25. For true pre-miRNAs that included SNP regions, the structures corresponding to different allelic variants were compared using MFE. The predicted hairpin sequences and their genomic coordinates were additionally checked against annotations in the miRBase^71,72^ and miRNASNP-v4^73^ databases for sheep and other ruminants (goats, cattle) to assess potential overlap with known miRNA loci.

Based on the predicted secondary structures, tertiary structure modeling of pre-miRNAs carrying different allelic variants was performed using RNAComposer^74,75^. Non-canonical base pairs were excluded from the input of secondary structures during modeling. The resulting three-dimensional models of the pre-miRNAs were subsequently used for molecular dynamics simulations in GROMACS 2023.3^76,77^. For each modeled RNA molecule, two independent simulation repeats were performed with independently generated random atomic velocities. For each RNA molecule, topologies were generated using the CHARMM36m force field^78^. The molecules were solvated in a dodecahedral simulation box with a minimum distance of 2.0 nm from the RNA to the box edge, filled with TIP3P water and neutralized with NaCl to a final concentration of 150 mM. The energy minimization was performed prior to equilibration.

Equilibration was carried out in three stages: (1) 1 ns of NVT ensemble using the V-rescale thermostat at 310 K with a temperature coupling constant of 0.1 ps; (2) 1 ns of NPT ensemble using the V-rescale thermostat at 310 K with a temperature coupling constant of 0.1 ps and the C-rescale barostat at 1 bar with a pressure coupling constant of 2.0 ps; (3) 10 ns of NPT with V-rescale thermostat (310 K, temperature coupling constant 1.0 ps) and C-rescale barostat (1 bar, pressure coupling constant 5.0 ps). A 150 ns production run followed, using the temperature and pressure parameters from the final equilibration step.

A time step of 1.6 fs was employed throughout the equilibration and production stages to ensure numerical stability, as some RNA bond vibrations exhibited oscillation periods shorter than those compatible with the standard 2 fs step. The Verlet cutoff scheme was applied for neighbor searching, with short-range cutoffs of 1.2 nm for both van der Waals and electrostatic interactions. The van der Waals forces were smoothly shifted to zero between 1.0 and 1.2 nm using the force-switch modifier. Long-range electrostatics were treated using the Particle Mesh Ewald (PME) method with cubic interpolation and a Fourier grid spacing of 0.16 nm. The minimum distance between periodic images was maintained to be no less than the nonbonded interaction cutoff parameters.

Trajectory analysis included the calculation of the root mean square deviation (RMSD) for sugar-phosphate backbone atoms, root mean square fluctuation (RMSF) for the C1′ atoms of the ribose, the radius of gyration (Rg), and the solvent-accessible surface area (SASA) of the entire RNA molecules^79,80^. Hydrogen bonds (distance ≤ 0.35 nm, angle ≤ 30°) were analyzed throughout the simulation trajectories for the entire RNA molecule, as well as specifically for atoms involved in base-pairing interactions, including canonical, wobble, and Hoogsteen geometries.

To identify dominant conformational states and dynamic behaviors, trajectories of pre-miRNAs of the same sequence length but different allelic variants were fitted based on the sugar-phosphate backbone of a representative pre-miRNA structure carrying the reference allele and subsequently concatenated. Principal component analysis (PCA)^81,82^ was performed using the C1′ atoms of the ribose, PCA projection on the first two principal components were obtained, followed by construction of the free energy landscape (FEL). Visualization of the structures of pre-miRNAs at different time points of molecular dynamics simulations was done using open-source PyMOL software (version 2.5).

### Statistical analysis

Statistical analysis was performed using GenAlEx 6.5 software^83,84^. The observed heterozygosity (*H*_*o*_) was calculated according to the formula:

$${H}_{o}=\frac{{n}_{het}}{n},$$

where *n*_*het*_ is the number of heterozygous individuals at a given locus, and *n* is the total number of individuals in the sample.

The expected heterozygosity (*H*_*e*_) under Hardy-Weinberg equilibrium (HWE) was calculated as:

$${H}_{e}=1-\sum{p}_{i}^{2},$$

where *p*_*i*_ is the frequency of the *i-*th allele at the locus.

The Wright fixation index (*F*_*IS*_), representing the inbreeding coefficient for the studied population was calculated using the formula:

$${F}_{IS}=\frac{{H}_{e}-{H}_{o}}{{H}_{e}}.$$

The polymorphic information content (*PIC*) was calculated according to the formula:

$$PIC=1-\stackrel{n}{\sum_{i=1}}{p}_{i}^{2}-\stackrel{n-1}{\sum_{i=1}}\stackrel{n}{\sum_{j=i+1}}2{p}_{i}^{2}{p}_{j}^{2},$$

where *p*_*i*_, and *p*_*j*_ are the frequencies of the *i*-th and *j*-th alleles at the locus.

For each locus, a χ^2^ test was performed using the formula:

$${\chi}^{2}=\stackrel{k}{\sum_{i=1}}\frac{(O-E{)}^{2}}{E},$$

where the summation from *i* to *k* genotypes is based on the observed number of individuals of the *i*-th genotype (*O*_*i*_), and the expected number for the *i*-th genotype (*E*_*i*_) under HWE.

For biallelic loci in this study the corresponding number of degrees of freedom was equal to 1 (*df = 1*). Loci with χ^2^ values greater than the critical threshold at *p* < 0.05 were considered to deviate significantly from HWE.

Haplotype phases were reconstructed from unphased diploid sequence data using DnaSP v6^85^ with the PHASE^86,87^ algorithm for haplotype inference. Subsequently, the frequencies of distinct unphased sequence motifs (containing ambiguity-coded nucleotides) and inferred haplotypes were calculated.

The linkage disequilibrium (LD) between pairs of SNPs was evaluated using DnaSP v6^85^, based on the standardized disequilibrium coefficient *D*′, the squared correlation coefficient *r*^*2*^, and the Fisher’s exact test of non-random association between alleles.

For two biallelic loci each with alleles A/a at the first locus and B/b at the second locus, the standardized linkage disequilibrium coefficient D′ was calculated as:

$${D}^{{\prime}}=\frac{D}{{D}_{max}},$$

where

$$D={p}_{AB}-{p}_{A}{p}_{B},$$

${p}_{AB}$ is the frequency of the haplotype carrying alleles A and B, ${p}_{A}$ is the frequency of allele A at the first locus, and ${p}_{B}$ is the frequency of allele B at the second locus,

$${D}_{max}=\{\begin{array}{c}min\left({p}_{A}\right(1-{p}_{B}),(1-{p}_{A}\left){p}_{B}\right),\mathrm{i}\mathrm{f}D>0,\\min({p}_{A}{p}_{B},(1-{p}_{A}\left)\right(1-{p}_{B}\left)\right),\mathrm{i}\mathrm{f}D<0.\end{array}$$

The squared correlation coefficient *r*^*2*^, which quantifies the degree of non-random association between loci, was calculated as:

$${r}^{2}=\frac{{D}^{2}}{{p}_{A}(1-{p}_{A}){p}_{B}(1-{p}_{B})}.$$

Pairwise LD values of the squared correlation coefficient (*r*^*2*^) were visualized as heatmaps using the R package LDheatmap^88^(version 0.99.8).

## Results

### Polymorphism of *MSTN* gene

**Fig. 1:**
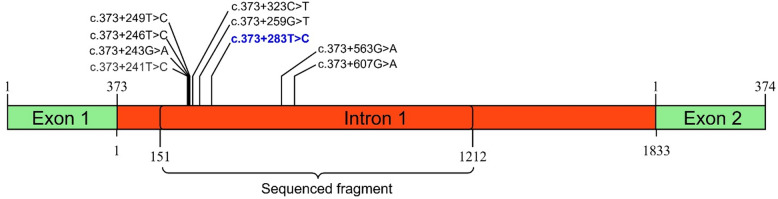
Fragment of the sheep *MSTN* gene. Exons are shown in green and the intron in red. The sizes of the exons, intron, sequenced region, and the positions of the identified SNPs are indicated. The figure was generated using the IBS 2.0 web server.

**Fig. 2:**
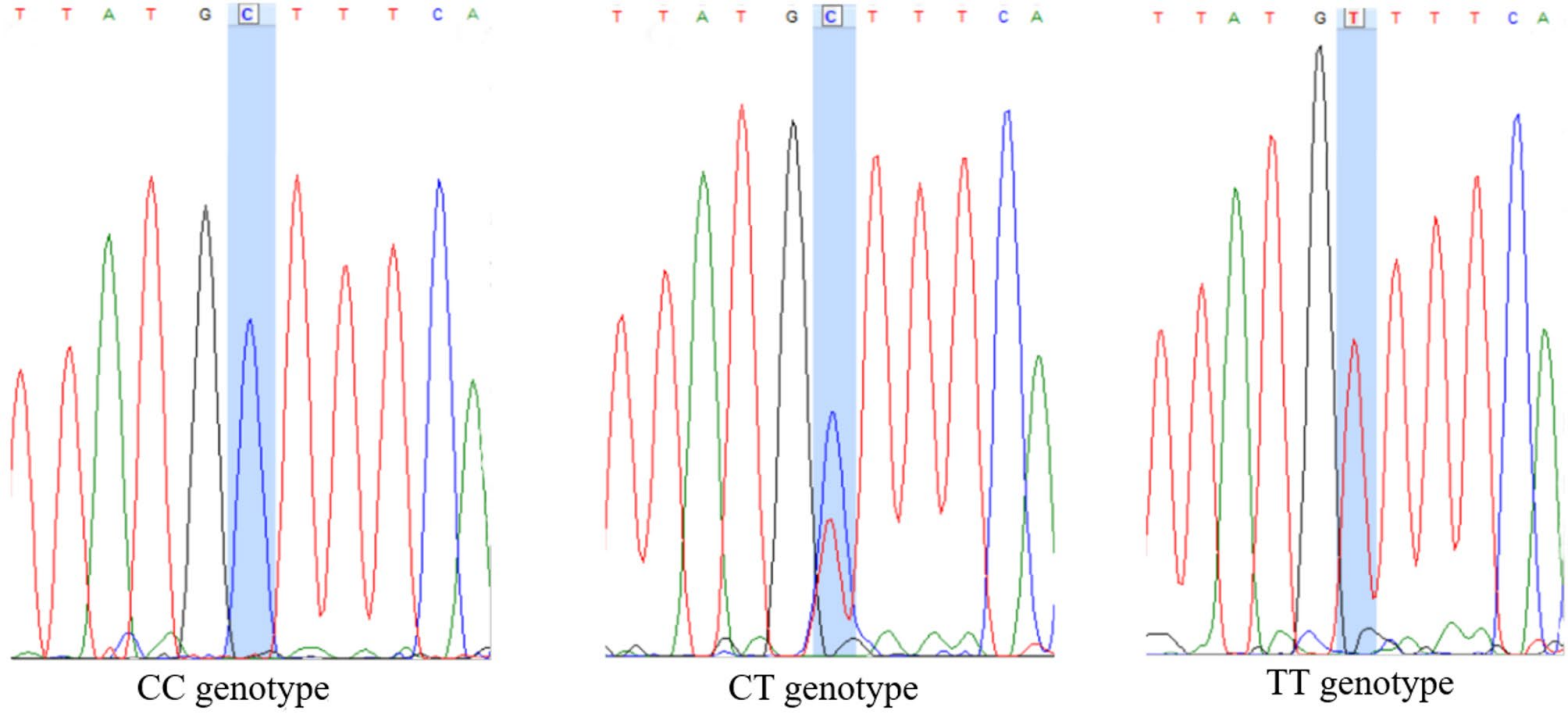
Identification of the novel SNP c.373+283T>C in intron 1 of the *MSTN* gene in UCM sheep using Sanger sequencing. Peaks on the chromatograms correspond to the CC, CT, and TT genotypes.

We sequenced a 1,062 bp fragment of the intron 1 of the *MSTN* gene in UCM sheep localized on chromosome 2, positions 119,285,858 to 119,292,614, according to the Ensembl 115 database (ENSOARG00020077637). Analysis of this region revealed the presence of multiple SNPs, indicating that the intron 1 is polymorphic in the studied population (Fig. 1). We identified eight previously known polymorphisms rs119102826 (c.373+241T>C), rs427811339 (c.373+243G>A), rs406172342 (c.373+246T>C), rs417602601 (c.373+249T>C), rs119102828 (c.373+259G>T), rs407388367 (c.373+323C>T), rs408710650 (c.373+563G>A), rs419902890 (c.373+607G>A) and one novel polymorphism localized 283 bp downstream of exon 1 (c.373+283T>C), that was not reported in the Ensembl 115 database. Data on polymorphisms detected in this study were deposited in European Variation Archive (project accession ID PRJEB98010)^89^. The novel variant was registered as rs7600797830. Sequencing chromatograms illustrating the newly detected polymorphism are presented in Fig. 2.

Conversely, none of the studied samples carried the alternative alleles for 11 other polymorphisms listed in Ensembl 115 in the sequenced region: rs3481344776 (c.373+244T>C), rs3481704354 (c.373+248A>G), rs3481615282 (c.373+315T>C), rs418842954 (c.373+364T>C), rs3481344788 (c.373+405T>G), rs119102830 (c.373+435A>C), rs430078136 (c.373+485C>T), rs3481615311 (c.373+571A>T), rs3481704348 (c.373+577del), and rs3481689011 (c.373+580T>C). All samples were homozygous for the reference allele at these loci.

In most of the detected SNPs, the minor mutant allele occurred exclusively in heterozygous form with frequencies ranging from 0.02 to 0.13 across loci (Table 1). The only exception was the novel polymorphism c.373+283T>C, for which all three genotypes (TT, CT, CC) were observed, with corresponding frequencies 0.91, 0.07, and 0.02. For all loci, homozygous wild-type alleles were predominant, ranging from 0.87 for c.373+259G>T to 0.98 for several loci with extremely low minor allele frequency.

**Table 1:** Genetic diversity at SNP loci in the intron 1 of the *MSTN* gene in Ukrainian Carpathian Mountain sheep (*n* = 54).

Variant	Genotypic frequency			Allelic frequency		H_o_	H_e_	F_IS_	PIC	χ^2^	*p*-value
rs119102826 (c.373+241T>C)	TT(0.98)	CT (0.02)	CC(0.00)	T(0.991)	C(0.009)	0.019	0.018	−0.009	0.0177	0.005	0.945
rs427811339 (c.373+243G>A)	GG(0.94)	AG(0.06)	AA(0.00)	G(0.972)	A(0.028)	0.056	0.054	−0.029	0.0530	0.044	0.834
rs406172342 (c.373+246T>C)	TT(0.98)	CT(0.02)	CC(0.00)	T(0.991)	C(0.009)	0.019	0.018	−0.009	0.0177	0.005	0.945
rs417602601 (c.373+249T>C)	TT(0.98)	CT (0.02)	CC(0.00)	T(0.991)	C(0.009)	0.019	0.018	−0.009	0.0177	0.005	0.945
rs119102828 (c.373+259G>T)	GG(0.87)	GT (0.13)	TT(0.00)	G(0.935)	T(0.065)	0.130	0.121	−0.069	0.1142	0.259	0.611
rs7600797830 (c.373+283T>C)	TT (0.91)	CT(0.07)	CC(0.02)	T(0.944)	C(0.056)	0.074	0.105	0.294	0.1001	4.671	0.031*
rs407388367 (c.373+323C>T)	CC(0.98)	CT(0.02)	TT(0.00)	C(0.991)	T(0.009)	0.019	0.018	−0.009	0.0177	0.005	0.945
rs408710650 (c.373+563G>A)	GG(0.98)	AG(0.02)	AA(0.00)	G(0.991)	A(0.009)	0.019	0.018	−0.009	0.0177	0.005	0.945
rs419902890 (c.373+607G>A)	GG(0.98)	AG(0.02)	AA(0.00)	G(0.991)	A(0.009)	0.019	0.018	−0.009	0.0177	0.005	0.945

Note. H_o_ - observed heterozygosity; H_e_ - expected heterozygosity under Hardy-Weinberg equilibrium. F_IS_ - inbreeding coefficient; PIC - polymorphic information content; χ^2^ - value corresponding to the deviation of observed genotype counts from expected under Hardy-Weinberg equilibrium; * indicates the statistical significance of the χ^2^ value at *p* < 0.05. Raw data used for statistics calculations is presented in Supplementary file 1.

The observed heterozygosity (H_o_) ranged between 0.019 and 0.130 for the majority of SNPs. It closely matched or slightly exceeded the expected heterozygosity (H_e_) in most cases, which varied from 0.018 to 0.121. The highest heterozygosity values were recorded for c.373+259G>T (H_o_ = 0.130) and c.373+283T>C (H_o_ = 0.074). The latter is notable for having a H_e_ value (0.105) higher than H_o_, indicating a heterozygote deficit. The Wright’s *F*_*IS*_ (inbreeding coefficient) was close to zero or slightly negative for all SNPs except c.373+283T>C, which had a positive value (*F*_*IS*_ = 0.294), suggesting some deficit of heterozygotes at this site.

PIC values were low across all loci (0.0177–0.1142), reflecting the low genetic diversity of this intron region in the studied population. The χ^2^ test for HWE indicated that eight loci out of nine conformed to HWE expectations, with χ^2^ values between 0.005 and 0.259. In contrast, the novel SNP c.373+283T>C deviated significantly from HWE (χ^2^ = 4.671, *p* < 0.05), showing a heterozygote deficit (H_o_ < H_e_) and a positive *F*_*IS*_ value. Given the low minor allele frequency and the relatively small sample size, this pattern may reflect stochastic sampling effects or population substructure, while the contributions of selection or genetic drift cannot be excluded.

**Fig. 3:**
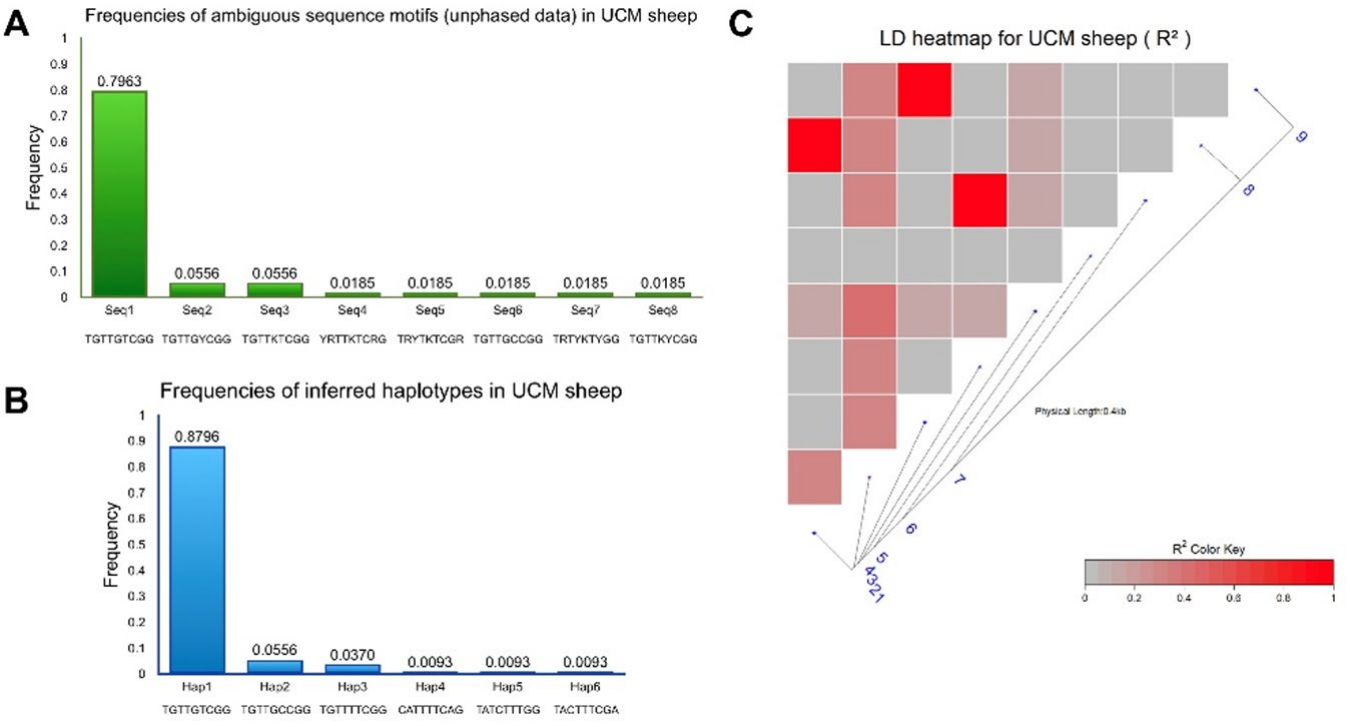
Analysis of allelic combinations and linkage disequilibrium patterns in UCM sheep. **(A)** Frequencies of ambiguous sequence motifs (unphased genotypes) identified in the UCM sheep. **(B)** Frequencies of inferred haplotypes reconstructed from phased genotypic data for the same individuals. **(C)** LD heatmap showing pairwise r^2^ values among the nine polymorphisms within intron 1 of the *MSTN* gene. Polymorphisms labeled as 1–9 correspond respectively to c.373+241T>C, c.373+243G>A, c.373+246T>C, c.373+249T>C, c.373+259G>T, c.373+283T>C, c.373+323C>T, c.373+563G>A, and c.373+607G>A.

When diploid genotype data for each animal were represented as sequence motifs of nine nucleotides (using ambiguity-coded nucleotides at heterozygous positions), it was possible to assess the diversity and prevalence of genotype combinations in unphased data. A total of eight distinct sequence motifs were identified in the sample (Fig. 3A), with the reference motif Seq1 (TGTTGTCGG) accounting for 79.63% of cases. This proportion reflects animals homozygous for the reference (major) alleles at all polymorphic sites.

After haplotype inference from unphased diploid data, six distinct haplotypes (Hap1–Hap6) were reconstructed. Haplotype frequencies in UCM sheep are shown in Fig. 3B, and Table 2 presents their distribution together with comparative data from other breeds^8,37,90^. Hap1, containing the reference allele at all variable positions, was the predominant haplotype, representing 87.96% of the population. The frequency of Hap1 exceeded that of Seq1 because phasing separated additional reference-carrying motifs present on one of the homologous chromosomes in heterozygous individuals. Hap2, with a frequency of 5.56%, differed from Hap1 only at the novel polymorphism site c.373+283T>C, carrying the C allele instead of the reference T. Hap3, occurring in 3.70% of the population, showed a single difference from Hap1 at c.373+259G>T, where the variant T allele was present. All of the remaining three haplotypes (Hap4-Hap6) occurred at a frequency of 0.93%. Hap4, Hap5, and Hap6 were characterized by the presence of four different mutations (Table 2). Overall, the UCM population shows a strongly skewed haplotype spectrum, with a dominant reference haplotype (Hap1) and only a small number of low-frequency derived haplotypes. This pattern is consistent with a low-diversity intron 1 profile and provides a concise contrast to breeds in which multiple non-reference haplotypes are reported at higher cumulative frequencies (Table 2)^8,37,90^.

**Table 2:** *MSTN* gene haplotypes found within different sheep breeds.

Breed	Haplotype	Frequency, %	rs119102826	rs427811339	rs406172342	rs417602601	rs119102828	rs7600797830	rs407388367	rs408710650	rs419902890	Reference
			c.373+241T>C	c.373+243G>A	c.373+246T>C	c.373+249T>C	c.373+259G>T	c.373+283T>C	c.373+323C>T	c.373+563G>A	c.373+607G>A	
Ukrainian Carpathian Mountain	Hap1	87.96	T	G	T	T	G	T	C	G	G	Curent data
	Hap2	5.56	T	G	T	T	G	C	C	G	G	
	Hap3	3.70	T	G	T	T	T	T	C	G	G	
	Hap4	0.93	C	A	T	T	T	T	C	A	G	
	Hap5	0.93	T	A	T	C	T	T	T	G	G	
	Hap6	0.93	T	A	C	T	T	T	C	G	A	
Latvian dark-headed	H1/H9	51/2,1	T	G	T	T	G	n	n	G	G	^8^
	H2	14,6	C	A	T	T	T	n	n	A	A	
	H3/H13	15,6/1	T	G	T	T	T	n	n	G	G	
	H4	8,3	T	A	C	T	T	n	n	G	A	
	H5	1	T	A	C	C	T	n	n	G	A	
	H7	2,1	C	A	T	T	G	n	n	A	G	
	H8	5,2	T	A	C	T	G	n	n	G	A	
Colored Polish Merino	MSTN-A	87.7	T	G	T	T	G	n	C	n	n	^37^
	MSTN-C	2.3	T	A	T	C	T	n	T	n	n	
	MSTN-E/E1	5.5/4.5	T	G	T	C	T	n	T	n	n	
New Zealand Romney	Hap A	68.3	T	G	T	T	G	n	C	n	n	^90^
	Hap B	22.7	C	A	T	T	T	n	C	n	n	
	Hap C	9	T	A	T	C	T	n	T	n	n	
	Hap D	< 1	T	A	C	T	T	n	C	n	n	
	Hap E	< 1	T	G	T	T	T	n	C	n	n	

Note. Comparative data highlight differences in the predominance of the reference haplotype and the breadth of the minor-haplotype spectrum across breeds.

LD between polymorphisms was evaluated using both D′ and r^2^ statistics. Because of the limited sample size, the absolute D′ value for all polymorphism pairs was equal to 1, which limited its interpretative power. The resulting r^2^ values are summarized in Table 3 together with their corresponding *p*-values obtained from Fisher’s exact test. Visualization of pairwise LD based on r^2^ values is shown as a heatmap in Fig. 3C. According to the r^2^ estimates, three pairs of polymorphisms exhibited evidence of LD: c.373+241T>C (rs119102826) and c.373+563G>A (rs408710650); c.373+246T>C (rs406172342) and c.373+607G>A (rs419902890); c.373+249T>C (rs417602601) and c.373+323C>T (rs407388367). LD between c.373+241T>C and c.373+563G>A is shown in Hap4; between c.373+246T>C and c.373+607G>A in Hap6; and between c.373+249T>C and c.373+323C>T in Hap5. The novel polymorphism rs7600797830 showed no LD with any of the previously characterized polymorphisms.

**Table 3:** Pairwise LD (r^2^) matrix for *MSTN* polymorphisms in UCM sheep.

	rs119102826	rs427811339	rs406172342	rs417602601	rs119102828	rs7600797830	rs407388367	rs408710650	rs419902890
rs119102826		0.327 (0.028)	0.000 (1.000)	< 0.001 (1.000)	0.135 (0.065)	0.001 (1.000)	< 0.001 (1.000)	**1.000** **(0.009)**	< 0.001 (1.000)
rs427811339	0.327 (0.028)		0.327 (0.028)	0.327 (0.028)	0.412 (< 0.001)	0.002 (1.000)	0.327 (0.028)	0.327 (0.028)	0.327 (0.028)
rs406172342	0.000 (1.000)	0.327 (0.028)		< 0.001 (1.000)	0.135 (0.065)	0.001 (1.000)	< 0.001 (1.000)	< 0.001 (1.000)	**1.000** **(0.009)**
rs417602601	< 0.001 (1.000)	0.327 (0.028)	< 0.001 (1.000)		0.135 (0.065)	0.001 (1.000)	**1.000** **(0.009)**	< 0.001 (1.000)	< 0.001 (1.000)
rs119102828	0.135 (0.065)	0.412 (< 0.001)	0.135 (0.065)	0.135 (0.065)		0.004 (1.000)	0.135 (0.065)	0.135 (0.065)	0.135 (0.065)
rs7600797830	0.001 (1.000)	0.002 (1.000)	0.001 (1.000)	0.001 (1.000)	0.004 (1.000)		0.001 (1.000)	0.001 (1.000)	0.001 (1.000)
rs407388367	< 0.001 (1.000)	0.327 (0.028)	< 0.001 (1.000)	**1.000** **(0.009)**	0.135 (0.065)	0.001 (1.000)		< 0.001 (1.000)	< 0.001 (1.000)
rs408710650	**1.000** **(0.009)**	0.327 (0.028)	< 0.001 (1.000)	< 0.001 (1.000)	0.135 (0.065)	0.001 (1.000)	< 0.001 (1.000)		< 0.001 (1.000)
rs419902890	< 0.001 (1.000)	0.327 (0.028)	**1.000** **(0.009)**	< 0.001 (1.000)	0.135 (0.065)	0.001 (1.000)	< 0.001 (1.000)	< 0.001 (1.000)	

Note. Each cell shows the pairwise linkage disequilibrium coefficient (r^2^) with the corresponding *p*-value from Fisher’s exact test given in parentheses. Estimates that are both in strong linkage disequilibrium and statistically significant are shown in bold.

### Effects of detected SNPs on pre-mRNA folding

The impact on the structure and stability of pre-mRNA of all 9 detected SNPs in the *MSTN* intron 1 of the UCM sheep, including novel polymorphism c.373+283T>C, were evaluated. The results obtained using remuRNA and RNAsnp tools are presented in Tables 4 and 5, respectively.

**Table 4:** Effects of SNPs in the *MSTN* intron 1 of the UCM sheep on pre-mRNA structure evaluated using remuRNA.

SNP	MFE (wild-type)	MFE (mutated)	ΔMFE	H (wild-type: mutated)
rs119102826 (c.373+241T>C)	−352.3	−353.7	1.4	1.396
rs427811339 (c.373+243G>A)		−352.2	−0.1	0.08
rs406172342 (c.373+246T>C)		−353.537	1.237	3.673
rs417602601 (c.373+249T>C)		−352.1	−0.2	1.014
rs119102828 (c.373+259G>T)		−352.5	0.2	0.773
rs7600797830 (c.373+283T>C)		−353.8	1.5	1.726
rs407388367 (c.373+323C>T)		−354.4	2.1	3.216
rs408710650 (c.373+563G>A)		−351.1	−1.2	1.456
rs419902890 (c.373+607G>A)		−350.537	−1.763	3.644

**Table 5:** Effects of SNPs in the *MSTN* intron 1 of the UCM sheep on pre-mRNA structure evaluated using RNAsnp.

SNP	Folding region	MFE (wild-type)	MFE (mutated)	ΔMFE	Local region	Distance	*p*-value
rs119102826 (c.373+241T>C)	41–441	−95.40	−94.10	−1.30	192–241	0.0205	0.6524
rs427811339 (c.373+243G>A)	43–443	−95.40	−95.80	0.40	192–241	0.0000	0.9979
rs406172342 (c.373+246T>C)	46–446	−95.50	−92.60	−2.90	235–284	0.1610	0.1077
rs417602601 (c.373+249T>C)	49–449	−95.40	−93.70	−1.30	241–290	0.0746	0.3004
rs119102828 (c.373+259G>T)	59–459	−95.30	−95.30	0.00	241–290	0.0120	0.7577
rs7600797830 (c.373+283T>C)	83–483	−98.70	−101.70	3.00	275–324	0.0107	0.7768
rs407388367 (c.373+323C>T)	123–523	−98.80	−97.10	−1.70	181–325	0.0653	0.3397
rs408710650 (c.373+563G>A)	363–763	−84.70	−84.30	−0.40	516–627	0.0045	0.8816
rs419902890 (c.373+607G>A)	407–807	−78.70	−76.60	−2.10	565–614	0.3010	0.0251

The obtained results from remuRNA indicate that the largest changes in relative entropy H within the pre-mRNA region corresponding to intron 1 are caused by the polymorphisms c.373+246T>C, c.373+323C>T, and c.373+607G>A. Moderate structural changes, as reflected by relative entropy H, may be expected for polymorphisms c.373+241T>C, c.373+283T>C, and c.373+563G>A. These same polymorphisms are also associated with notable changes in MFE: c.373+241T>C, c.373+246T>C, c.373+283T>C, and c.373+323C>T are predicted to stabilize the nucleic acid structure (reflected by decreased MFE), while c.373+563G>A and c.373+607G>A are predicted to destabilize it, leading to increased MFE values (Table 4).

The RNAsnp results indicate that the polymorphism c.373+607G>A causes the most substantial changes in the local pre-mRNA structure, as evidenced by the calculated Euclidean distance between local structures (0.3010), and is statistically significant (*p* = 0.0251). The c.373+246T>C polymorphism also showed a moderate Euclidean distance of 0.1610, but it is statistically non-significant, (*p* = 0.1077). Unlike remuRNA, RNAsnp assesses stability changes not for the RNA within the entire intron 1 but within a defined folding region extending 200 nucleotides upstream and downstream of the polymorphism position (Table 5). Within this context, RNAsnp identified that the c.373+283T>C polymorphism stabilizes the RNA structure (ΔMFE > 0). In contrast, polymorphisms c.373+241T>C, c.373+246T>C, c.373+249T>C, c.373+323C>T, and c.373+607G>A were identified as those that destabilize the structure within their respective folding regions the most (ΔMFE < 0).

A comparison of the remuRNA and RNAsnp results revealed concordance in the direction of ΔMFE for polymorphisms c.373+249T>C, c.373+283T>C, c.373+563G>A, and c.373+607G>A. For the remaining polymorphisms, the predicted ΔMFE direction differed between two tools, reflecting differences in their computational approaches: remuRNA evaluates structure across the entire intron 1 sequence, whereas RNAsnp focuses on local folding regions, which may explain the observed discrepancies. Among the studied polymorphisms, c.373+607G>A and the newly characterized c.373+283T>C had the most pronounced effects on both RNA structure and stability. Predicted secondary structures for the folding regions corresponding to all studied polymorphisms, as assessed by RNAsnp, are provided in Supplementary file 2.

### Effects of detected SNPs on binding of transcription factors

A comprehensive approach was used to analyze the intron 1 sequence for the presence of transcription factor binding sites. First, transcription factors potentially involved in the regulation of *MSTN* gene expression were identified using the RegNetwork and TRRUST tools. The identification of their binding sites is biologically meaningful in this context. Subsequently, the JASPAR database and the FIMO tool were used to determine whether consensus or near-consensus binding sequences of these transcription factors are located within intron 1 of the sheep *MSTN* gene. Notably, this multi-step approach helped to reduce the number of false-positive predictions. The results of these predictions are presented in Supplementary file 3.

It was observed that consensus binding sequences were identified either on the positive or the negative DNA strand for certain transcription factors. In such cases, the FIMO results showed low *p*-values (< 0.0001) and *q*-values (< 0.1), indicating both a low probability of random occurrence and a controlled false discovery rate, respectively. For example, the binding sequence of FOXO1, GTAAACA, is located on the positive strand in the region spanning positions 620–626 from the beginning of intron 1. The E-box sequence CAGCTG, recognized by transcription factors Myf5, MyoD1, and myogenin (MYOG), was found in regions 1436–1441 and 1613–1618. Multiple regions on both the positive and negative strands were also identified as containing the TAAGTA/G motif, which is characteristic of NKX3-1 binding (positions: 473–478, 713–718, 1047–1052, 1168–1173, 1447–1452, and 1479–1484). The octamer sequence ATGCTAAT, which may serve as a potential binding site for POU3F2, is located on the positive strand in the region spanning positions 153–160.

None of the aforementioned potential binding sites are affected by the genetic polymorphisms identified during sequencing in this study of UCM sheep. However, the detected polymorphisms influence other sequences that partially overlap with the motif profiles of one or more of the studied transcription factors (Table 6). These sequences are associated with higher *p*-values and *q*-values due to their deviation from the optimal consensus motifs. For example, the T allele in the c.373+259G>T polymorphism forms a sequence resembling the NKX3-1 binding motif. Similarly, the simultaneous presence of the T alleles at c.373+246T>C and c.373+249T>C generates a sequence on the positive strand that resembles the target of NFIL3. In contrast, the presence of the C allele at c.373+249T>C leads to the formation of an additional site on the negative strand, whereas in the presence of C allele at c.373+246T>C, the corresponding binding site is not detected. Furthermore, the C allele at c.373+283T>C disrupts sequences similar to those recognized by HNF1A and FOXO1, which are preserved when the T allele is present at this position. The sequence GAAAACA, found at positions 281–287 on the positive strand is of particular interest. It differs from the FOXO1 consensus sequence GTAAACA by one nucleotide.

**Table 6:** Sites of potential transcription factors binding affected by SNPs in the *MSTN* intron 1 of the UCM sheep.

Transcription factor	Allele variants	Strand	Start	End	*p*-value	q-value	Matched Sequence
NKX3-1 (MA0124.1)	G259	absent in the corresponding region					
	T259	-	255	261	0.000703	0.47	AT**A**CTAA
NFIL3 (MA0025.1)	T246, T249	+	246	256	0.000316	0.546	**T**TA**T**ATAGTTT
	C249	+	246	256	0.000406	0.616	TTA**C**ATAGTTT
		-	243	253	0.000486	0.616	CTAT**G**TAACAC
	C246	absent in the corresponding region					
HNF1A (MA0046.1)	T283	+	275	288	0.000206	0.252	TGTTTATG**T**TTTCA
	C283	absent in the corresponding region					
FOXO1 (MA0480.3)	T283	-	281	287	0.000385	0.147	GAAA**A**CA
	C283	absent in the corresponding region					

Thus, although none of the identified polymorphisms directly alter high-confidence consensus transcription factor binding sites, several variants modify sequences that partially overlap with established motif profiles. Such alterations may influence weaker or context-dependent transcription factor interactions and could become functionally relevant under specific regulatory conditions. Moreover, these variants may acquire greater regulatory impact in the course of evolutionary sequence changes, whereby additional nucleotide substitutions in the surrounding region give rise to a complete consensus transcription factor binding motif.

The results of the cross-species multiple sequence alignment for the region containing the studied polymorphisms, which allows assessment of interspecies variability within the predicted motifs, are presented in Supplementary file 4. The alignment shows that the polymorphic sites c.373+241T>C, c.373+243G>A, c.373+246T>C, c.373+249T>C, and c.373+259G>T are located within regions conserved among ruminant species. In contrast, the polymorphic sites c.373+283T>C, c.373+323C>T, c.373+563G>A, and c.373+607G>A occur in regions conserved across a broader taxonomic range of species. Notably, for several sites (c.373+243G>A, c.373+259G>T, c.373+283T>C, c.373+323C>T, and c.373+563G>A), the minor alleles observed in sheep correspond to the reference alleles in other species. This pattern suggests that the nucleotide substitutions identified in sheep may exert comparable effects on transcription factor binding at the interspecies level.

### Prediction of miRNA formation from sheep *MSTN* intron 1

A contemporary in silico approach involving miRNAFold and miRBoost tools was applied to predict the formation of pre-miRNAs within the intron of the *MSTN* gene, as the combined use of these tools reduces the likelihood of false-positive predictions. Using miRNAFold, 28 different pre-miRNAs were predicted from the sequence of sheep *MSTN* intron 1. After verification using miRBoost, four of the predicted hairpins were identified as likely true pre-miRNAs, while the remaining 24 were classified as probable pseudo pre-miRNAs (Supplementary file 5). The sequences corresponding to the putative true pre-miRNAs span the regions 578–658, 1357–1429, 1359–1457, and 1593–1707. To determine whether these miRNA candidates correspond to previously reported miRNAs of sheep or other ruminants (goats, cattle), the predicted hairpin regions were compared with entries in the miRBase and miRNASNP-v4 databases. No annotated pre-miRNA loci were found within intron 1 of the *MSTN* genes of these ruminants; therefore, the four identified hairpins are considered putative novel intronic pre-miRNA candidates pending experimental validation.

**Fig. 4:**
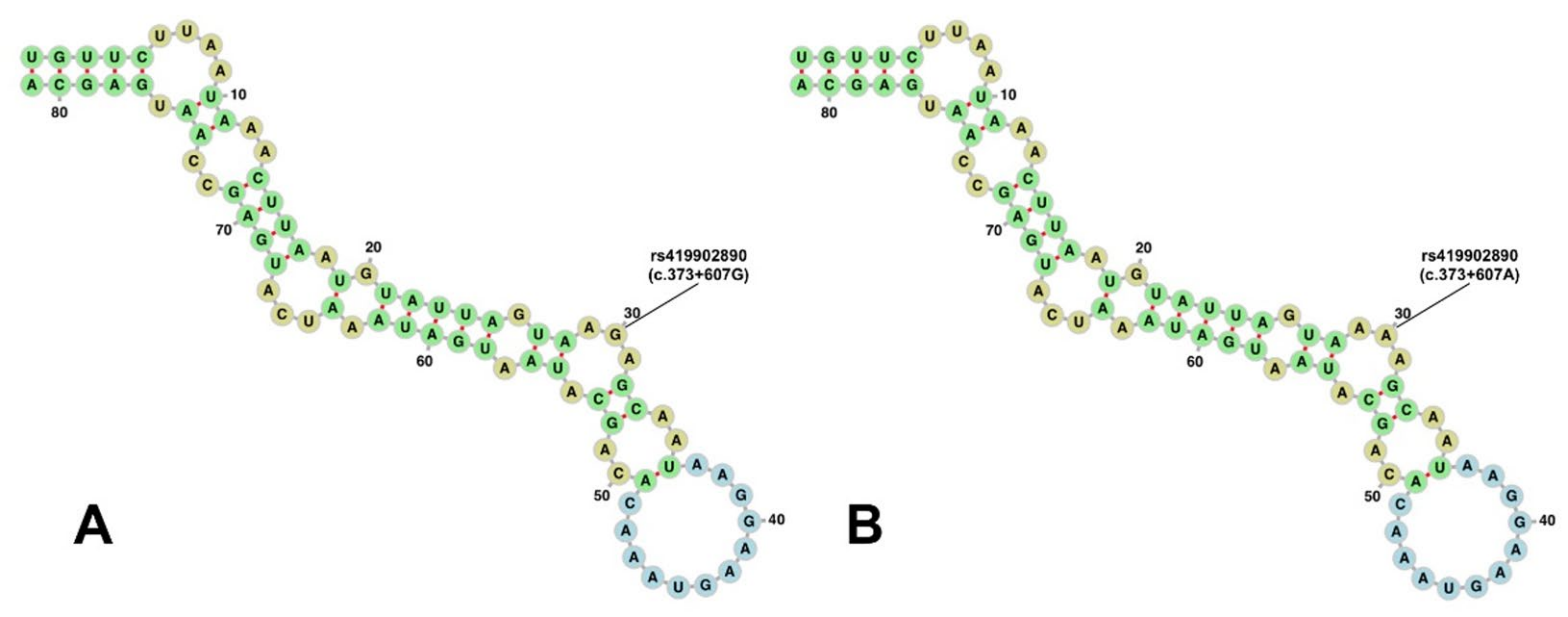
Predicted secondary structures of pre-miRNAs formed from intron 1 of the sheep *MSTN* gene. **(A)** Pre-miRNA carrying the reference G allele of the c.373+607G>A polymorphism. **(B)** Pre-miRNA carrying the mutant A allele of the c.373+607G>A polymorphism. Non-canonical base pairing between nucleotides at positions 11 and 74 was shown as originally predicted with miRNAFold. The depicted pre-miRNA corresponds to nucleotides 578–658 of intron 1 of the sheep *MSTN* gene, while the numbering shown in the figure refers to the 81-nucleotide pre-miRNA sequence itself and therefore starts from position 1.

The first of these hairpins encompasses the site of the c.373+607G>A polymorphism. Figure 4 presents the secondary structures of hairpins formed in the presence of different allelic variants at this position. The estimated MFE for pre-miRNA with both allelic variants is equal to −12.6 kcal/mol. Although the polymorphism does not disrupt base pairing and does not impact predicted MFE of pre-miRNA due to its location within the hairpin loop (Fig. 4), it may still influence the tertiary structure and properties of the nucleic acid.

**Fig. 5:**
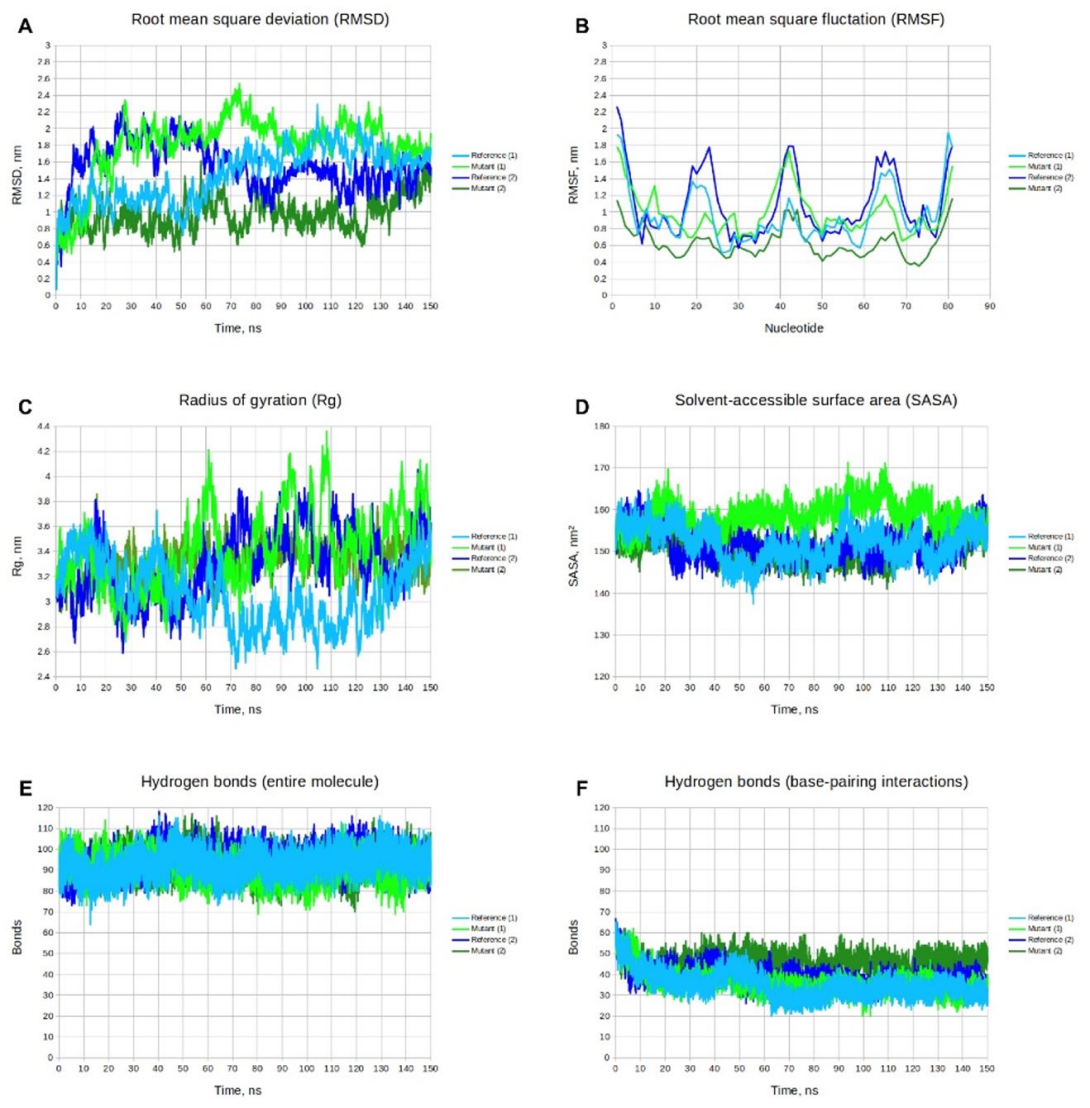
Analysis of molecular dynamics trajectories. **(A)** Root mean square deviation (RMSD). **(B)** Root mean square fluctuation (RMSF). **(C)** Radius of gyration (Rg). **(D)** Solvent-accessible surface area (SASA). **(E)** Number of hydrogen bonds within the entire RNA molecule. **(F)** Number of hydrogen bonds involving base-pairing atoms. Graphs for the pre-miRNA with the reference G allele of the c.373+607G>A polymorphism are shown in light blue and blue, and graphs for the pre-miRNA with the mutant A allele are shown in green and forest green. For each allele, the two color shades represent independent simulation replicates.

For the hairpin spanning nucleotides 578–658, two tertiary structures of pre-miRNAs differing at the c.373+607G>A polymorphic site were modeled. These structures were subjected to molecular dynamics simulations to assess the impact of the c.373+607G>A polymorphism on the stability and conformational behavior of the predicted pre-miRNA. For each molecule, two independent simulation replicates were performed to capture a broader range of possible structural changes. Figure 5 presents the key structural parameters obtained from the molecular dynamics analysis of the wild-type (G allele at c.373+607) and mutant (A allele at c.373+607) pre-miRNAs.

**Fig. 6:**
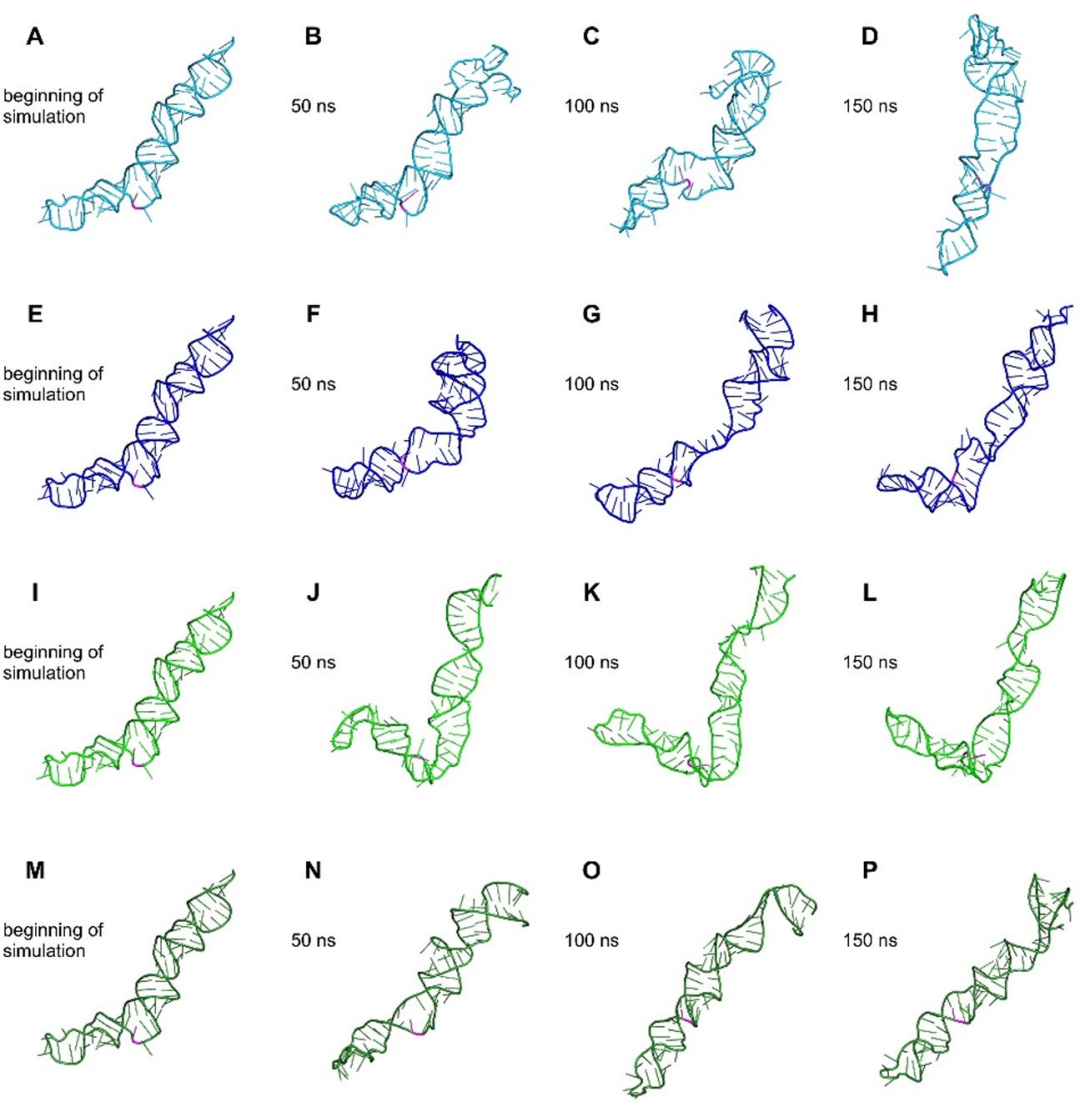
Conformational changes of pre-miRNAs during molecular dynamics simulations. Panels **A-H** show structures of the pre-miRNA carrying the reference G allele, where light blue and blue indicate conformational changes observed in two independent simulation replicates. Panels **I-P** show structures of the pre-miRNA carrying the mutant A allele, where green and forest green represent the corresponding two independent replicates. Polymorphic nucleotide is colored in magenta in all panels.

**Fig. 7:**
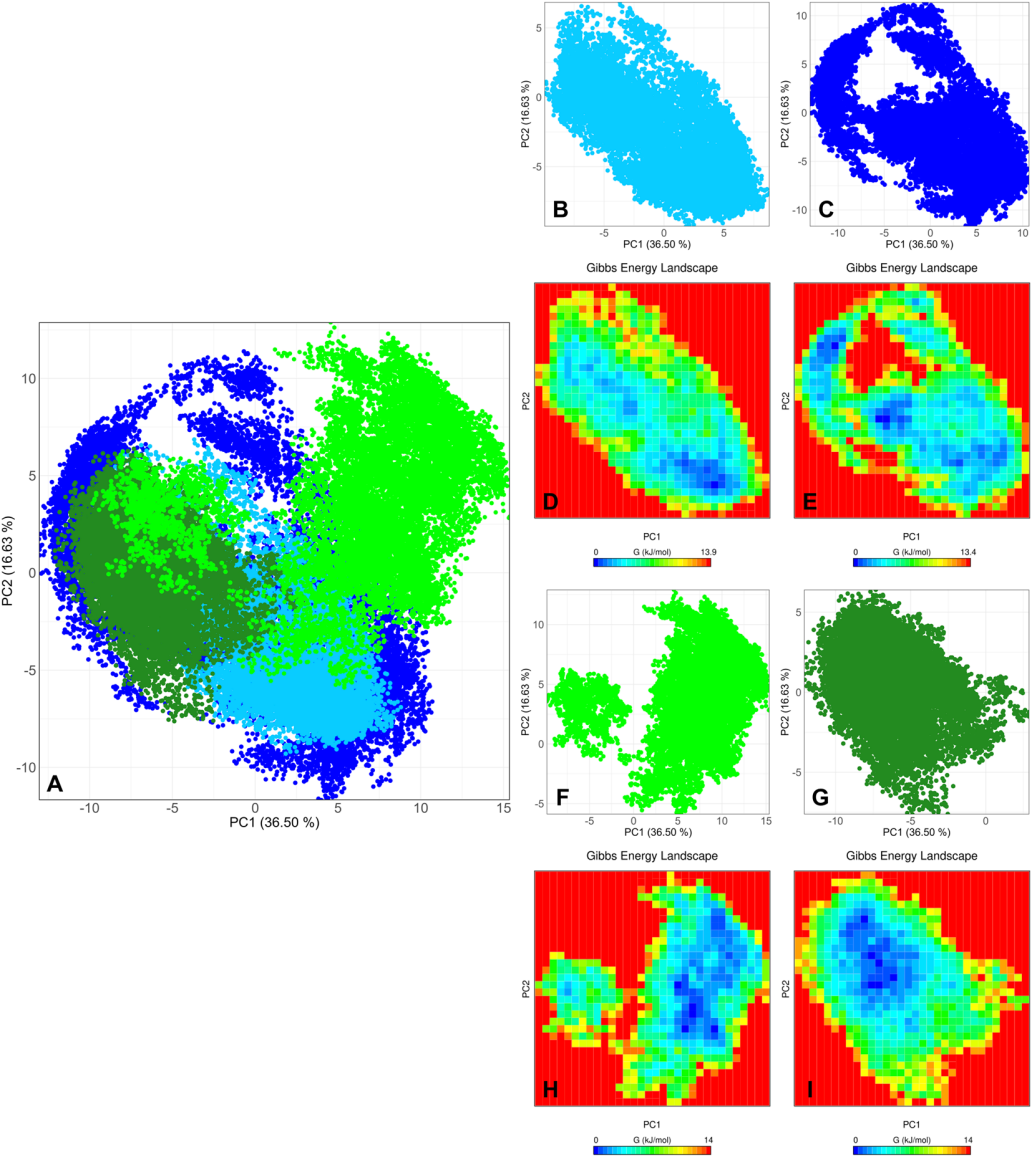
Principal component analysis (PCA) and free energy landscape (FEL) analysis based on molecular dynamics simulations of pre-miRNAs. **(A)** PCA projection onto the first two principal components including all simulation replicates of both pre-miRNAs: light blue and blue points represent the two replicates of the pre-miRNA carrying the reference G allele, while green and forest green points represent the two replicates of the pre-miRNA carrying the mutant A allele of the c.373+607G>A polymorphism. **(B-C)** separate PCA projections for two independent simulation replicates of the pre-miRNA with the reference G allele. **(D-E)** FEL plots corresponding to the same two replicates of the pre-miRNA with the reference G allele. **(F-G)** Separate PCA projections for two independent simulation replicates of the pre-miRNA with the mutant A allele. **(H–I)** FEL plots corresponding to the same two replicates of the pre-miRNA with the mutant A allele.

The molecular dynamics trajectory analysis included plotting root mean square deviation (RMSD), root mean square fluctuation (RMSF), radius of gyration (Rg), and solvent-accessible surface area (SASA), calculating the number of hydrogen bonds (Fig. 5), and performing principal component analysis (PCA) and free energy landscape (FEL) analysis (Fig. 7). The RMSD quantify the overall deviation of the molecular structure from its initial conformation over time and reflect global structural flexibility; lower and more consistent RMSD profiles across replicates indicate a more stable fold, whereas divergent RMSD patterns suggest structural heterogeneity. The RMSD plot (Fig. 5A) indicates high structural flexibility in both pre-miRNA molecules and notable deviations from their initial conformations over the course of the simulations. In the case of the pre-miRNA carrying the reference G allele, simulation 1 (light blue) and simulation 2 (blue) exhibited similar patterns of structural change during the second half of the trajectory. In contrast, for the pre-miRNA carrying the mutant A allele, simulation 1 (green) and simulation 2 (forest green) displayed pronounced differences in their dynamic behavior throughout the entire simulation, suggesting the existence of distinct conformational states (Figs. 5A and 6).

The RMSF measures the average mobility of individual nucleotides during the simulation; regions with high RMSF values correspond to flexible segments. The RMSF plot (Fig. 5B) shows that the most flexible regions of both pre-miRNAs correspond to the terminal segments and to hairpin loops lacking stable hydrogen-bonded base pairs, including the central hairpin (nucleotides 37–48, as annotated in Fig. 4). For the pre-miRNA carrying the reference G allele, simulations 1 (light blue) and 2 (blue) exhibited similar RMSF profiles across most regions, except for the central hairpin, which was less flexible in the first simulation than in the second (Fig. 5B). For the pre-miRNA carrying the mutant A allele, the replicate simulations showed pronounced differences in flexibility: the structure in simulation 1 (green) had higher RMSF values than that in simulation 2 (forest green), with the largest deviations also observed in the central hairpin region (Fig. 5B).

The Rg reflects the compactness of the tertiary structure, while SASA quantifies the exposure of nucleic acid atoms to solvent. Analysis of the Rg plot (Fig. 5C) indicates that the pre-miRNA carrying the reference G allele adopts a more compact conformation, with average Rg values (mean ± SD) of 3.06 ± 0.25 nm and 3.30 ± 0.26 nm in simulations 1 (light blue) and 2 (blue), respectively. In contrast, the pre-miRNA carrying the mutant A allele displays a less compact structure, with Rg values of 3.41 ± 0.30 nm and 3.32 ± 0.15 nm for simulations 1 (green) and 2 (forest green), respectively. Examination of the SASA plots (Fig. 5D) shows that the average SASA values (mean ± SD) were 151.89 ± 4.08 nm^2^ and 151.65 ± 3.48 nm^2^ for the pre-miRNA with the reference G allele in simulations 1 (light blue) and 2 (blue), respectively. For the mutant A allele, the average SASA value in simulation 2 (forest green) was 150.80 ± 3.80 nm^2^, which was comparable to that of the reference structure, whereas in simulation 1 (green) it increased to 158.72 ± 3.28 nm^2^, reflecting greater structural expansion and diffuseness.

Conformational changes of each pre-miRNA at 50 ns intervals are visualized in Fig. 6, clearly illustrating the differences in the adopted conformations of the two molecules and their relative compactness. Distinct conformational behavior was observed for the pre-miRNA carrying the mutant A allele in simulation 1 (green), which exhibited a characteristic bend in the structure (Fig. 6I-L) that was not present in the conformational states observed in any of the other simulations.

The high mobility of the studied molecules and the fluctuations that lead to the adoption of different conformational states also affected the number of intramolecular contacts. The average number (mean ± SD) of internal hydrogen bonds across the entire pre-miRNA carrying the reference G allele was 92.93 ± 6.19 in simulation 1 (light blue) and 94.63 ± 5.78 in simulation 2 (blue) (Fig. 5F). For the pre-miRNA carrying the mutant A allele, the corresponding values were 91.06 ± 5.82 in simulation 1 (green) and 93.12 ± 5.78 in simulation 2 (forest green) (Fig. 5F).

The average number (mean ± SD) of hydrogen bonds involving only base-pairing atoms within the pre-miRNA with reference G allele was 35.11 ± 6.16 in simulation 1 (light blue) and 38.74 ± 4.65 in simulation 2 (blue) (Fig. 5F). For the pre-miRNA with mutant A allele, the corresponding values were 35.99 ± 5.69 in simulation 1 (green) and 45.97 ± 4.19 in simulation 2 (forest green) (Fig. 5F).

During the simulations, the number of hydrogen bonds involving base-pairing atoms decreased at the beginning, whereas the total number of intramolecular hydrogen bonds in the entire molecule did not show such early fluctuations. This indicates a rearrangement of the hydrogen-bond network during molecular dynamics and stabilization of the molecule through intramolecular interactions rather than base-pairing. Notably, in simulation 2 of the pre-miRNA carrying the mutant A allele, the decrease in the number of base-pairing hydrogen bonds was smaller compared to the other simulations, resulting in a higher number of such bonds. In contrast, simulation 1 of the same mutant molecule displayed a different pattern, exhibiting the lowest number of total internal hydrogen bonds, which reflects reduced intramolecular cohesion due to the adopted bent conformation (Fig. 6).

PCA reduces the complex motion of the molecule into dominant collective movements, and FEL plots summarize the relative stability of sampled conformations. Together, these analyses illustrate the range of major conformational states and their energetic favorability, with distinct low-energy basins suggesting stable conformational preferences. In this study, PCA and FEL plots (Fig. 7) also show differences in the predominant conformational states of the two pre-miRNA molecules. The projections of positional fluctuations of the ribose C1′ atoms onto the first two principal components account for 36.50% and 16.63% of the total variance, respectively. A substantial overlap is observed between the PCA plots from simulation 1 (light blue) and simulation 2 (blue) of the pre-miRNA carrying the reference G allele (Fig. 7A-C). It indicates closely related positional fluctuations in the two independent replicates. At the same time, the PCA distribution for simulation 2 (Fig. 7C) spans a broader region of the coordinate space than that for simulation 1 (Fig. 7B), suggesting greater structural variability in the second replicate. The corresponding FEL plots (Fig. 7D-E) show separate low-energy regions for the two replicates, indicating that each simulation favors different projected conformational minima despite the overall similarity in positional fluctuations.

For the pre-miRNA carrying the mutant A allele, the PCA plots showed markedly different behavior. Only the PCA distribution from simulation 2 (Fig. 7A, G) overlaps with the PCA clusters observed for the structure with reference G allele (Fig. 7B-C). In contrast, the PCA plot for simulation 1 (Fig. 7F) displays an entirely distinct distribution of positional fluctuations, clearly separating this trajectory from both the second replicate of the same mutant molecule and from all simulations of the reference allele. The FEL plots for the mutant A allele (Fig. 7H-I) further support this observation by showing different energy minima in two independent simulations. Taken together, these analyses indicate that the pre-miRNA carrying the reference G allele exhibits broadly similar dynamical behavior across replicates (despite differences in the detailed energy landscape), whereas the pre-miRNA carrying the mutant A allele adopts completely different conformational states in repeated simulations. Distinct low-energy basins in the FEL and non-overlapping PCA clusters indicate that the same pre-miRNA can adopt different stable conformations. These conclusions are consistent with the structural visualization shown in Fig. 6.

## Discussion

The *MSTN* gene plays a critical role in the physiology of sheep and other animals. Mutations in this gene often exert a causal effect, directly influencing phenotypic traits, particularly the double-muscled phenotype^11,30^. Beyond its canonical function as a negative regulator of skeletal muscle growth, MSTN is increasingly recognized as a broader modulator of systemic physiology. Accumulating evidence indicates that altered MSTN signaling can influence glucose and lipid metabolism, whole-body energy homeostasis, and related metabolic pathways, supporting the notion that genetic variation in *MSTN* gene may exert indirect metabolic effects in addition to muscle-specific outcomes^91–93^. In livestock-relevant settings, perturbations of MSTN activity have also been associated with shifts in gut microbial composition^19–23^.

MSTN has additionally been implicated in the regulation of muscle development under hypoxic conditions. Oxygen-dependent upregulation of *MSTN* expression has been reported in hypoxia, with potential consequences for muscle growth and adaptation, a mechanism that may be particularly relevant in high-altitude or mountainous environments^94,95^. Such conditions are characteristic of mountain sheep breeds, including the UCM sheep, although the contribution of *MSTN* variation to hypoxia-related adaptation in these populations remains insufficiently explored. In a locally adapted mountain breed such as the UCM sheep, which exhibits resilience to cold, humidity, and other environmental stressors, the pleiotropic roles of *MSTN* are biologically meaningful, as they may intersect with multiple productivity- and adaptation-related traits.

According to Ensembl 115 release, 370 polymorphisms have been reported for sheep *MSTN* gene, 69 of which are located within its two introns, including 35 in intron 1. Several previous studies have examined polymorphisms in intron 1 of the *MSTN* gene in sheep, and associations between individual variants or haplotypes and productivity traits have been investigated^8,36,37,90,96^.

The present study is the first to examine polymorphisms in intron 1 of the *MSTN* gene in UCM sheep, a semi-coarse-wool breed with ancient origin. Our aim was to perform a comprehensive analysis, which included not only the identification of *MSTN* gene polymorphisms in this breed, but also a bioinformatic functional assessment to evaluate potential molecular mechanisms underlying their effects. In total, eight previously reported SNPs were identified in UCM sheep, together with a novel variant, c.373+283T>C, subsequently registered as rs7600797830.

It is noteworthy that the *MSTN* intron 1 sequence shows a high degree of evolutionary conservation among ruminants, with several regions remaining conserved even at broader, interspecies taxonomic levels. In particular, the positions harboring the polymorphisms c.373+241T>C, c.373+246T>C, and c.373+249T>C are conserved across ruminant species, indicating strong sequence constraint in these loci, whereas the position corresponding to c.373+607G>A is conserved across a wider range of mammalian species. At the same time, interspecies variability is evident at other polymorphic sites, and in several cases this variability mirrors the alternative allelic states observed within sheep. For example, in the reference genomes of pig (*Sus scrofa*) and human (*Homo sapiens*), a cytosine is present at the position corresponding to the newly identified sheep polymorphism c.373+283T>C, while the surrounding nucleotide motif remains identical to that of sheep (Supplementary file 4). This pattern of conservation of the local sequence context, combined with species-specific nucleotide variation at the focal position, provides additional support for the biological plausibility and genuine polymorphic nature of this site.

Interestingly, two SNPs located in the intron 1 of sheep *MSTN*, c.373+241T>C and c.373+563G>A, were observed together (Hap4) in this dataset and, given the high r^2^ value, may be in LD. This SNP pair was first described in the Latvian Dark-headed breed by Trapina et al.^9^ Consistent with current findings, Trapina et al.^8^ and Hickford et al.^90^ reported a transition c.373+241T>C in Latvian Dark-headed sheep (haplotypes H2 and H7) and New Zealand Romney sheep (haplotype Hap B), whereas no nucleotide variation at this position was detected in Colored Polish Merino^37^ or New Zealand Texel^53^. Notably, Latvian Dark-headed sheep with the genotypes c.373+241TT and c.373+563GG exhibit a significantly greater change in muscle depth at the 13th rib per kilogram of body weight gained during fattening^8^. Furthermore, in Egyptian sheep breeds (Barki, Ossimi, and Rahmani), the c.373+241TT genotype was statistically significant associated to high average daily gain (g/day)^97^.

The mutant allele A (c.373+243G>A), detected in the UCM breed at a frequency of 2.8%, was present in three haplotypes (Hap4, Hap5, and Hap6), each occurring at a frequency of less than 1%. Comparable results were reported for the Colored Polish Merino breed, in which allele A was found in a single haplotype (MSTN-C) with a frequency of 2.3%^37^. In contrast, in the Latvian Dark-headed breed, allele A was distributed across five haplotypes (H2, H4, H5, H7, and H8) with a combined frequency of 31.2%^8^, while in the New Zealand Romney breed it occurred in three haplotypes (Hap B, Hap C, and Hap D) with a combined frequency of approximately 32%^90^. In the Latvian Dark-headed breed, carriers of the mutant allele A (c.373+243G>A) exhibited higher body weight at 90 days of age. Furthermore, animals with the heterozygous genotype c.373+243GA had a greater proportion of slaughter weight relative to live weight at processing compared with individuals carrying the homozygous genotypes c.373+243GG or c.373+243AA^8^.

The c.373+246T>C and c.373+607G>A polymorphisms in UCM sheep appeared to be in LD, as suggested by a high r^2^ value, and were observed together in a single haplotype (Hap6) with a frequency of 0.93%. The C allele of c.373+246T >C occurs at a frequency of less than 1% in New Zealand Romney sheep, whereas in Colored Polish Merino and New Zealand Merino sheep it was detected only in the homozygous c.373+246TT genotype^37,53,90^. In contrast, these polymorphisms are more frequent in the Latvian Dark-headed breed: the C allele of c.373+246T>C is present in three haplotypes (H4, H5, H8) with a combined frequency of 14.5%, and the A allele of c.373+607G>A is found in four haplotypes (H2, H4, H5, H8) with a combined frequency of 29.1%. The presence of c.373+607G>A in H2, together with the absence of c.373+246T>C, indicates that the linkage between these two polymorphisms may not be complete. Both polymorphisms were associated with variation in body weight at 90 days of age in Latvian Dark-headed lambs^8^.

In UCM sheep, Hap5 was the only haplotype carrying a cytosine at position c.373+249 and a thymine at position c.373+323, with a frequency of 0.93%, and the high r^2^ value indicated the possibility of LD between these sites. In comparison, these polymorphisms were in LD in the Colored Polish Merino breed with a combined frequency of 12.3% (haplotypes MSTN-C, MSTN-E, MSTN-E1) and in New Zealand Romney sheep at 9% (haplotype Hap C)^37,90^. Trapina et al.^8^ reported the c.373+249T>C polymorphism in Latvian Dark-headed sheep at a frequency of 1%, but did not report c.373+323C>T, and no significant effect on growth traits was observed in this breed.

Regarding the newly identified polymorphism c.373+283T>C, which has a minor C allele frequency of 0.056, it was the only polymorphism in this study to show a statistically significant deviation from HWE. Given the sample size and the low allele frequency, the observed deviation from HWE may be driven by hidden population structure rather than by a locus-specific biological mechanism. Nevertheless, we cannot exclude that local selection or genetic drift in a population with a small effective size has contributed to the genotype distribution at this site. Further investigation in larger samples is needed to validate this finding.

This SNP was detected in a single haplotype (Hap2) with frequency of 5.56%. The C allele of c.373+283T>C appeared to be the only allele in this haplotype that differed from the reference sequence and may not be linked to other polymorphisms. Notably, despite numerous previous investigations of intron 1 in other sheep breeds^8,36,37,90,96^, this polymorphism has not been reported. This observation suggests that c.373+283T>C may serve as a valuable phylogenetic marker specific to a particular sheep breed or group of breeds. Consequently, further research is warranted to examine genetically related breeds to UCM and other sheep populations in the Carpathian region, to determine the geographical distribution of this polymorphism. It should also be noted that the allele and haplotype frequencies reported here reflect the sampled UCM population. Population-level estimates may differ in larger cohorts, and further validation in expanded sample sets is warranted to confirm the overall distribution and linkage patterns of the identified polymorphisms.

Taken together, the comparative datasets suggest that UCM sheep exhibit a low-diversity pattern in *MSTN* intron 1, with most genetic variation confined to rare haplotypes and a clear predominance of the reference haplotype. In contrast, studies of other sheep breeds^8,37,90^ have reported intron 1 polymorphisms distributed across multiple haplotypes with higher combined frequencies of non-reference alleles. Because the non-reference haplotypes in the present study occur at low frequencies, their estimates are sensitive to sampling effects; therefore, the observed low diversity should be interpreted cautiously as a feature of the analyzed UCM cohort until confirmed in larger, multi-flock datasets to determine whether it is representative of the breed as a whole.

Several studies examining the influence of *MSTN* intron 1 polymorphisms on economically important traits in sheep focus on haplotype-based associations rather than on individual genotypes. For example, Grochowska et al.^37^ demonstrated that the MSTN-E1 haplotype, which carries mutations at three positions (c.373+249, c.373+259, c.373+323), was associated with body weight on the second day of life in Colored Polish Merino lambs. The same study also reported associations between *MSTN* haplotypes and slaughter traits in this breed. Specifically, the presence of the MSTN-E allele (carrying mutations at c.373+249, c.373+259, and c.373+323) was linked to higher fore shank weight and heavier hind shank cuts compared with genotypes lacking this allelic variant. In contrast, a study by Hickford et al.^90^ in New Zealand Romney sheep found no significant associations between *MSTN* intron 1 variation and mean birth weight, weaning weight, pre-weaning growth rate, or the weight of lambs at drafting age.

Although this study did not aim to associate the identified polymorphisms with specific performance traits in UCM sheep, an in silico functional assessment was conducted to explore potential molecular mechanisms through which these mutations might exert their effects. Since intronic mutations do not directly alter protein structure and function in the manner of missense polymorphisms, their influence may be less apparent and require a comprehensive, multifaceted evaluation^33^. In this study, three possible aspects of the functional impact of intronic polymorphisms were considered: their effect on the stability of the primary transcript, potential alterations in transcription factor binding sites, and possible localization within regions from which precursor microRNAs (pre-miRNAs) are synthesized.

With respect to the effect on primary transcript (pre-mRNA) stability, the most pronounced changes were observed for the polymorphisms c.373+283T>C and c.373+607G>A. For both polymorphisms, the predicted direction of ΔMFE was consistent between remuRNA and RNAsnp. The minor C allele at position c.373+283T>C was associated with a decrease in minimum free energy (ΔMFE > 0), suggesting a stabilization of the transcript structure. Conversely, the minor A allele at position c.373+607G>A was associated with higher MFE values (ΔMFE < 0) relative to the reference transcript sequence, which may indicate destabilization. Alterations in pre-mRNA stability can influence transcript functionality, particularly by affecting the splicing and, consequently, the level and pattern of gene expression.

Regarding the in silico analysis of transcription factor binding sites, the most notable findings were several E-box motifs (CAGCTG), which may serve as binding sites for the myogenic regulatory factors Myf5, MyoD1, and myogenin (MYOG), as well as a binding site for FOXO1 (GTAAACA). Myf5, MyoD1, MYOG and MSTN are all involved in muscle development and growth. Myf5, MyoD1, and MYOG, along with other factors, belong to the family of myogenic regulatory factors that control myogenesis. Myf5, MyoD1, and MYOG act as positive regulators of muscle growth, whereas MSTN functions as a key negative regulator of this process^98–100^. Previous studies have demonstrated that MyoD can bind to E-boxes in the *MSTN* promoter across multiple species, thereby enhancing promoter activity^101,102^. Similarly, FOXO1 has been implicated in regulating *MSTN* promoter activity through its binding^103–105^.

Some limitation in predicting transcription factor binding sites arises from the fact that the major resources used to identify potential regulators of *MSTN* expression (RegNetwork^62,63^, TRRUST^64^ and the JASPAR^65^ motif profile database) are primarily based on human and mouse data. Nevertheless, this constraint is less consequential for transcription factors with well-characterized and evolutionarily conserved binding motifs. The motifs identified in this study are located within the intronic region of the sheep *MSTN* gene and therefore require targeted validation to clarify their functional relevance in regulating *MSTN* expression, for example through electrophoretic mobility shift assays (EMSA)^106^ to confirm transcription factor binding, chromatin immunoprecipitation (ChIP)^107^ to assess in vivo occupancy, and reporter gene assays^108^ to evaluate allele-specific effects on transcriptional activity. Although none of the 9 polymorphisms analyzed in this study significantly affect these sites, they may be relevant for other intronic polymorphisms.

The potential formation of pre-miRNAs within the region encompassing the c.373+607G>A polymorphism was predicted using miRNAFold^68,69^ and miRBoost^70^. Molecular dynamics simulations were subsequently performed to evaluate structural fluctuations in the corresponding nucleic acid hairpin, as well as the polymorphism-induced changes in structural dynamics, conformational behavior, and intramolecular bonds.

The structural dynamics observed in the molecular dynamics simulations can be interpreted in the context of canonical miRNA biogenesis, in which hairpin recognition and processing depend not only on primary sequence but also on the three-dimensional and dynamic properties of the stem-loop structure. The Drosha-DGCR8 complex recognizes characteristic hairpin architectures within primary transcripts and cleaves them to generate pre-miRNAs, which are subsequently processed by Dicer to yield mature miRNAs^109^. Accordingly, increased structural heterogeneity or enhanced flexibility of stem-loop elements may modulate the efficiency of miRNA processing. In the present study, the pre-miRNA carrying the reference allele exhibited a comparatively consistent global fold and compactness across independent simulations, whereas the pre-miRNA carrying the mutant allele showed broader conformational variability and a higher degree of dynamical divergence. These allele-dependent differences in structural behavior may therefore influence the probability and efficiency with which a given hairpin is recognized and processed into a mature miRNA.

This study represents one of the early applications of molecular dynamics-based analyses of pre-miRNA structures to the functional assessment of intronic polymorphisms in animal genomes. We acknowledge that experimental validation is required to confirm the biogenesis of miRNAs from intron 1 of the *MSTN* gene. Nonetheless, even if the formation of the predicted miRNA is not confirmed experimentally, the results of the molecular dynamics analysis would remain valuable, as they can still be interpreted as reflecting the effect of the polymorphism on the local tertiary hairpin structure of the primary mRNA.

In the context of bioinformatic analysis, this study can be compared with that of Sjakste et al.^50^, which also examined the impact of polymorphisms in the 5′-UTR and intron 1 of the sheep *MSTN* gene on transcription factor binding and pre-mRNA structure. Separately, the present work extends the conceptual framework of “rational selective breeding design”^33^, originally proposed for prioritizing missense variants through integrated bioinformatic evaluation, to intronic polymorphisms. In this framework, bioinformatic evaluation serves as an initial step for identifying sequence variants that merit subsequent empirical investigation.

Building on this framework, the present study applies bioinformatic screening to prioritize intronic SNPs for potential experimental validation and future association studies. The analysis highlights the previously reported polymorphism c.373+607G>A and the novel polymorphism c.373+283T>C as promising candidates for further investigation based on their in silico predicted regulatory potential. In particular, both variants are predicted to affect pre-mRNA stability, while c.373+607G>A is additionally predicted to influence the structural properties of a pre-miRNA potentially formed in the corresponding genomic region. In contrast, the in silico predictions for the remaining analyzed polymorphisms were less conclusive. Nevertheless, their combined investigation may still be relevant, especially in the context of possible linkage disequilibrium among polymorphisms located within intron 1 of the sheep *MSTN* gene.

As this study did not aim to establish direct genotype-phenotype associations, the predictions should be interpreted as indicative rather than confirmatory. Therefore, the functional and regulatory mechanisms proposed here remain computational and require direct experimental validation in cellular or animal models. Nonetheless, by providing an integrated bioinformatic evaluation of *MSTN* intronic polymorphisms, this study establishes a robust and informative foundation for prioritizing variants in sheep populations, offering a valuable framework to guide future experimental functional and association studies.

## Conclusions

The present study provides the first analysis of *MSTN* intron 1 polymorphisms in UCM sheep, identifying eight previously described SNPs and one novel variant, rs7600797830 (c.373+283T>C). The detected polymorphisms show varying frequencies and haplotype patterns, some of which align with observations in other sheep breeds, indicating both shared and breed-specific genetic features. Bioinformatic analyses suggest that intronic polymorphisms could influence *MSTN* gene regulation through effects on pre-mRNA stability, proximity to transcription factor binding sites, and potential pre-miRNA formation, providing hypotheses about possible regulatory mechanisms. Both previously reported c.373+607G>A and novel c.373+283T>C polymorphisms emerge as candidates warranting further experimental validation and association studies. By integrating molecular genetics and bioinformatic evaluation, this study establishes a robust foundation and a valuable framework to guide future functional studies and association analyses of *MSTN* intronic variants in sheep populations.

## Supplementary Information

Below is the link to the electronic supplementary material.

Supplementary Material 1

Supplementary Material 2

Supplementary Material 3

Supplementary Material 4

Supplementary Material 5

## Data Availability

Data on single-nucleotide polymorphism detected in this study were deposited in European Variation Archive (EVA) under project accession ID PRJEB98010. All other relevant data are within the paper and its supporting information files.
