# Supplementary material for: Identification and bioinformatic functional analysis of novel and known polymorphisms in the myostatin gene of Ukrainian Carpathian Mountain sheep
Source: Sci Rep. 2026 Mar 23;16:14628. doi: 10.1038/s41598-026-44326-6 (PMC13153238; doi:10.1038/s41598-026-44326-6)
Supplement: Supplementary file 3 — Supplementary Material 3 [file 41598_2026_44326_MOESM3_ESM.pdf]

MSTN Intron 1 Cross-Species Multiple Alignment

Notes:
snp1 - rs119102826 (c.373+241T>C)
snp2 - rs427811339(c.373+243G>A)
snp3 - rs406172342 (c.373+246T>C)
snp4 - rs417602601 (c.373+249T>C)
snp5 - rs119102828 (c.373+259G>T)
snp6 - rs7600797830 (c.373+283T>C)
snp7 - rs407388367 (c.373+323C>T)
snp8 - rs408710650 (c.373+563G>A)
snp9 - rs419902890 (c.373+607G>A)
xx→intron 1 - intron 1 beginning (arrow correspond to first intronic nucleotide)

```
                              xx→intron 1
ovis_aries/1-2852             GTGTGAGTAGT--------TCTG----CTA----------GGGCAGAGC-----------
capra_hircus/1-2852           GTGTGAGTAGT--------TCTG----CTA----------GTGCAGAGC-----------
bison_bison_bison/1-2852      GTGTGAGTAGT--------CCTG----CTG----------GTGCAGAGC-----------
bos_grunniens/1-2852          GTGTGAGTAGT--------CCTG----CTG----------GTGCAGAGC-----------
bos_mutus/1-2852              GTGTGAGTAGT--------CTTG----CTG----------GTGCAGAGC-----------
bos_taurus/1-2852             GTGTGAGTAGT--------CCTG----CTG----------GTGCAGAGC-----------
sus_scrofa/1-2852             GTGTAAGTAGT--------CCTA----TTA----------GTGTATATC-----------
camelus_dromedarius/1-2852    GTGTAAGTAGT--------CCTA----TTA----------GTGTATATC-----------
canis_lupus_familiaris/1-2852 GAGTGAGTAGC----------------TGG----------GTGTGTGGC-----------
homo_sapiens/1-2852           GTGTAAGTAGT----CCTA--------TTA----------GTGTATATC-----------
mus_musculus/1-2852           GTGTAAGTATA----TCTG--------TTA----------AAGTATATC-----------
oryctolagus_cuniculus/1-2852  GTGTAAGTAGT----CCTA--------TTA----------GTGTATGTC-----------
                              * ** ****                                 *     *


ovis_aries/1-2852             ---------------------AACGACTCTGCTGACT----------------GCTGTTC
capra_hircus/1-2852           ---------------------AACGACTCTGCTGACT----------------GCTGTTC
bison_bison_bison/1-2852      ---------------------AACGACTCTGCTGACT----------------GCTGTTC
bos_grunniens/1-2852          ---------------------AACGACTCTGCTGACT----------------GCTGTTC
bos_mutus/1-2852              ---------------------AACGACTCTGCTGACT----------------GCTGTTC
bos_taurus/1-2852             ---------------------AACGACTCTGCTGACT----------------GCTGTTC
sus_scrofa/1-2852             ---------------------AACAATTCTGCTGACT----------------GTTGTTC
camelus_dromedarius/1-2852    ---------------------AACAATTCTGCTGACT----------------GTTGTTC
canis_lupus_familiaris/1-2852 -----------------------------------------------------GCGGGGC
homo_sapiens/1-2852           -----AACAGTTCTGCTGACT--------------------------------GTTGTTC
mus_musculus/1-2852           -----AACAGTTCTGCTGACT--------------------------------GCTGTCC
oryctolagus_cuniculus/1-2852  -----AACAGTTCTGCTGACT--------------------------------GTTGTTC
                                                                                   *  *  *


ovis_aries/1-2852             TAGTGTTCATGAGAAACCGA------------TCTATT------TTCAGGCTCTT-TTA-
capra_hircus/1-2852           TAGTGTTCATGAGAAACCGA------------TCTATT------TTCAGGCTCTT-TTA-
bison_bison_bison/1-2852      TAGTGTTCATGAGAAACCGA------------TCTATT------TTCAGGCTCTT-TTA-
bos_grunniens/1-2852          TAGTGTTCATGAGAAACCGA------------TCTATT------TTCAGGCTCTT-TTA-
bos_mutus/1-2852              TAGTGTTCATGAGAAACCGA------------TCTATT------TTCAGGCTCTT-TTA-
bos_taurus/1-2852             TAGTGTTCATGAGAAACCGA------------TCTATT------TTCAGGCTCTT-TTA-
sus_scrofa/1-2852             CAGTGTTTATGAGAAACAGA------------TCTATT------TTCAGGCTCTT-TTA-
camelus_dromedarius/1-2852    TAGTGTTTATGAGAAACAGA------------TCTATT------TTCAGGCTCTT-TTA-
canis_lupus_familiaris/1-2852 TCGTGTCGGTGG-CAGCTGG------------------------TCCGGGCTCCC-TTC-
homo_sapiens/1-2852           TAGTGTTTATGAGAAACAGA------TCTATT------------TTCAGGCTCTT-TTAA
mus_musculus/1-2852           TAGTGTTTATGAGAAACAGA------TCTATT------------TTCAGGCT--T-TT--
oryctolagus_cuniculus/1-2852  TAGTGTTTATGGGAAACAGA------TCTATT------------TTCAGGCTCTT-TTA-
                                ****   **   * * *                         * * ****    **


ovis_aries/1-2852             ACAAGCTGCT-----------GGCTTG----TA--CGTAAGGAGGAGGGC----A--AAG
capra_hircus/1-2852           ACAAGCTGCT-----------GGCTTG----TA--CGTAAGGAGGAGGGC----A--AAG
bison_bison_bison/1-2852      ACAAGCTGCT-----------GGCTTG----TA--TGTAAGGAGGAGGGG----A--GAG
bos_grunniens/1-2852          ACAAGCTGCT-----------GGCTTG----TA--TGTAAGGAGGAGGGG----A--AAG
bos_mutus/1-2852              ACAAGCTGCT-----------GGCTTG----TA--TGTAAGGAGGAGGGG----A--AAG
bos_taurus/1-2852             ACAAGCTGCT-----------GGCTTG----TA--TGTAAGGAGGAGGGG----A--AAG
sus_scrofa/1-2852             ACAAGCTGTT-----------GGCTTG----TA--CGTAAGTAGGAGGGA----A--AAG
camelus_dromedarius/1-2852    ACAGGCTGTT-----------CGTGTG----TA--TGTAAGTAGGAGGGA----A--AAG
canis_lupus_familiaris/1-2852 CCAGGCCGCC-----------GCCGTG--------TGGGAGTGGGAAGGA--------GG
homo_sapiens/1-2852           ACAAGCTGTT-----------GGCCTG--TA----TGTAAGTAGAAAGGA--AA----AG
mus_musculus/1-2852           ----------AACAAGCTGTTGGCTTA--TA----TGTAAGTAGCAGAGA--AA----GG
oryctolagus_cuniculus/1-2852  ATAAGCTGTC-----------AGCTTT--TA----TGTAAGAAGCAGGAA--AG----AC
                                                       *          *  **  * *
```

```
ovis_aries/1-2852             AGCTT--------------TTTG---------------CAAGACTTCATGAGA---AAT-
capra_hircus/1-2852           AGCTT-------------TTTTG---------------CAAGACTTCATGAGA---AAT-
bison_bison_bison/1-2852      AGCTT--------------TTTT---------------CAAGATTTCATGAGA---AAT-
bos_grunniens/1-2852          AGCTT--------------TTTT---------------CAAGATTTCATGAGA---AAT-
bos_mutus/1-2852              AGCTT--------------TTTT---------------CAAGATTTCATGAGA---AAT-
bos_taurus/1-2852             AGCTT--------------TTTT---------------CAAGATTTCATGAGA---AAT-
sus_scrofa/1-2852             AGTTTCTT-----------TTTT---------------CAAGATTTCATGAGA---AAT-
camelus_dromedarius/1-2852    AGTTTCAT-----------TTTT---------------CAAGATTTCATGAGA---AAT-
canis_lupus_familiaris/1-2852 ATCTCTGT---C-------TTTG---------------CAAGATGTCACGGAGCTGAGC-
homo_sapiens/1-2852           AGTTTCTC---T-------TTTT---------------CAAGATTGCATGAGA----AT-
mus_musculus/1-2852           AGATTTTT-----------TTTT------------CCCCAAGATTTCCTGAGA---AAC-
oryctolagus_cuniculus/1-2852  AGTCTCTT---C-------TTTT---------------CAAGATTTTGTGAGA---AAT-
                              *                  ***                *****      *


ovis_aries/1-2852             --------ATGCTAA------TGAGACTGAAAGCTGCTACAT------------------
capra_hircus/1-2852           --------ATGCTAA------TGAGACTGAAAGCTGCTACAT------------------
bison_bison_bison/1-2852      --------AGACCAA------TGAGACTGAAAGCTGCTACTT------------------
bos_grunniens/1-2852          --------AGACCAA------TGAGACTGAAAGCTGCTACTT------------------
bos_mutus/1-2852              --------AGACCAA------TGAGACTGAAAGCTGCTACTT------------------
bos_taurus/1-2852             --------AGACCAA------TGAGACTGAAAGCTGCTACTT------------------
sus_scrofa/1-2852             --------AAACTAA------TGAGACTGAAAGCTGCTGTATT-----------------
camelus_dromedarius/1-2852    -----------CTAA------TGAGACTGAGAGCTGCTGCATT-----------------
canis_lupus_familiaris/1-2852 -----------TGAA------GGACGCAGGAAGCTGCGGCACC-----------------
homo_sapiens/1-2852           ---AT-AT----------TAATGAGACAAAAATCTGCTGCAT------------------
mus_musculus/1-2852           ---AT-AC-------------CGACAATGAAATCTGTC---T------------------
oryctolagus_cuniculus/1-2852  ---AT-GT-------------TGAAACTGAAATCTGTCACAC------------------
                                                    **        * ***


ovis_aries/1-2852             --------------------------------TATCTGTTTCCTTA----GAGAGCT---
capra_hircus/1-2852           --------------------------------TATCTGTTTTCTTA----GAGAGCT---
bison_bison_bison/1-2852      --------------------------------TATTTGTTTCCTTA----GAGAGCG---
bos_grunniens/1-2852          --------------------------------TATTTGTTTCCTTA----GAGAGCT---
bos_mutus/1-2852              --------------------------------TATTTGTTTCCTTA----GAGAGCT---
bos_taurus/1-2852             --------------------------------TATTTGTTTCCTTA----GAGAGCT---
sus_scrofa/1-2852             -------------------------ATT--------GTTTTCCTTA--------GCT---
camelus_dromedarius/1-2852    -------------------------ATA--------TATTCCCTGAGGGA----GCT---
canis_lupus_familiaris/1-2852 ---------------------------------------------------------ATC
homo_sapiens/1-2852           ----TATTTGTTTTCTTATAGAGAC-----------------------------------
mus_musculus/1-2852           TGCT----TGTTTCCTGGCAGTGCC-----------------------------------
oryctolagus_cuniculus/1-2852  TGTC----TGTTTCCTTAAAAAGCT-----------------------------------


ovis_aries/1-2852             -----------------AAAAAGCTAAAAATC--AGA-AATGAAATGCTTGCAT------
capra_hircus/1-2852           -----------------AAAAAGCTAAAAATC--AGA-AATGAAATGCTCGCAT------
bison_bison_bison/1-2852      -----------------AAAAAGCTAAAAATC--AAA-AATGAAATGCTTGCAT------
bos_grunniens/1-2852          -----------------AAAAAGCTAAAAATC--AAA-AATGAAATGCTTGCAT------
bos_mutus/1-2852              -----------------AAAAAGCTAAAAATC--AAA-AATGAAATGCTTGCAT------
bos_taurus/1-2852             -----------------AAAAAGCTAAAAATC--AAA-AATGAAATGCTTGCAT------
sus_scrofa/1-2852             -----------------AAACAGCTGAAAATA--AAA-AATAAAATGCTTGCAT------
camelus_dromedarius/1-2852    -----------------AAAAAGCTAAAAATA--AAA-AATAAAATGCTTGCAT------
canis_lupus_familiaris/1-2852 GGCCTCGCCGGAGAGCTCAAAAGCCAAAAGGAGATGAGGATAAAACGCCTGCGT------
homo_sapiens/1-2852           -----------------AAAAAACTAAAA---------AATAAAGTACTTGCAT------
mus_musculus/1-2852           -----------------AAATAGCTCAAA---------GTTAATAT--------------
oryctolagus_cuniculus/1-2852  -----------------AAAAAGCTAAAA---------GATAAAAAGCTTGCAC------
                                                ** * *  ***           * *


ovis_aries/1-2852             ------------------------------------------------------------
capra_hircus/1-2852           ------------------------------------------------------------
bison_bison_bison/1-2852      ------------------------------------------------------------
bos_grunniens/1-2852          ------------------------------------------------------------
bos_mutus/1-2852              ------------------------------------------------------------
bos_taurus/1-2852             ------------------------------------------------------------
sus_scrofa/1-2852             ------------------------------------------------------------
camelus_dromedarius/1-2852    ------------------------------------------------------------
canis_lupus_familiaris/1-2852 ------------------------------------------------------------
homo_sapiens/1-2852           -----------AGCA--------------------------------------------T
mus_musculus/1-2852           ---------------------------GCTGGCTCAGCTTTAATAGTAT----------T
oryctolagus_cuniculus/1-2852  -----------AGCG------------------------TTAATATTAT----------C
```

```
                                                                                  snp1 snp2
ovis_aries/1-2852             --------------------------------------------------AGCATTCGTG
capra_hircus/1-2852           --------------------------------------------------AGCATTCATG
bison_bison_bison/1-2852      --------------------------------------------------AGCATTCATG
bos_grunniens/1-2852          --------------------------------------------------AGCATTCATG
bos_mutus/1-2852              --------------------------------------------------AGCATTCATG
bos_taurus/1-2852             --------------------------------------------------AGCATTCATG
sus_scrofa/1-2852             ---------------------------------AGCATT---GTTATATA----------
camelus_dromedarius/1-2852    ----------------AGCATTAATATTATATA---------------------------
canis_lupus_familiaris/1-2852 --GGCGCTCACGTTTC--------------------------------------------
homo_sapiens/1-2852           TA----------------------------------------------------------
mus_musculus/1-2852           TA----------------------------------------------------------
oryctolagus_cuniculus/1-2852  TA----------------------------------------------------------

                            snp3 snp4                               snp5(rs119102828)
ovis_aries/1-2852             TTATATA-----------------GTTTAG--------GATGAC-AA---------CTAT
capra_hircus/1-2852           TTATATA-----------------GTTTAG--------TATGAC-AA---------CTAT
bison_bison_bison/1-2852      TTATATA-----------------GTTTAG--------TATGAC-AA---------CTAT
bos_grunniens/1-2852          TTATATA-----------------GTTTAG--------TATGAC-AA---------CTAT
bos_mutus/1-2852              TTATATA-----------------GTTTAG--------TATGAC-AA---------CTAT
bos_taurus/1-2852             TTATATA-----------------GTTTAG--------TATGAC-AA---------CTAT
sus_scrofa/1-2852             ------------------------GTTTAATAAG--------AC-AA---------ATAT
camelus_dromedarius/1-2852    ------------------------GTTTAGCATG--------AC-CA-------------
canis_lupus_familiaris/1-2852 ------------------------GCTGCGTGTG--------AC-AGTGGC----G----
homo_sapiens/1-2852           ------------------------ATTTAATAAG--------GC-AAATATAGATA----
mus_musculus/1-2852           ------------------------ATTTTATGCG--------GA-AAATGT----A----
oryctolagus_cuniculus/1-2852  ------------------------GTTTAAT-TG--------GC-AAATAC----A----
                                                        *

                                            snp6(rs7600797830)
ovis_aries/1-2852             AAC--ATGTTTATGTTTTCACAGCTTAATGCTA----CCAA-GGTGA-------------
capra_hircus/1-2852           AAC--ATGTTTATGTTTTCACAGCTTAATGCTA----CCAA-GGTGA-------------
bison_bison_bison/1-2852      AAC--ATGTTTATGTTTTCACAGCTTAATGCTA----CCAA-GGTAA-------------
bos_grunniens/1-2852          AAC--ATGTTTATGTTTTCACAGCTTAATGCTA----CCAA-GGTAA-------------
bos_mutus/1-2852              AAC--ATGTTTATGTTTTCACAGCTTAATGCTA----CCAA-GGTAA-------------
bos_taurus/1-2852             AAC--ATGTTTATGTTTTCACAGCTTAATGCTA----CCAA-GGTAA-------------
sus_scrofa/1-2852             AAC--ATGCTTATGCTTTCACAGCTTAATGCCA----CCAA-GGC-A-------------
camelus_dromedarius/1-2852    -AT--ATGCTTATGTTTTCACAGCTTAATACCA----CCAA-GGC-A-------------
canis_lupus_familiaris/1-2852 -CC--GGGCTTGCACCCTCGCAGCGCGGTGGCC----GCGGTGGC-A-------------
homo_sapiens/1-2852           -GC--ATGCTTATGCTTTCACAA--TAATACCA----CCAA-GGC-A-------------
mus_musculus/1-2852           -GA--ATGCTTGTGTGTTCAGAGTCTCACACT-----ATAA-TGT-A-------------
oryctolagus_cuniculus/1-2852  -GC--ATGCTTATGCCTTCACAGCCTCATACCAACCAGCAA-GGC-G-------------
                                     * **      **  *                     *

ovis_aries/1-2852             ------------------------------------------------------------
capra_hircus/1-2852           ------------------------------------------------------------
bison_bison_bison/1-2852      ------------------------------------------------------------
bos_grunniens/1-2852          ------------------------------------------------------------
bos_mutus/1-2852              ------------------------------------------------------------
bos_taurus/1-2852             ------------------------------------------------------------
sus_scrofa/1-2852             ------------------------------------------------------------
camelus_dromedarius/1-2852    ------------------------------------------------------------
canis_lupus_familiaris/1-2852 ------------------------------------------------------------
homo_sapiens/1-2852           ------------------------------------------------------------
mus_musculus/1-2852           ------------------------------------------------------------
oryctolagus_cuniculus/1-2852  ------------------------------------------------------------

ovis_aries/1-2852             ------------------------------------------------------------
capra_hircus/1-2852           ------------------------------------------------------------
bison_bison_bison/1-2852      ------------------------------------------------------------
bos_grunniens/1-2852          ------------------------------------------------------------
bos_mutus/1-2852              ------------------------------------------------------------
bos_taurus/1-2852             ------------------------------------------------------------
sus_scrofa/1-2852             ------------------------------------------------------------
camelus_dromedarius/1-2852    ------------------------------------------------------------
canis_lupus_familiaris/1-2852 ------------------------------------------------------------
homo_sapiens/1-2852           --------AGGACTGGGAGATACTACAAGCAGTGTTTAAAACTTACATTAGATTTTAGAA
mus_musculus/1-2852           --------AGGACTGTGAGATTCTATAGGCAGTGTTAAAG-C--ATGAGAAA-TTTACAA
oryctolagus_cuniculus/1-2852  --------AGGACTGGCAGATACTAC-AGCAATGTTAAAAACTCACAGGGAATTTTATAA
```

ovis_aries/1-2852	--AG
capra_hircus/1-2852	--AG
bison_bison_bison/1-2852	--AG
bos_grunniens/1-2852	--AG
bos_mutus/1-2852	--AG
bos_taurus/1-2852	--AG
sus_scrofa/1-2852	--AG
camelus_dromedarius/1-2852	--AG
canis_lupus_familiaris/1-2852	--
homo_sapiens/1-2852	TTGTATTTAGTT-------GTGTAAAAT--AAGT---------------TT------T--
mus_musculus/1-2852	TTACATTT-----------GTCTAAAATGT--GCACTAGT---------TT---------
oryctolagus_cuniculus/1-2852	GTGCACTT----GTA----AACCAAAATAT--GCATTTAT---------TACAATAAG--

snp7(rs407388367)

ovis_aries/1-2852	GATTGGGAGA**C**AGTAGCAGCCATGTGAAAAATT---------------------------
capra_hircus/1-2852	GATTGGGAGATAGTAGCAGCCATGTGAAAAATT---------------------------
bison_bison_bison/1-2852	GATTGGGAGACAGTATCAGCAATGTGAAAAATT---------------------------
bos_grunniens/1-2852	GATTGGGAGACAGTATCAGCAATGTGAAAAATT---------------------------
bos_mutus/1-2852	GATTGGGAGACAGTATCAGCAATGTGAAAAATT---------------------------
bos_taurus/1-2852	GATTGGGAGACAGTATCAGCAATGTGAAAAATT---------------------------
sus_scrofa/1-2852	GATTGGGAGTTTCTACAAGCAATGTGGAAAAAA---------------------------
camelus_dromedarius/1-2852	GATTGTAAGATACTGCAAACAATGTGAAAAAC-ACATGAAATTTGTAACTG---------
canis_lupus_familiaris/1-2852	--
homo_sapiens/1-2852	--
mus_musculus/1-2852	--
oryctolagus_cuniculus/1-2852	--

ovis_aries/1-2852	-----TACATGAAATTTCCTAATTGCATTTGGTTGCCTGAAATATGCATTTATAATAACA
capra_hircus/1-2852	-----TACATGAAATTTCCTAATTGCATTTGGTTGCCTGAAATATGCATTTCAAAAAACA
bison_bison_bison/1-2852	-----TACATCAAATTTCCTAATTGCATTTGGTTGCCTGAAATATGCATTTATAATAACA
bos_grunniens/1-2852	-----TACATCAAATTTCCTAATTGCATTTGGTTGCCTGAAATATGCATTTATAATAACA
bos_mutus/1-2852	-----TACATCAAATTTCCTAATTGCATTTGGTTGCCTGAAATATGCATTTATAATAACA
bos_taurus/1-2852	-----TACATCAAATTTCCTAATTGCATTTGGTTGCCTGAAATATGCATTTATAATAACA
sus_scrofa/1-2852	-------------------------AGGTTGGTTGTCTGAAATAGGCATTTGTAATAACA
camelus_dromedarius/1-2852	-------------------------CAGTTAGTTATCTGAAATATGCATTTATAACAGCA
canis_lupus_familiaris/1-2852	--
homo_sapiens/1-2852	--
mus_musculus/1-2852	--
oryctolagus_cuniculus/1-2852	--

ovis_aries/1-2852	GG--------GTT--------------TT-------------------------------
capra_hircus/1-2852	GG--------GTT--------------TT-------------------------------
bison_bison_bison/1-2852	GG--------TTT--------TTTTTTTT-------------------------------
bos_grunniens/1-2852	GG--------TTT--------TTT--TTT-------------------------------
bos_mutus/1-2852	GG--------TTT--------TTTTTTTT-------------------------------
bos_taurus/1-2852	GG--------TTT--------TTTTTTTT-------------------------------
sus_scrofa/1-2852	GG--------TTT---------------T-------------------------------
camelus_dromedarius/1-2852	GA--------CTT---
canis_lupus_familiaris/1-2852	--
homo_sapiens/1-2852	--
mus_musculus/1-2852	--
oryctolagus_cuniculus/1-2852	--

ovis_aries/1-2852	--
capra_hircus/1-2852	--
bison_bison_bison/1-2852	--
bos_grunniens/1-2852	--
bos_mutus/1-2852	--
bos_taurus/1-2852	--
sus_scrofa/1-2852	--
camelus_dromedarius/1-2852	--
canis_lupus_familiaris/1-2852	--
homo_sapiens/1-2852	--
mus_musculus/1-2852	--
oryctolagus_cuniculus/1-2852	--

```
ovis_aries/1-2852             ------------------------------------------------------------
capra_hircus/1-2852           ------------------------------------------------------------
bison_bison_bison/1-2852      ------------------------------------------------------------
bos_grunniens/1-2852          ------------------------------------------------------------
bos_mutus/1-2852              ------------------------------------------------------------
bos_taurus/1-2852             ------------------------------------------------------------
sus_scrofa/1-2852             ------------------------------------------------------------
camelus_dromedarius/1-2852    ------------------------------------------------------------
canis_lupus_familiaris/1-2852 --GTGCCGGGTGCCAGGCCCGAGGGCTCGGCCGCGGGGGGGGGGCTCAGGTCGCGCTGGT
homo_sapiens/1-2852           ------------------------------------------------------------
mus_musculus/1-2852           ------------------------------------------------------------
oryctolagus_cuniculus/1-2852  ------------------------------------------------------------


ovis_aries/1-2852             ------------------------------------------------------------
capra_hircus/1-2852           ------------------------------------------------------------
bison_bison_bison/1-2852      ------------------------------------------------------------
bos_grunniens/1-2852          ------------------------------------------------------------
bos_mutus/1-2852              ------------------------------------------------------------
bos_taurus/1-2852             ------------------------------------------------------------
sus_scrofa/1-2852             ------------------------------------------------------------
camelus_dromedarius/1-2852    ------------------------------------------------------------
canis_lupus_familiaris/1-2852 CGCCCCGGGCCAGAGGGCTCAGGCCCGGCCACGGCCCGCAGTGACCCCCGGGGCCCACAC
homo_sapiens/1-2852           ------------------------------------------------------------
mus_musculus/1-2852           ------------------------------------------------------------
oryctolagus_cuniculus/1-2852  ------------------------------------------------------------


ovis_aries/1-2852             ------------------TTTC--AC----------------------------------
capra_hircus/1-2852           ------------------TTTC--AC----------------------------------
bison_bison_bison/1-2852      ---------------------C--AT----------------------------------
bos_grunniens/1-2852          ------------------TTTC--AT----------------------------------
bos_mutus/1-2852              ------------------T--C--AT----------------------------------
bos_taurus/1-2852             ------------------TTTC--AT----------------------------------
sus_scrofa/1-2852             ------------------TTTC--AC----------------------------------
camelus_dromedarius/1-2852    ------------------TTTG--AC----------------------------------
canis_lupus_familiaris/1-2852 CTGCTGCCTGGCCCGCGTGCCC--CC----------------------------------
homo_sapiens/1-2852           ------------------TTTC--AC----------------------------------
mus_musculus/1-2852           ------------------CCCC--CT----------------------------------
oryctolagus_cuniculus/1-2852  ------------------CTT---TT----------------------------------


ovis_aries/1-2852             ------------------------------------------------------------
capra_hircus/1-2852           ------------------------------------------------------------
bison_bison_bison/1-2852      ------------------------------------------------------------
bos_grunniens/1-2852          ------------------------------------------------------------
bos_mutus/1-2852              ------------------------------------------------------------
bos_taurus/1-2852             ------------------------------------------------------------
sus_scrofa/1-2852             ------------------------------------------------------------
camelus_dromedarius/1-2852    ------------------------------------------------------------
canis_lupus_familiaris/1-2852 ------------------------------------------------------------
homo_sapiens/1-2852           ------------------------------------------------------------
mus_musculus/1-2852           ------------------------------------------------------------
oryctolagus_cuniculus/1-2852  ------------------------------------------------------------


ovis_aries/1-2852             ------------------------------------------------------------
capra_hircus/1-2852           ------------------------------------------------------------
bison_bison_bison/1-2852      ------------------------------------------------------------
bos_grunniens/1-2852          ------------------------------------------------------------
bos_mutus/1-2852              ------------------------------------------------------------
bos_taurus/1-2852             ------------------------------------------------------------
sus_scrofa/1-2852             ------------------------------------------------------------
camelus_dromedarius/1-2852    ------------------------------------------------------------
canis_lupus_familiaris/1-2852 ------------------------------------------------------------
homo_sapiens/1-2852           ------------------TAATAACAGAAA-------AAA---AAAAAGAAACTTGCTGG
mus_musculus/1-2852           ------------------GACTCACACTAAAGA-------------GAGATCTCTGCTGG
oryctolagus_cuniculus/1-2852  ------------------CACTAGTAATAAAGAAAGG---------AAGGAACTTGCTAG
```

```
ovis_aries/1-2852              ------------------------------------------------------------
capra_hircus/1-2852            ------------------------------------------------------------
bison_bison_bison/1-2852       ------------------------------------------------------------
bos_grunniens/1-2852           ------------------------------------------------------------
bos_mutus/1-2852               ------------------------------------------------------------
bos_taurus/1-2852              ------------------------------------------------------------
sus_scrofa/1-2852              ------------------------------------------------------------
camelus_dromedarius/1-2852     ------------------------------------------------------------
canis_lupus_familiaris/1-2852  ------------------------------------------------------------
homo_sapiens/1-2852            ATGTTTAAGCTTACTTGAGCACTGCTGAAAACCTC------AAGTGAT----------TT
mus_musculus/1-2852            ATGTT------TATTTGAACACTGTTCCACAG----------------TTAGAGTGCTTT
oryctolagus_cuniculus/1-2852   AATTTTAAGCTTCTTTGAGCACTGCTGAACACCTC------CAGTGAT----------TT


ovis_aries/1-2852              ---------------TAATAAAAGAGAAA--GG--AAG----AAATCTCTAGATGTTGAA
capra_hircus/1-2852            ---------------TAATAAAAGAGAAA--GG--AAG----AAATCTGTAGATGTTGAA
bison_bison_bison/1-2852       ---------------TAATAAAAGAGAAA--GG--AAG----AAATCTGTAGAGGTTGAA
bos_grunniens/1-2852           ---------------TAATAAAAGAGAAA--GG--AAG----AAATCTGTAGAGGTTGAA
bos_mutus/1-2852               ---------------TAATAAAAGAGAAA--GG--AAG----AAATCTGTAGAGGTTGAA
bos_taurus/1-2852              ---------------TAATAAAAGAGAAA--GG--AAG----AAATCTGTAGAGGTTGAA
sus_scrofa/1-2852              ---------------TAATGATAAAGAAG--GG--AAGATGTAAATTTGCAGATATTGAG
camelus_dromedarius/1-2852     ---------------TAATAACAGAGAAA--GG--AAG----AAGTTTGTAGATGTTGAA
canis_lupus_familiaris/1-2852  ------------------------------------------------------------
homo_sapiens/1-2852            CTGTTATTTGAAACT---------------------------------------------
mus_musculus/1-2852            CTGTTATTTAAAACT---------------------------------------------
oryctolagus_cuniculus/1-2852   CTGTTATTCCAAACT---------------------------------------------


ovis_aries/1-2852              GCCTATTTGGGCATTTGCTGAACACTTAGAATGACTTCTGTTATTCAAAACTATT-----
capra_hircus/1-2852            GCCTATTTGGGCATTTGCTGAACACTTAGAATGACTTCTGTTATTCAAAACTATT-----
bison_bison_bison/1-2852       GCCTATTTGGGCATTTGCTGAACACTTAGAATGACTTCTGTTATTCAAAACTATT-----
bos_grunniens/1-2852           GCCTATTTGGGCATTTGCTGAACACTTAGAATGACTTCTGTTATTCAAAACTATT-----
bos_mutus/1-2852               GCCTATTTGGGCATTTGCTGAACACTTAGAATGACTTCTGTTATTCAAAACTATT-----
bos_taurus/1-2852              GCCTATCTGGGCATTTGCTGAACACTTAGAATGACTTCTGTTATTCAAAACTATT-----
sus_scrofa/1-2852              CCCCATTGGGGCATTTGCTGCACCCCTAGAATGACTTCTGTTATTCAGAACGATT-----
camelus_dromedarius/1-2852     ACCTATTTGGGCATTTGCTGACAACCTAGAATGACTTCTGTTATTCAAAACTATT-----
canis_lupus_familiaris/1-2852  ------------------------------------------------------------
homo_sapiens/1-2852            ------------------------------------------------------------
mus_musculus/1-2852            ------------------------------------------------------------
oryctolagus_cuniculus/1-2852   ------------------------------------------------------------


ovis_aries/1-2852              ----------------------------------------------------------TC
capra_hircus/1-2852            ----------------------------------------------------------TC
bison_bison_bison/1-2852       ----------------------------------------------------------TC
bos_grunniens/1-2852           ----------------------------------------------------------TC
bos_mutus/1-2852               ----------------------------------------------------------TC
bos_taurus/1-2852              ----------------------------------------------------------TC
sus_scrofa/1-2852              ----------------------------------------------------------TC
camelus_dromedarius/1-2852     ----------------------------------------------------------TC
canis_lupus_familiaris/1-2852  ----------------------------------------------------------AC
homo_sapiens/1-2852            ------------------------------------------------------------
mus_musculus/1-2852            ------------------------------------------------------------
oryctolagus_cuniculus/1-2852   ------------------------------------------------------------


ovis_aries/1-2852              TCATAGGGTTTTTATGT----------------TCTTC----------------------
capra_hircus/1-2852            TCATAGGGTTTTTATGT----------------TCTTC----------------------
bison_bison_bison/1-2852       TCATAGGGTTTTTATGT----------------TCTTC----------------------
bos_grunniens/1-2852           TCATAGGGTTTTTATGT----------------TCTTC----------------------
bos_mutus/1-2852               TCATAGGGTTTTTATGT----------------TCTTC----------------------
bos_taurus/1-2852              TCATAGGGTTTTTATGG----------------TCTTC----------------------
sus_scrofa/1-2852              TCACAGTGTTTCTATGT----------------TCTTC----------------------
camelus_dromedarius/1-2852     TCACAGTGTTTTTATGT----------------TCTTC----------------------
canis_lupus_familiaris/1-2852  C--------------------------------CCCAC----------------------
homo_sapiens/1-2852            --------------------------------------CTCT----CAGTAATTTTTTTG
mus_musculus/1-2852            ------------------------------------------------------------
oryctolagus_cuniculus/1-2852   ------------------------------------------------------------
```

```
ovis_aries/1-2852             ---------------------------------------------------------ATA
capra_hircus/1-2852           ---------------------------------------------------------ATA
bison_bison_bison/1-2852      ---------------------------------------------------------ACA
bos_grunniens/1-2852          ---------------------------------------------------------ACA
bos_mutus/1-2852              ---------------------------------------------------------ACA
bos_taurus/1-2852             ---------------------------------------------------------ACA
sus_scrofa/1-2852             ---------------------------------------------------------ACA
camelus_dromedarius/1-2852    ---------------------------------------------------------ACA
canis_lupus_familiaris/1-2852 ---------------------------------------------------------ACG
homo_sapiens/1-2852           TTCTTC---------------------------------------------------ACA
mus_musculus/1-2852           ------------------------------GTCAGTCTCAGAGCTTCTATGCTCTTGACA
oryctolagus_cuniculus/1-2852  ------CCTTCACAGTGTTTTGTGTGCTTC---------------------------ACA
                                                                                       *

                                                                                snp8(rs408710650)
ovis_aries/1-2852             G------AG--------------T----ATCTGATTTTGAAAGCTATTAC---AGTGGAA
capra_hircus/1-2852           G------AG--------------T--------AATTTTGAAAGCTATTAC---AGTGGAA
bison_bison_bison/1-2852      G------AG--------------TATCT----AATTTTGAAAGCTATTAG---AGTGGAA
bos_grunniens/1-2852          G------AG--------------TATCT----AATTTTGAAAGCTATTAG---AGTGGAA
bos_mutus/1-2852              G------AG--------------TATCT----AATTTTGAAAGCTATTAG---AGTGGAA
bos_taurus/1-2852             G------AG--------------TATCT----AATTTTGAAAGCTATTAG---AGTGGAA
sus_scrofa/1-2852             A------ATT---AAAA------TGTCT----AATTTTGAAAGCTATTAC---ACTGGAA
camelus_dromedarius/1-2852    A------ATT---ACAA------TATCT----AATTTTGAAAGCTATTAC---ACTGGAA
canis_lupus_familiaris/1-2852 G------------AGG-------GGGGT----AATTCTGAGACACACCAC---ACTGGAG
homo_sapiens/1-2852           G---TTA--------AA------AATTT----AATTTTGAGAGCTATTAC---ACTGAAG
mus_musculus/1-2852           A---GCT------AGAA------TAT-T----AATTGTGAAAGCTACTGC---ACTGGAA
oryctolagus_cuniculus/1-2852  G---ATT------AAAA------TGTTT----CCTTTCGGAGGCTATTAC---ACTGAGG
                                                                **  *      *       * **


ovis_aries/1-2852             AGGATAAA-----------------------AAAATGTTCTTAA-------------TAA
capra_hircus/1-2852           AGGATAAA-----------------------AAAATGTTCTTAA-------------TAA
bison_bison_bison/1-2852      AGGATAAA-----------------------AGAATATTCTTAA-------------TAA
bos_grunniens/1-2852          AGGATAAA-----------------------AGAATATTCTTAA-------------TAA
bos_mutus/1-2852              AGGATAAA-----------------------AGAATATTCTTAA-------------TAA
bos_taurus/1-2852             AGGATAAA-----------------------AGAATATTCTTAA-------------TAA
sus_scrofa/1-2852             AGTATAA------------------------AAAATATTTTTAA-------------AAA
camelus_dromedarius/1-2852    AGTATAAA-------------------------AATATTTTTAA-------------AAA
canis_lupus_familiaris/1-2852 AGCAACAA---------------CAACCAAA--AGGGTTTTT-A-------------AAA
homo_sapiens/1-2852           AATATGCA------------AAA-----------------------------ATTAAATA
mus_musculus/1-2852           AGTTAAAG------------A-A---------------------G---TTAA-----ATA
oryctolagus_cuniculus/1-2852  AGCAAGAA------------A-A---------------------TATTTTAA-----ATA
                              *                                                          *

                                                   snp9(GAGCAATAAGG)
ovis_aries/1-2852             ACTTAATGTATTAGTAA----GAGCAATAAGGAAGTAAACACAGCATAAT---GATA---
capra_hircus/1-2852           ACTTAATGTATTAGTAA----GAGCAATAAGGAAGTAAACACAGCATAAT---GATA---
bison_bison_bison/1-2852      ACTTAATGTATTAGTAA----GAGCAATAAGGAAGTAAACACAGCATAAT---GAAA---
bos_grunniens/1-2852          ACTTAATGTATTAGTAA----GAGCAATAAGGAAGTAAACACAGCATAAT---GAAA---
bos_mutus/1-2852              ACTTAATGTATTAGTAA----GAGCAATAAGGAAGTAAACACAGCATAAT---GAAA---
bos_taurus/1-2852             ACTTAATGTATTAGTAA----GAGCAATAAGGAAGTAAACACAGCATAGT---GAAA---
sus_scrofa/1-2852             ATTTAATGTTTTGGTAA----GAGCAATGATGAAGTAAACATAGCATAATGGT-------
camelus_dromedarius/1-2852    ATTTAATGTATTAGTAA----GAGCAATGATGAAGTAAACATAGCATAATAGA-------
canis_lupus_familiaris/1-2852 ATGTACCGTATCGGGAA----GACCAGTGAGGAAGTGCAGGCGGTGCAATGAC-------
homo_sapiens/1-2852           TCTTAATGTGTTA----GTAAGAACAATAATGAAGTAAACATAGCATAATAAT-------
mus_musculus/1-2852           T-TAAATGCATTATCAT----GAGCCACGATGAAGCAGGC--------------------
oryctolagus_cuniculus/1-2852  C-TTTATGTATTAGTAA----GAGCAATGACGAAATAAAC--------------------
                                     *  *          ** *    * ***


ovis_aries/1-2852             AAT-----------------CATGAGCCAATGAGCAGAAAATGCT--AAGAAATAAACAT
capra_hircus/1-2852           AAT-----------------CATGAGCCAATGAGCAGAAAATGCT--AAGAAATAAACAT
bison_bison_bison/1-2852      AAT-----------------CATGAGCTAATCAGCAGAAAATTCT--AAGAAATAAACAT
bos_grunniens/1-2852          AAT-----------------CATGAGCTAATCAGCAGAAAATTCT--AAGAAATAAACAT
bos_mutus/1-2852              AAT-----------------CATGAGCTAATCAGCAGAAAATTCT--AAGAAATAAACAT
bos_taurus/1-2852             AAT-----------------CATGAGCTAATCAGCAGAAAATTCT--AAGAAATAAACAT
sus_scrofa/1-2852             AAT-----------------TATGAGCTAATTATCAGAAAATGCC--AAGAAATAAACAT
camelus_dromedarius/1-2852    AAT-----------------CATGAGCTAATGATTAGAAAATGCC--AAGAAATAAACAT
canis_lupus_familiaris/1-2852 AGC-----------------CGCAAGCCGAGTGTCACGAGGTGCC--ACGGAGCGAACAC
homo_sapiens/1-2852           CAT-----------------CATGAACTAAATATTAGAAAATGCCT-AAGAAATAAACAT
mus_musculus/1-2852           ---ATAGCAA-AATAATACTCAGGA-CCGAGTATCAGAAAACACTC-AGGAAATGAGCAT
oryctolagus_cuniculus/1-2852  --------------------CGTGACTTCAAATTCAGAAAATGTCT-AGGAAATAAACAC
                                                      *    *     *  *        * * *   * **
```

```
ovis_aries/1-2852             T----------------------------TTAATTGAGTAGG-------------TTATG
capra_hircus/1-2852           T----------------------------TTAATTGAGTAGG-------------TTATG
bison_bison_bison/1-2852      T----------------------------TTAAT--------------------------
bos_grunniens/1-2852          T----------------------------TTAATTGAGTAGG-------------TTATG
bos_mutus/1-2852              T----------------------------TTAAT--------------------------
bos_taurus/1-2852             T----------------------------TTAAT--------------------------
sus_scrofa/1-2852             T----------------------------TTA-TCAAGTAGG-------------TTATG
camelus_dromedarius/1-2852    T----------------------------TTAATCAAATAGG-------------TTATG
canis_lupus_familiaris/1-2852 G------------------------------------------------------CCACG
homo_sapiens/1-2852           T-------------TT--AATTGGGTAGG--------------------------TTATG
mus_musculus/1-2852           T-----------------AACTGGG-AGG--------------------------TCATG
oryctolagus_cuniculus/1-2852  T-------------TC--AGTTGGGCAAG--------------------------TTACG


ovis_aries/1-2852             GC----------TTACAAAGTCCCACT-TATACCCTGAC------CA--TGGTAC-----
capra_hircus/1-2852           GC----------TTACAAAGTCCCACT-TATACCCTGAC------CA--TGGTAC-----
bison_bison_bison/1-2852      -------------TACAAAGTTCCACT-TATACCCTGAC------CA--TGGTAC-----
bos_grunniens/1-2852          GC----------TTACAAAGTTCCACT-TATACCCTGAC------CA--TGGTAC-----
bos_mutus/1-2852              -------------TACAAAGTTCCACT-TATACCCTGAC------CA--TGGTAC-----
bos_taurus/1-2852             -------------TACAAAGTTCCACT-TATACCCTGAC------CA--TGGTAC-----
sus_scrofa/1-2852             GC----------TCACAAAGTCCTGCT-TATACCTTGAC------CA--TGGTAC-----
camelus_dromedarius/1-2852    GC----------TCACAAAGTCCTACT-TATACCTTGGC------CA--TGGTGC-----
canis_lupus_familiaris/1-2852 GCAGCCGGGCCGCCACAA-GGCCCACC-TGCACCTGGGG----------TGGCGC-----
homo_sapiens/1-2852           GC----------TCACAAAGTCCTCCT-TGTACCTTGAC--C--A----TAGTAC-----
mus_musculus/1-2852           GT------------GTGAAATGCTCCTGTGCC---CCTT--C--A----TAGCAC-----
oryctolagus_cuniculus/1-2852  GC----------TCCTGAAGTCC-TCT-CGTACCTTGAC--C--A----TGGTGC-----
                                               *    *  *                       * *  *
```
