# Supplementary material for: Identification and bioinformatic functional analysis of novel and known polymorphisms in the myostatin gene of Ukrainian Carpathian Mountain sheep
Source: Sci Rep. 2026 Mar 23;16:14628. doi: 10.1038/s41598-026-44326-6 (PMC13153238; doi:10.1038/s41598-026-44326-6)
Supplement: Supplementary file 4 — Supplementary Material 4 [file 41598_2026_44326_MOESM4_ESM.pdf]

SNV	Folding region	Allele	Secondary structure
c.373+241T>C	41-441	T	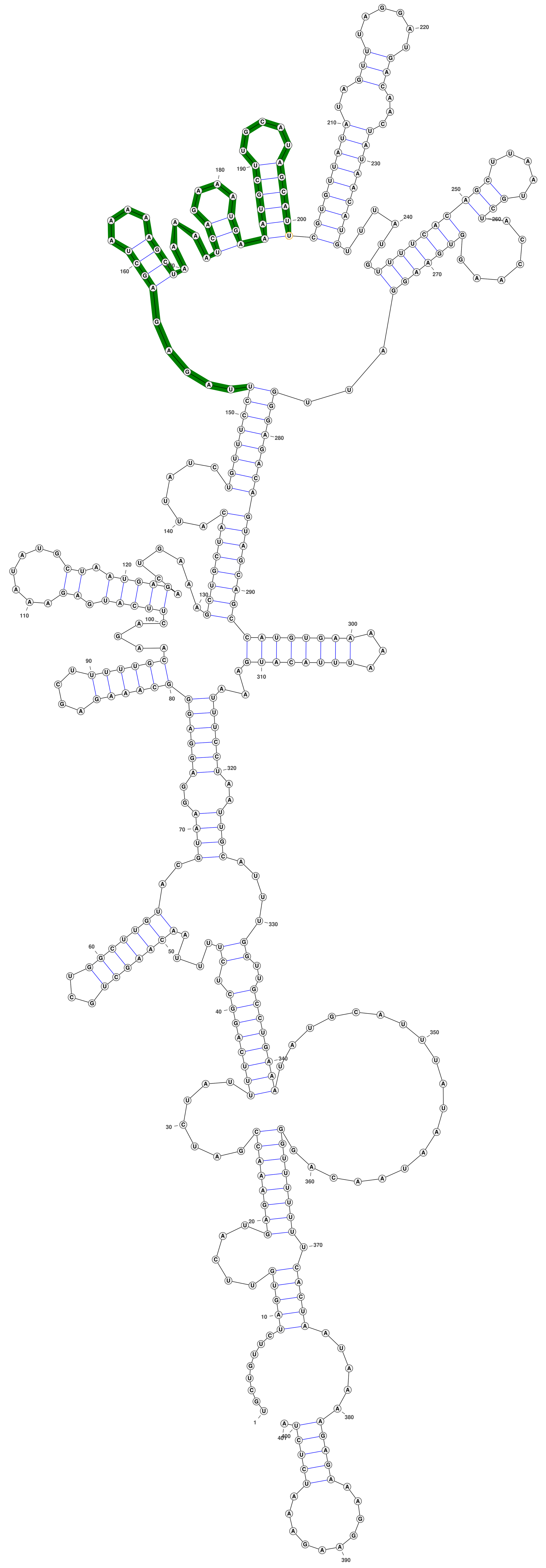

c.373+241T>C	41-441	C	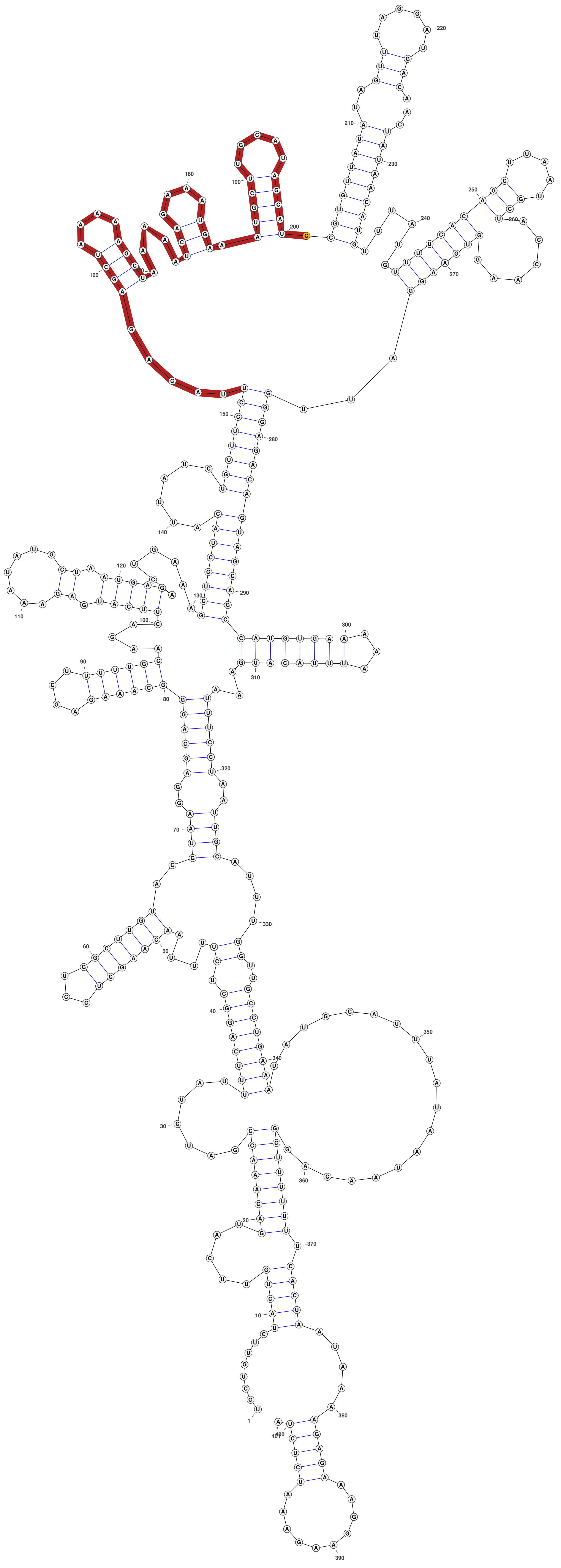

c.373+243G>A	43-443	G	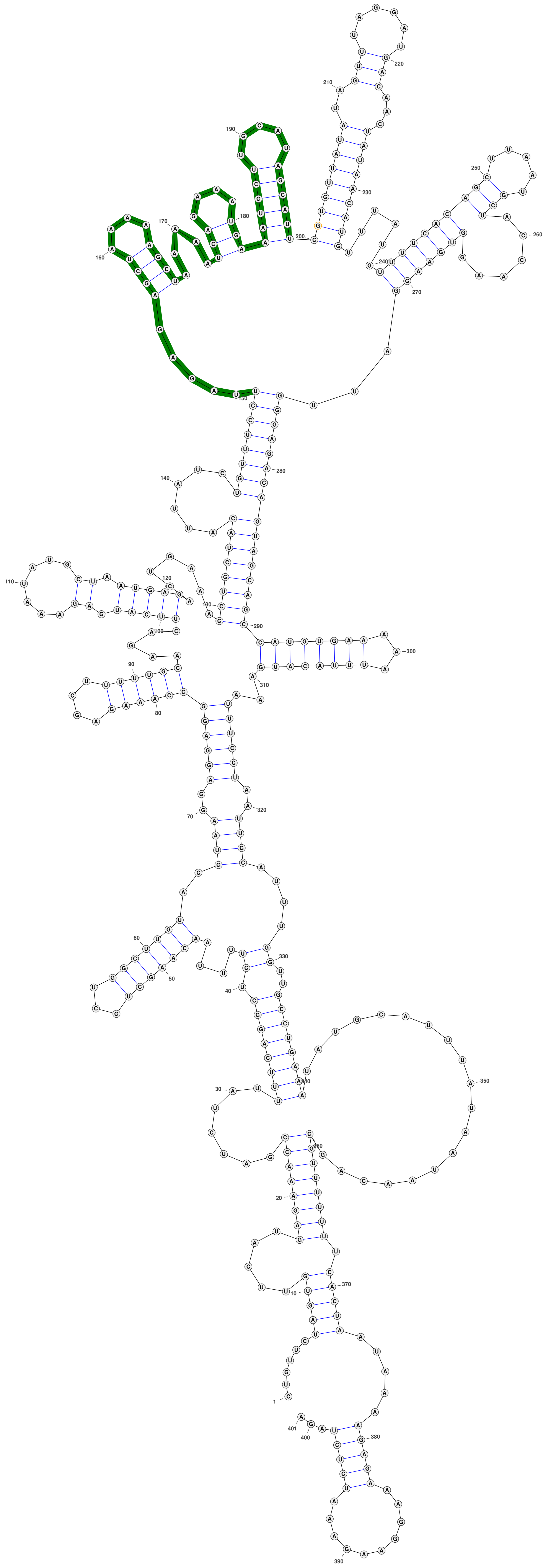

c.373+243G>A	43-443	A	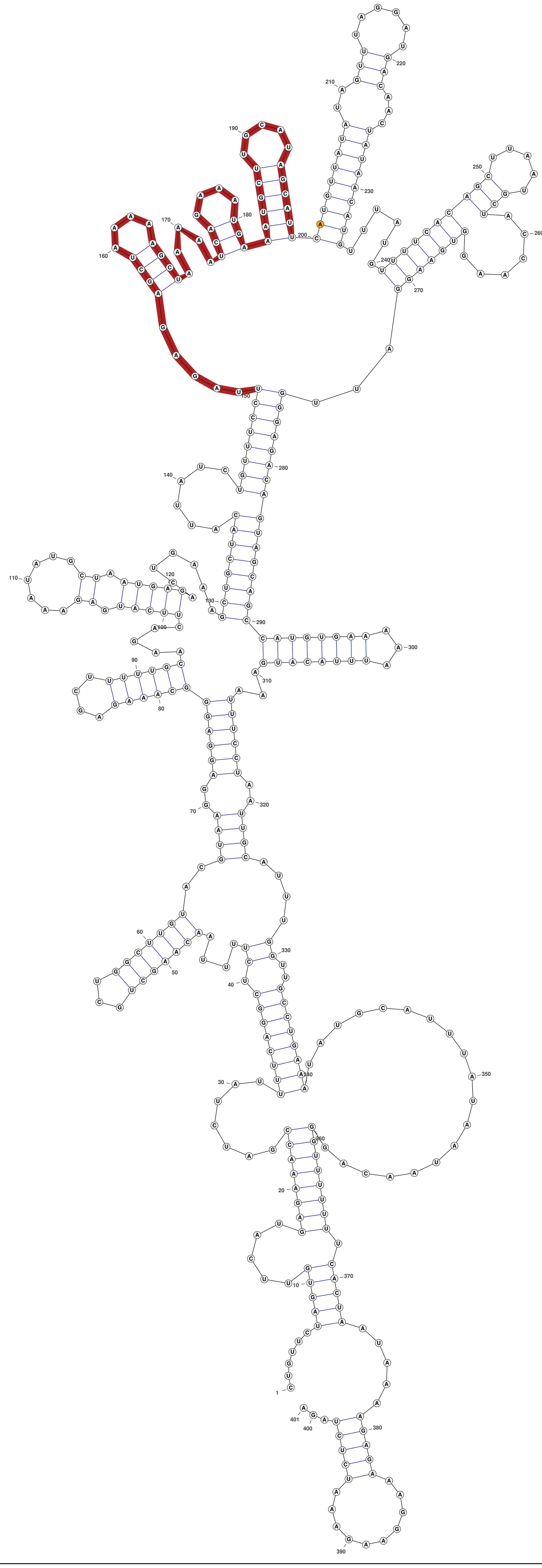

c.373+246T>C	46-446	T	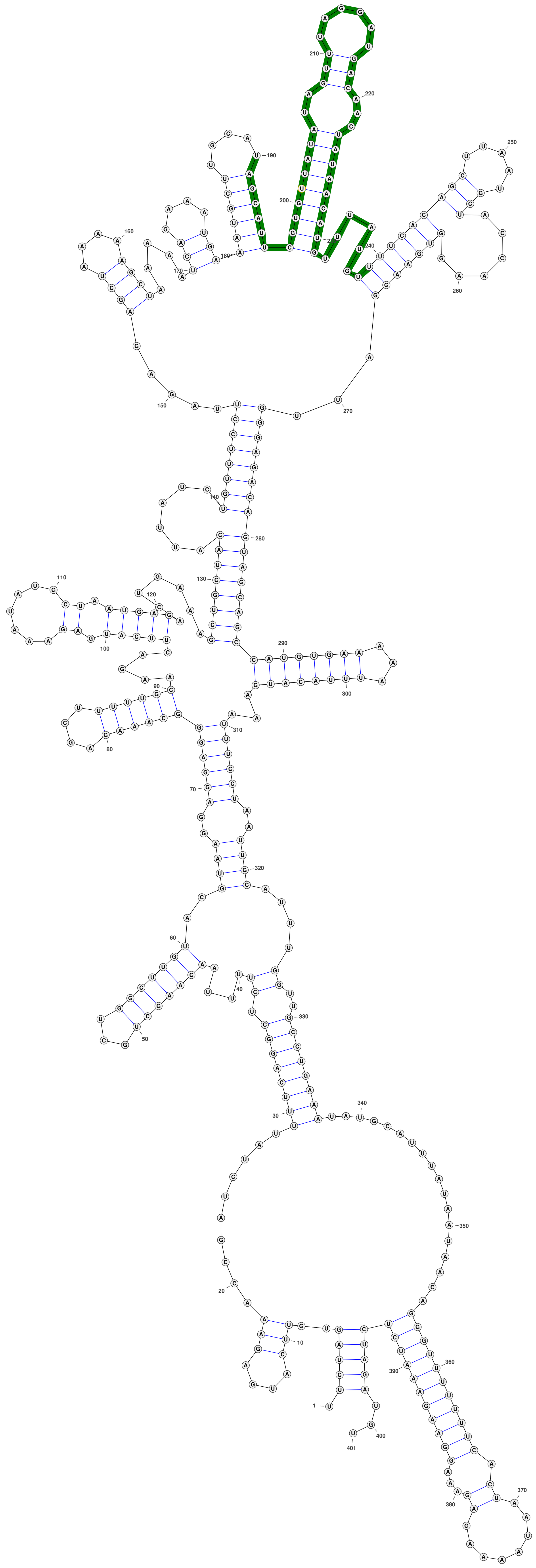

c.373+246T>C	46-446	C	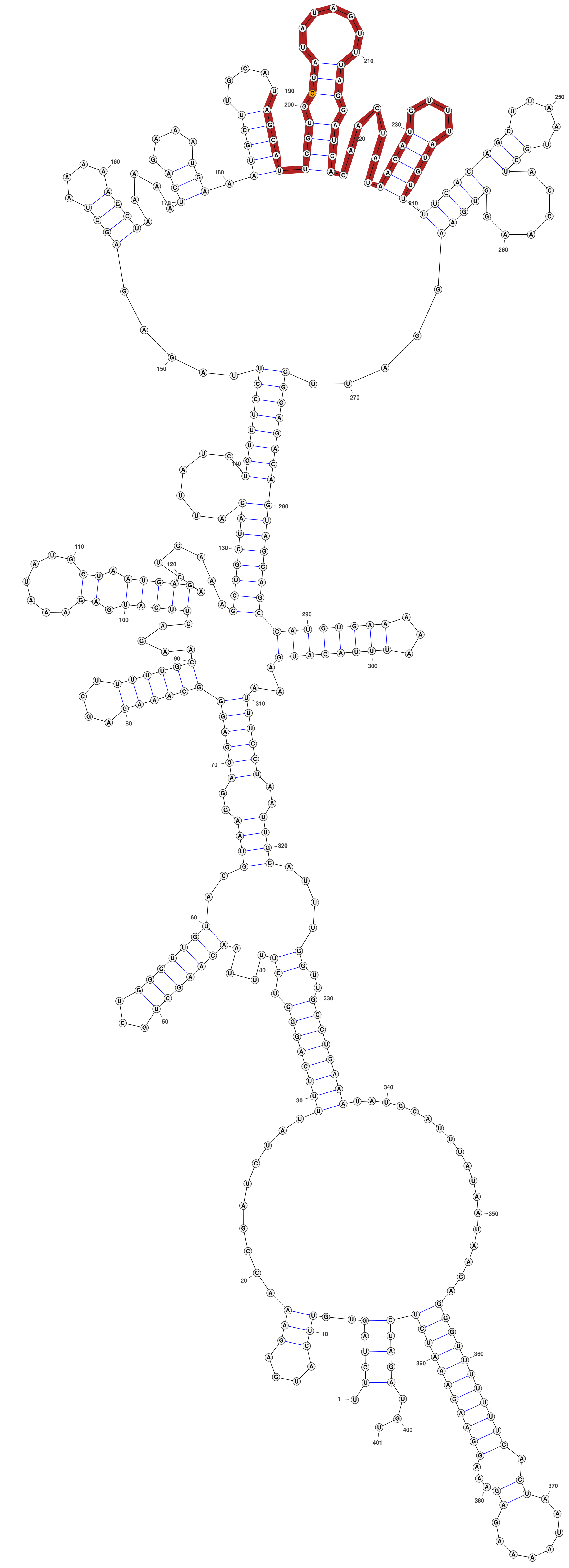

c.373+249T>C	49-449	T	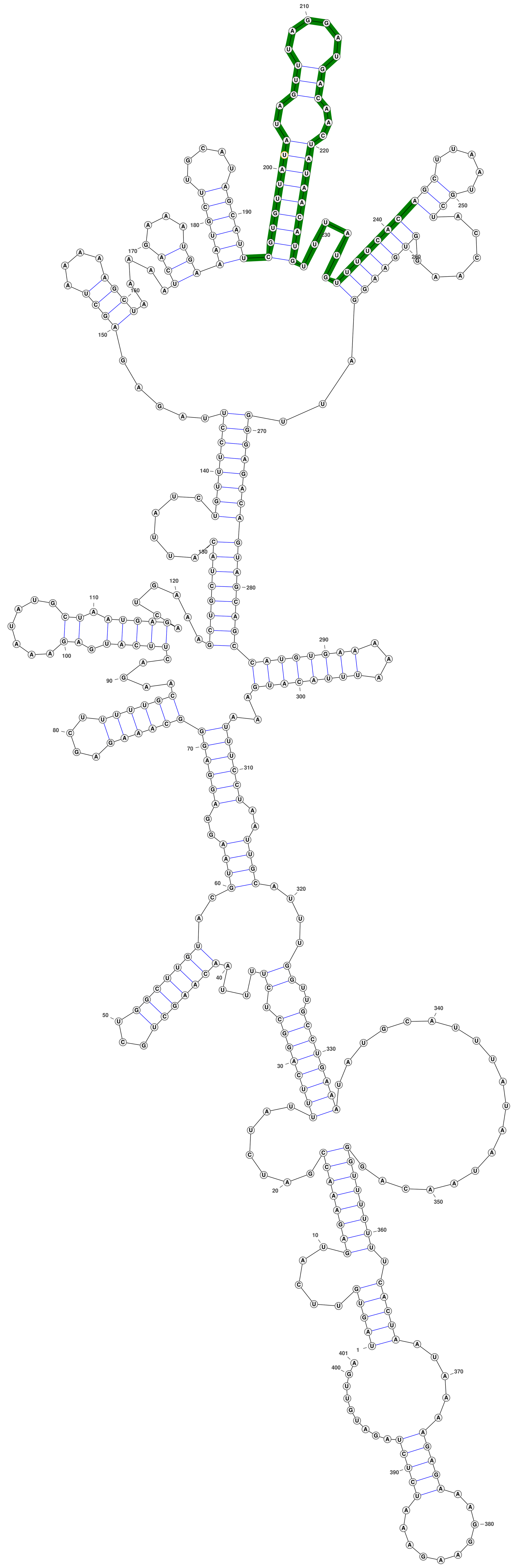

c.373+249T>C	49-449	C	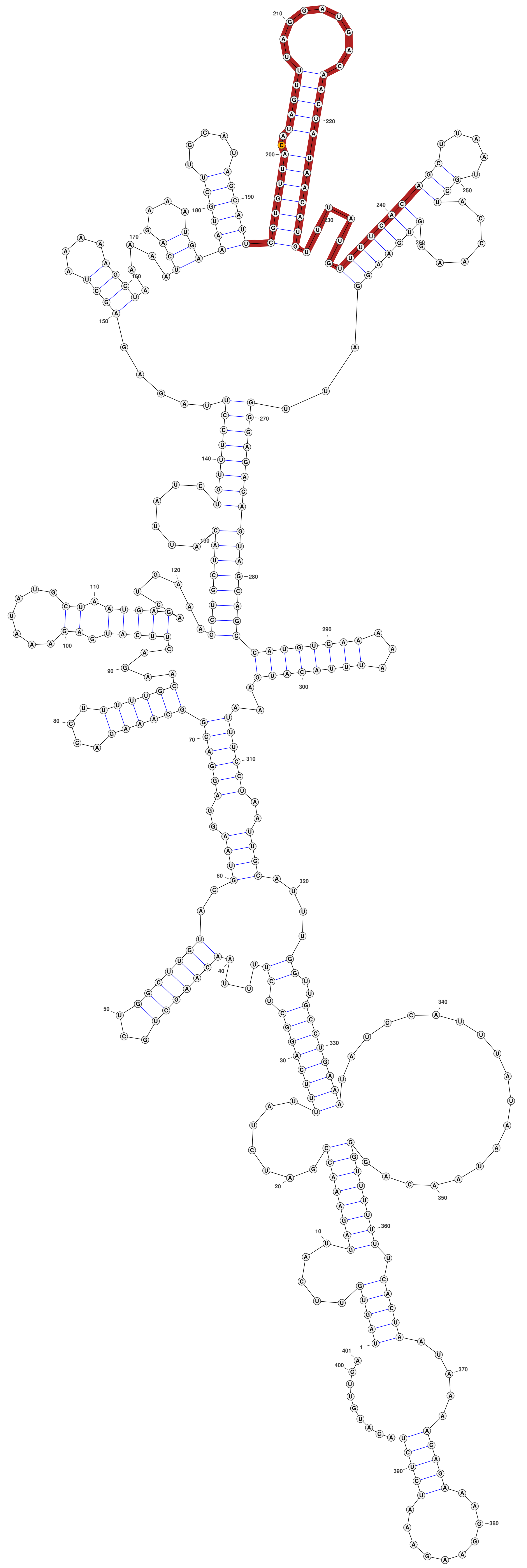

c.373+259G>T	59-459	G	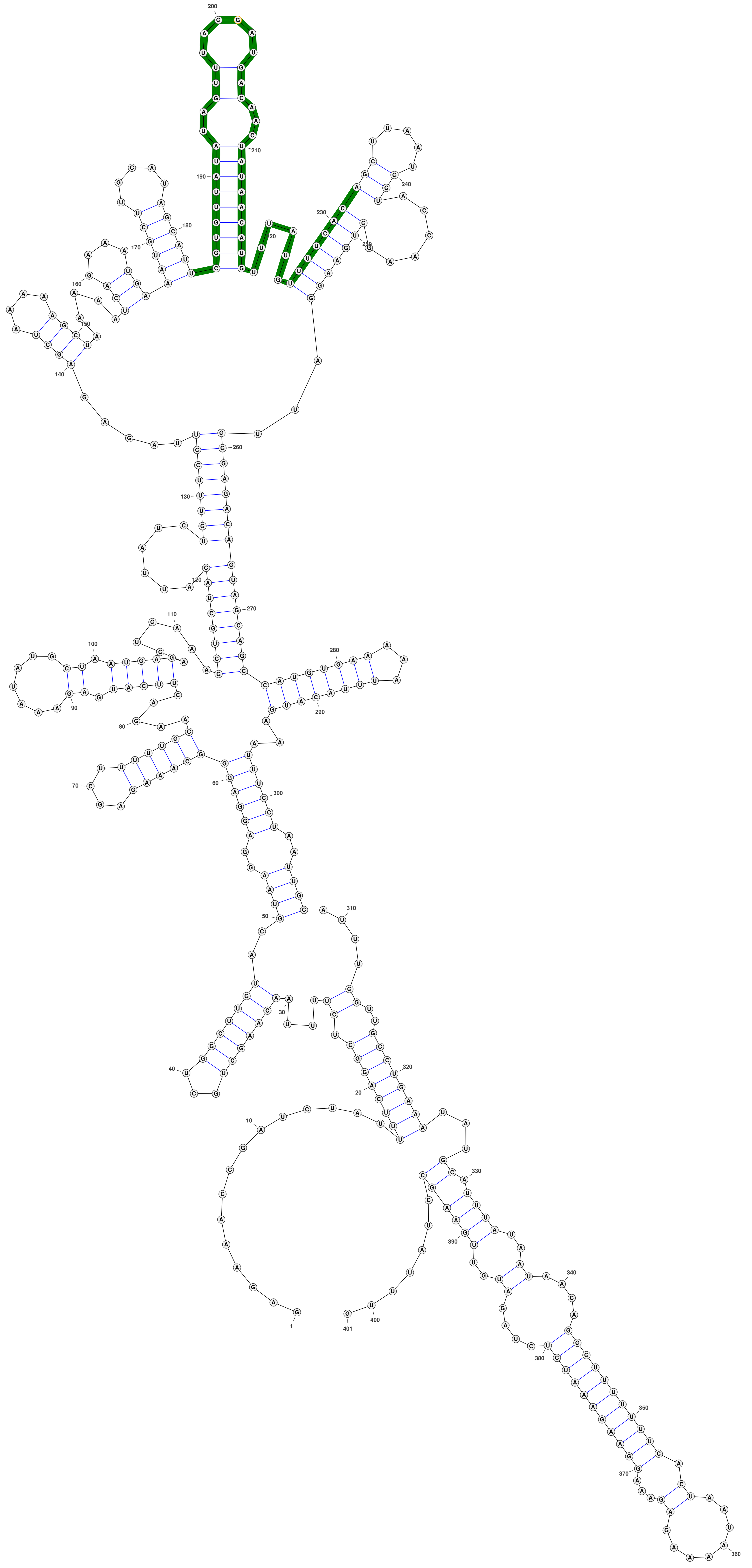

c.373+259G>T	59-459	T	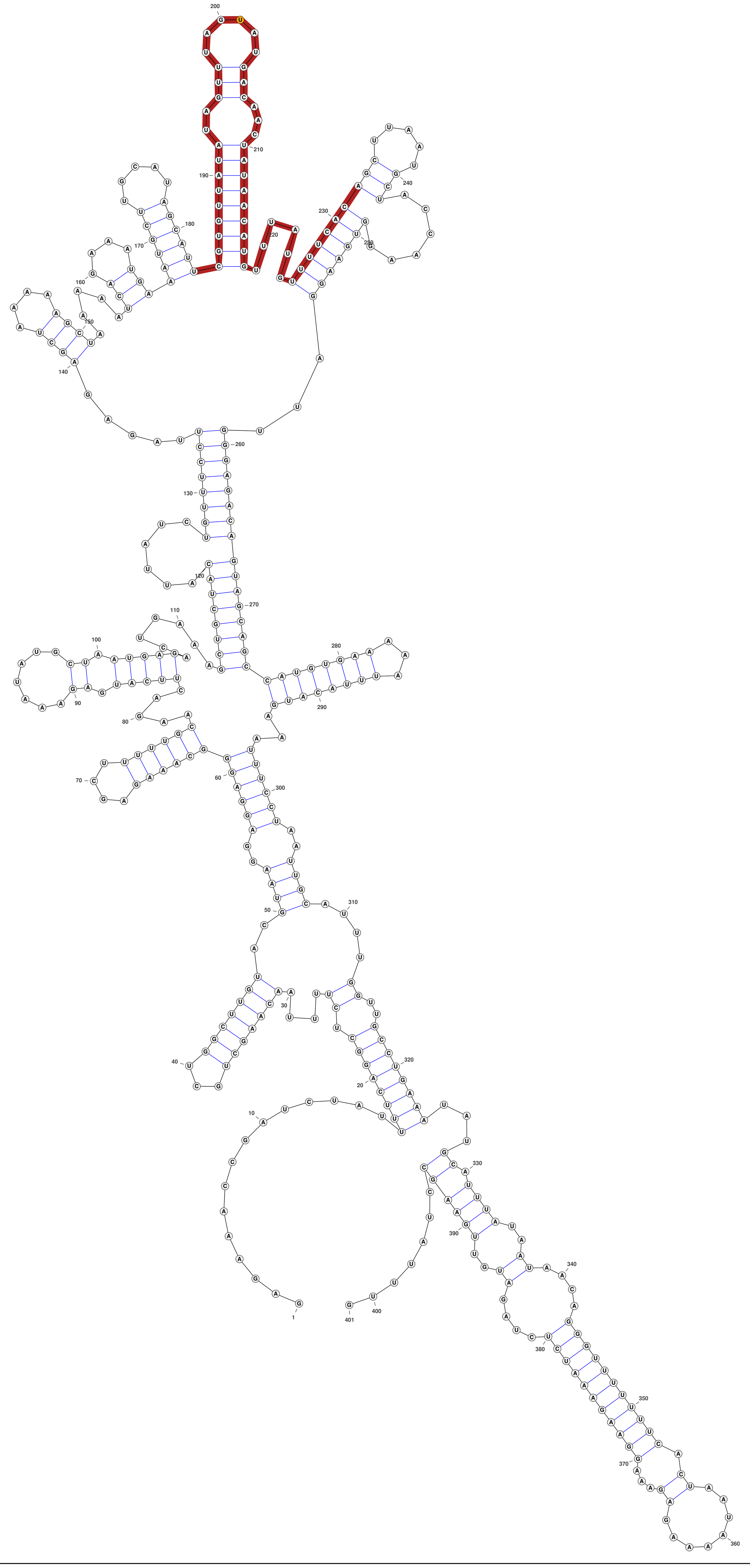

c.373+283T>C	83-483	T	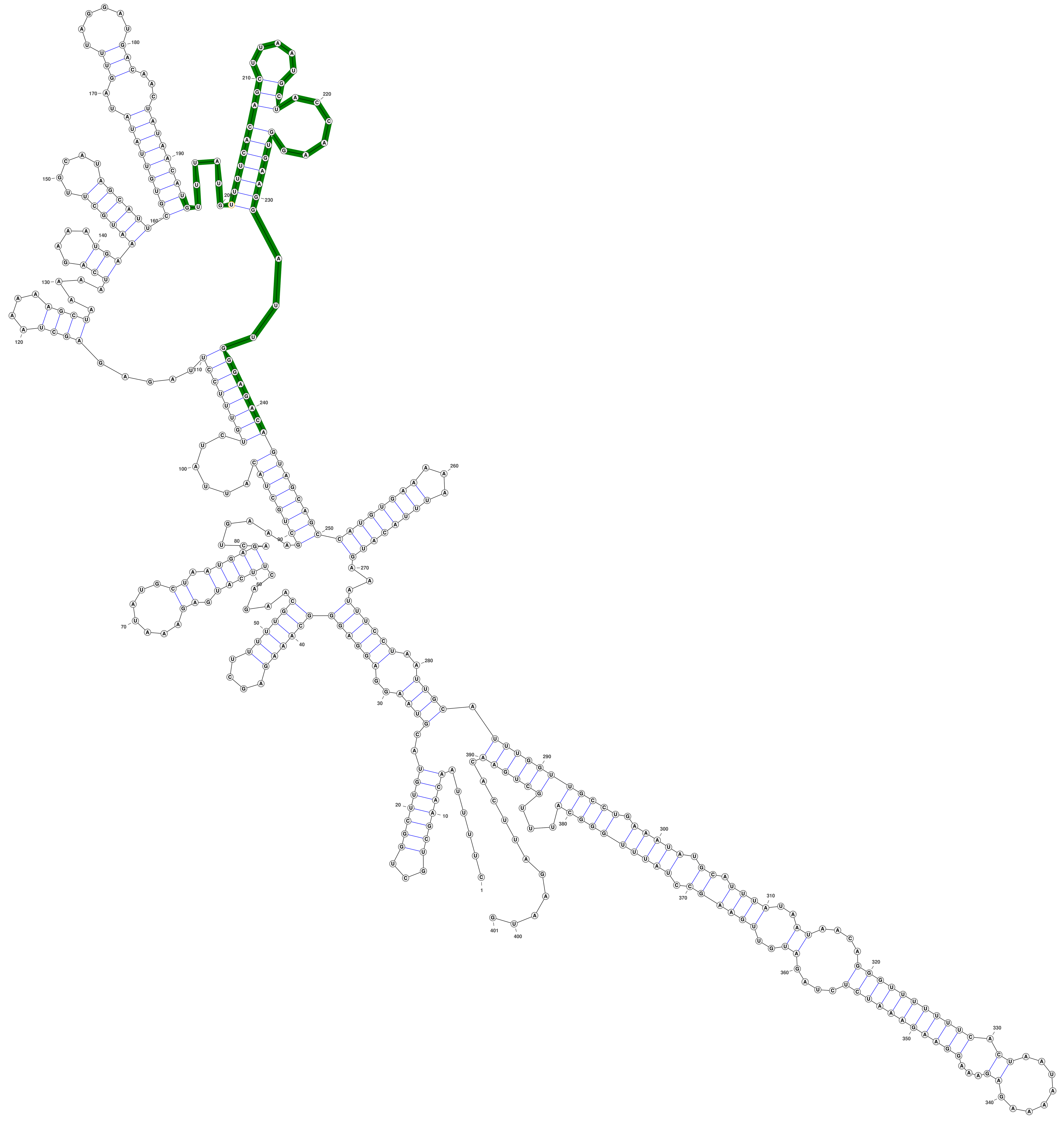

c.373+283T>C	83-483	C	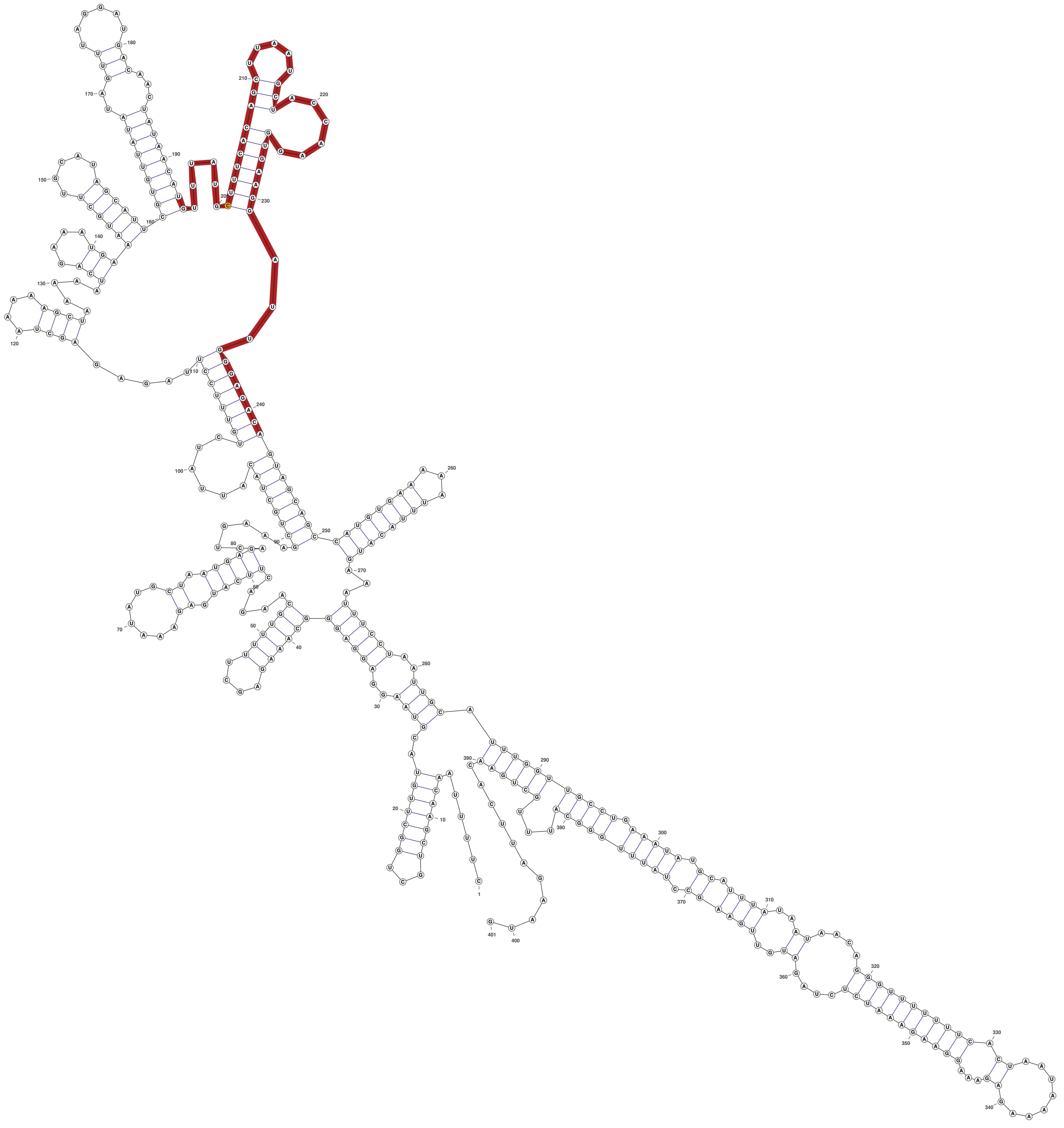

c.373+323C>T	123-523	C	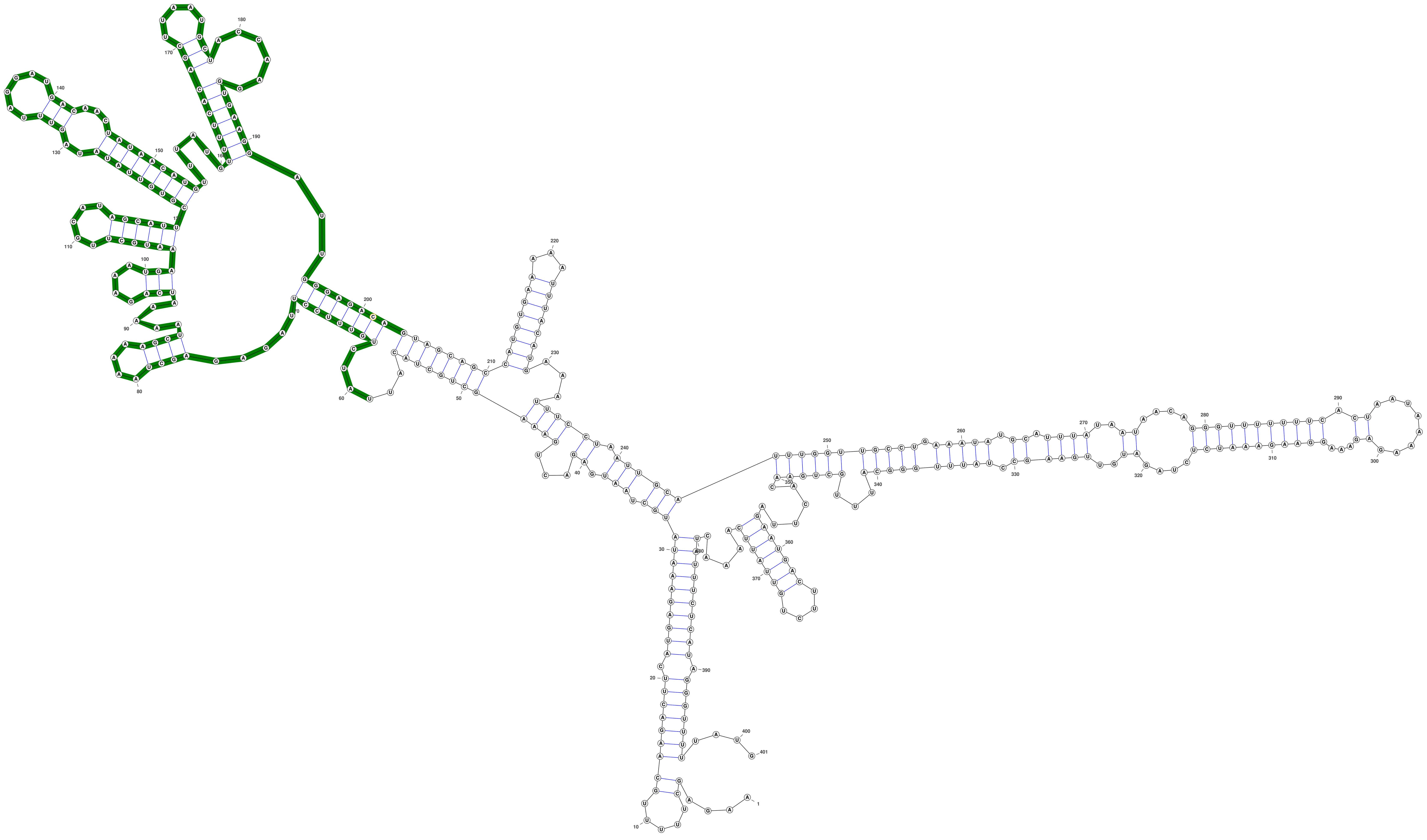
c.373+323C>T	123-523	T	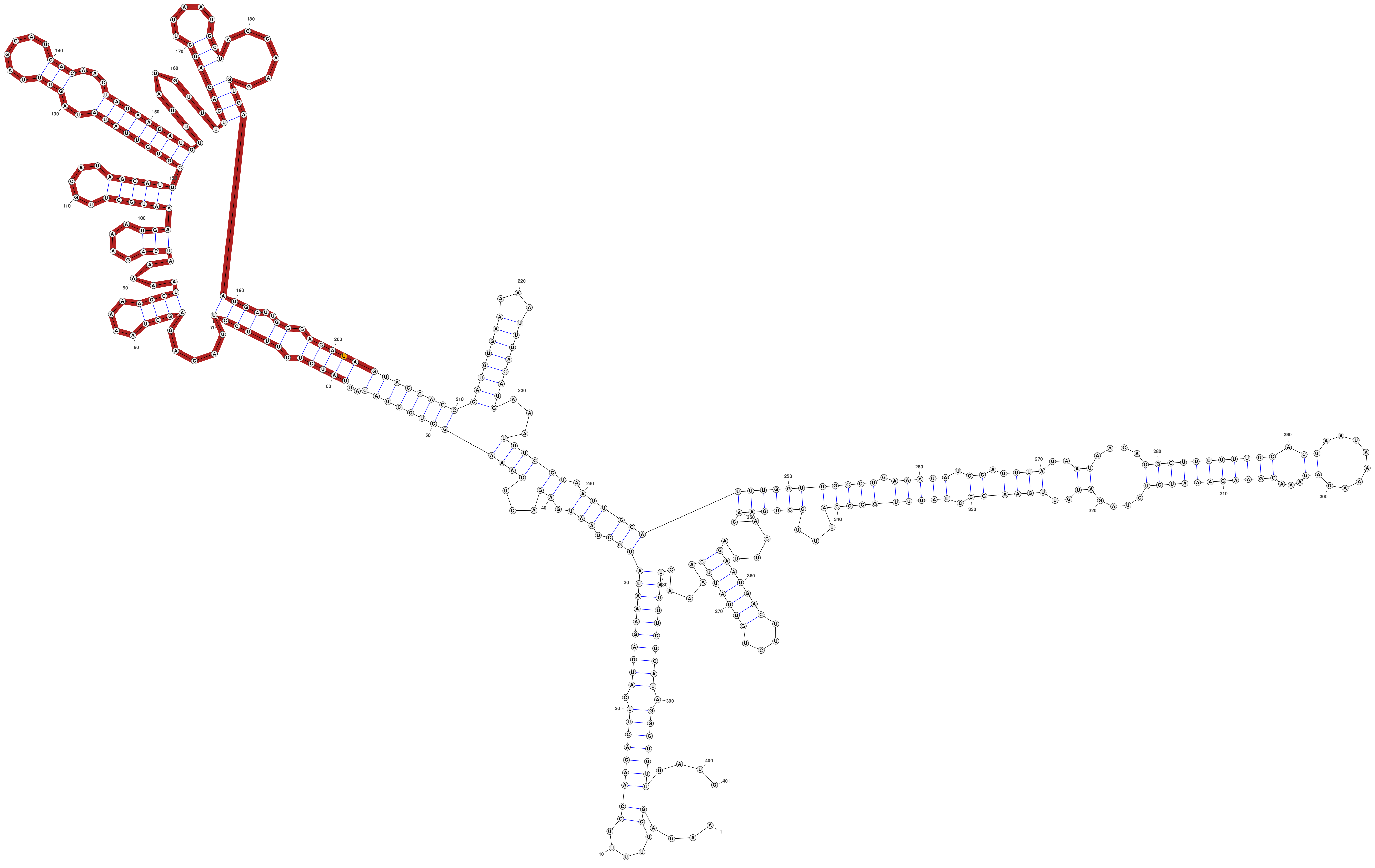

c.373+563G>A	363-763	G	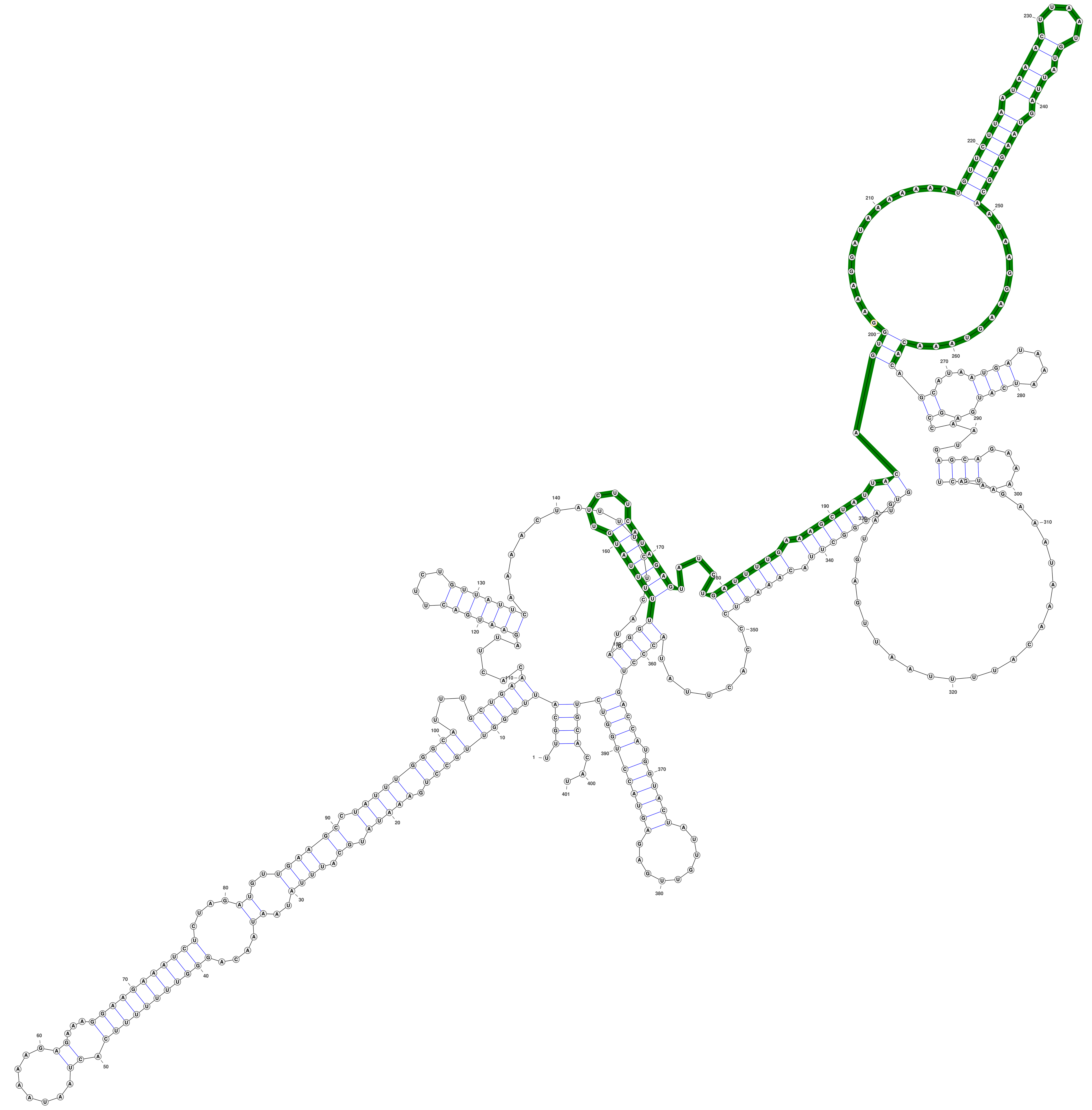

c.373+563G>A	363-763	A	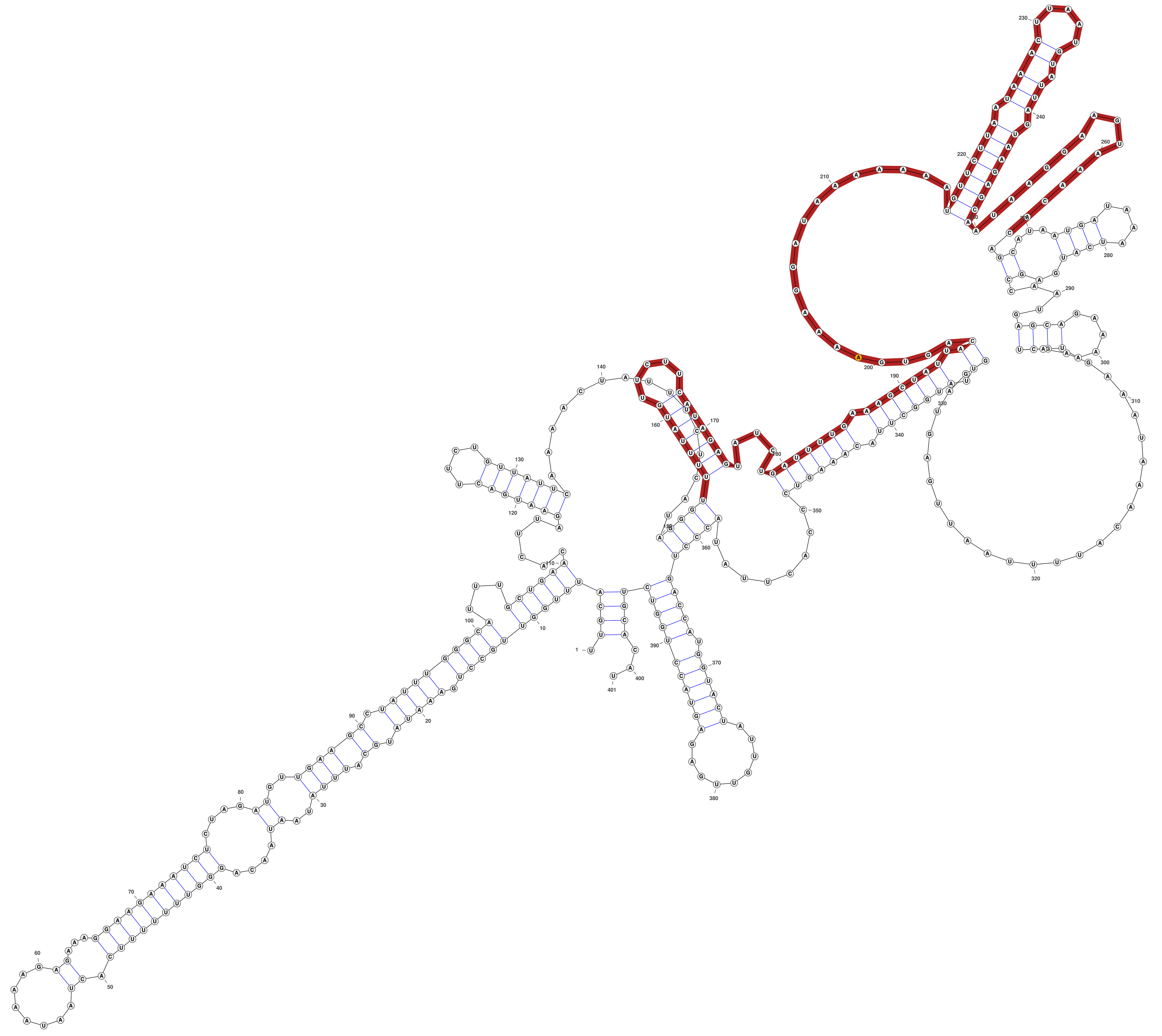

c.373+607G>A	407-807	G	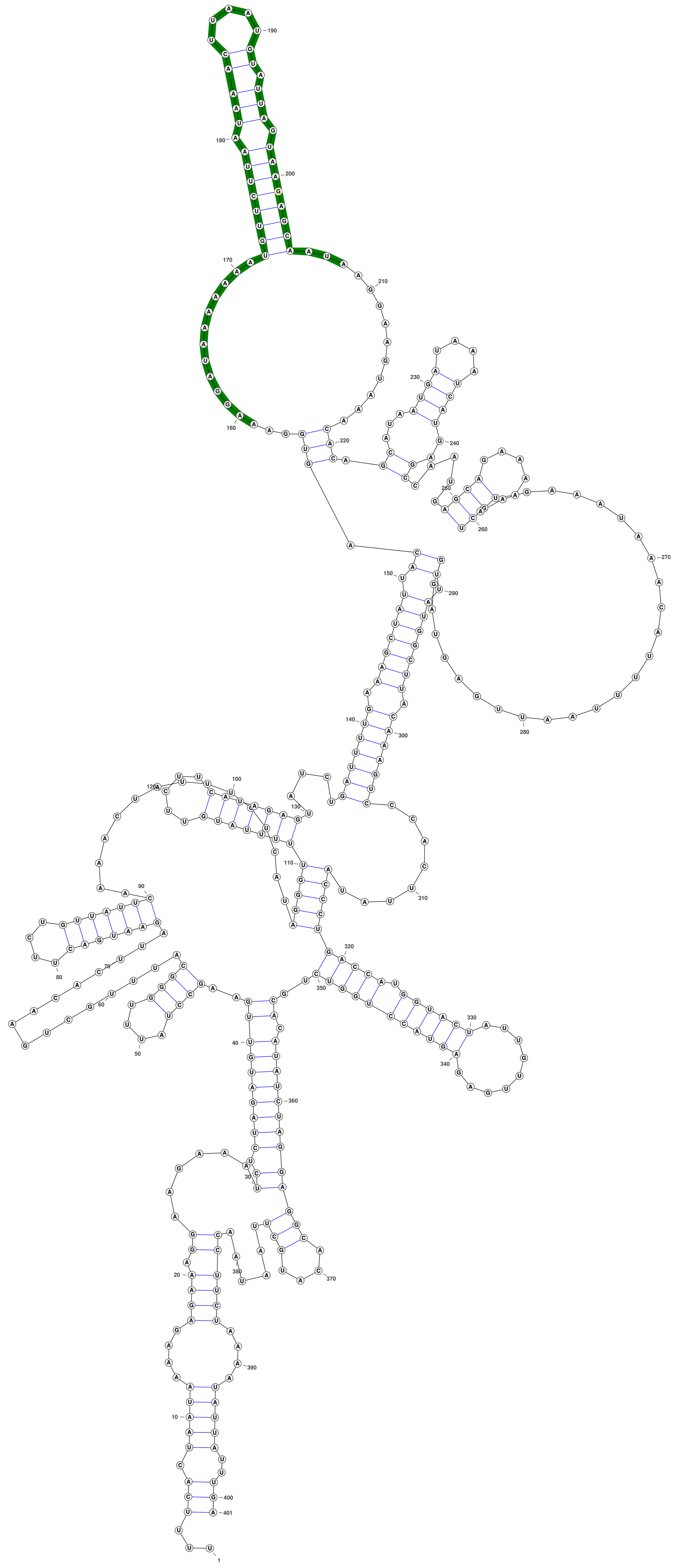

c.373+607G>A	407-807	A	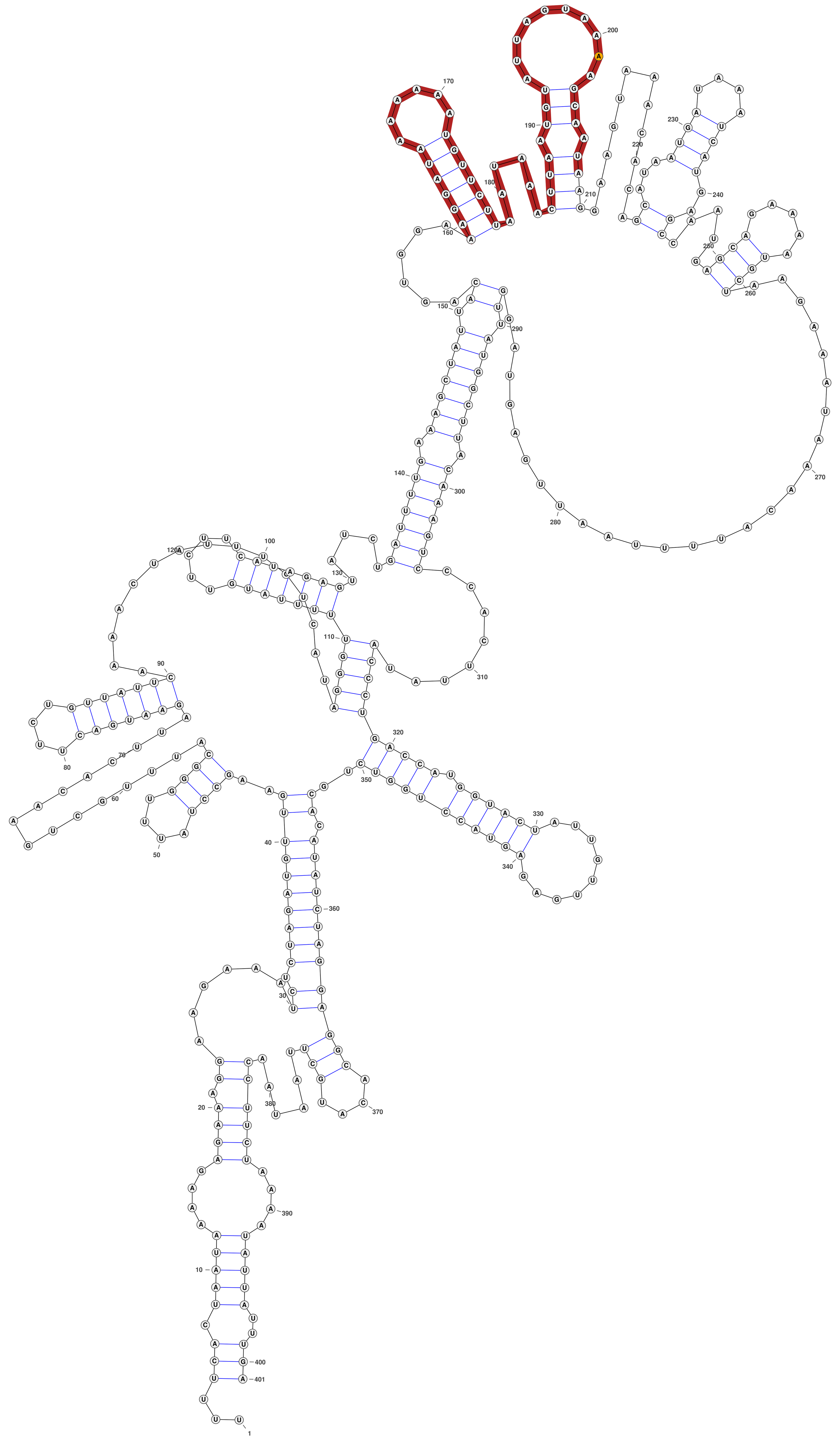
