# Supplementary material for: Identification and bioinformatic functional analysis of novel and known polymorphisms in the myostatin gene of Ukrainian Carpathian Mountain sheep
Source: Sci Rep. 2026 Mar 23;16:14628. doi: 10.1038/s41598-026-44326-6 (PMC13153238; doi:10.1038/s41598-026-44326-6)

Predicted miRNAs in intron 1 of sheep *MSTN* gene

Start Position : 578
End Position : 658
Sequence Size : 81 nucleotides
Minimum Free Energy : -12.6 kcal/mol
Hairpin
(((((....((..((((.(.(((((.((...((..(............)..)).)).))))).)...))))..)).)))))
Sequence
UGUUCUUAAUAAACUUAAUGUAUUAGUAAGAGCAAUAAGGAAGUAAACACAGCAUAAUGAUAAAUCAUGAGCCAAUGAGCA

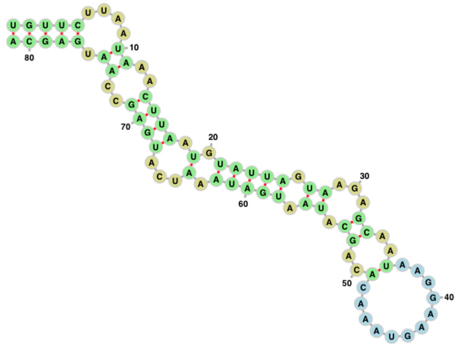

Start Position : 1357
End Position : 1429
Sequence Size : 73 nucleotides
Minimum Free Energy : -27 kcal/mol
Hairpin
((((((.(..((((...........(((((((((.......)))))))))..)))).........).))))))
Sequence
UCAGUUAGUUGCUCACUGUGUCUUGUCCCCAGGUAAUUCAGGCCUGGGGGAAGGGUUCCUUCCUCCAGACUGA

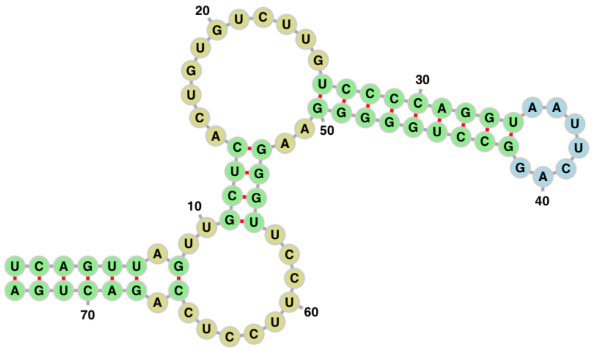

Start Position : 1359
End Position : 1457
Sequence Size : 99 nucleotides
Minimum Free Energy : -35.1 kcal/mol
Hairpin
(((((.(((((..((((.........((((......(((..
(((((((((((...))))))))))).))).)))).)))).......)))))...)))))
Sequence
AGUUAGUUGCUCACUGUGUCUUGUCCCCAGGUAAUUCAGGCCUGGGGAAGGGUUCCUUCCUCCAGACUGAUUGGUACAGCUGCU
CAGUAAGUGUAACU

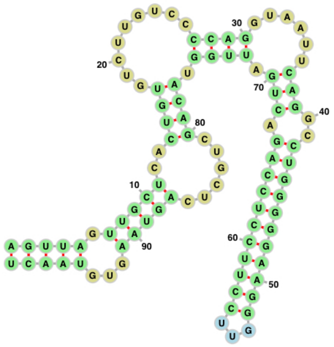

Start Position : 1593
End Position : 1707
Sequence Size : 115 nucleotides
Minimum Free Energy : -26 kcal/mol
Hairpin
((((((.(((...((((.((((((.(((..((((..((..((..((.
((.....)).)).)).))..)))).........))).))))))....))))...)))....))))))
Sequence
GGAAGUAGGCUGCUCAUAAACAGCUGAAAAAACAUAUACCUAAAAGAUUUUGACAAGCUGUAAUAAUUGUUAUACUUGAUAUUUU
GCUGUUAUGAAUGAAAUGCUACAUAUUUUU

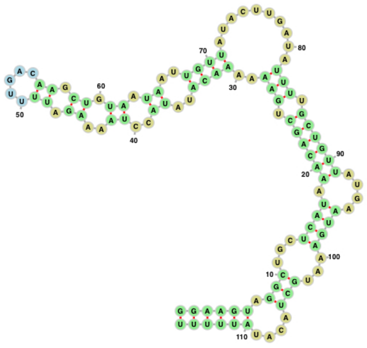

Predicted pseudo miRNAs in intron 1 of sheep *MSTN* gene

Start Position : 54
End Position : 163
Sequence Size : 110 nucleotides
Minimum Free Energy : -30.5 kcal/mol
Hairpin
(((((. ((. (. . . . . ((((((. (. ((((. (((. (((((. . (. ((((.
(..........).)))).)..))))).))).))))...).))))))...).)).)))))
Sequence
UUCAUGAGAAACCGAUCUAUUUUCAGGCUCUUUUAACAAGCUGCUGGCUUGUACGUAAGGAGGAGGGCAAAGAGCUUUUUGCAAG
ACUUCAUGAGAAAUAUGCUAAUGAG

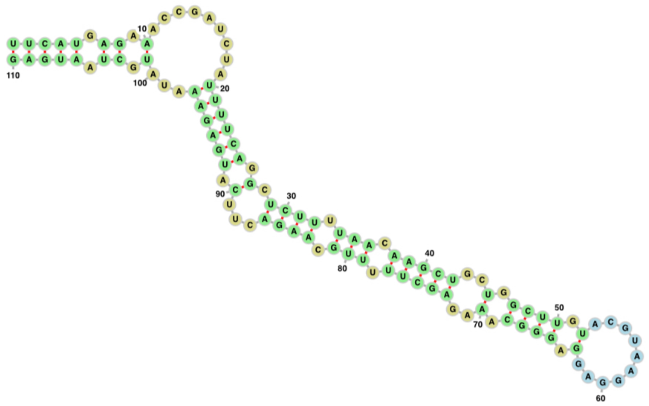

Start Position : 153
End Position : 240
Sequence Size : 88 nucleotides
Minimum Free Energy : -20.6 kcal/mol
Hairpin
((((((. . . . . (. ((((. . . . . (((. . ((((. ((. . . . .
((((....))))...)).)))).)))....)))).).))))))
Sequence
AUGCUAAUGAGACUGAAAGCUGCUACAUUAUCUGUUUCCUUAGAGAGCUAAAAAGCUAAAAAUCAGAAAUGAAAUGCUUGCAUAG
CAU

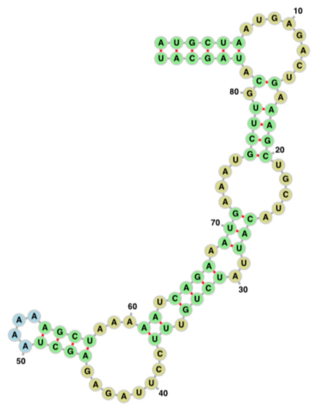

Start Position : 228
End Position : 292
Sequence Size : 65 nucleotides
Minimum Free Energy : -18.5 kcal/mol
Hairpin
(((.((((((....(((((((((..(((......)))...))))))))).))))))......)))
Sequence
GCUUGCAUAGCAUUCGUGUUAUAUAGUUUAGGAUGACAACUAUAACAUGUUUAUGUUUUCACAGC

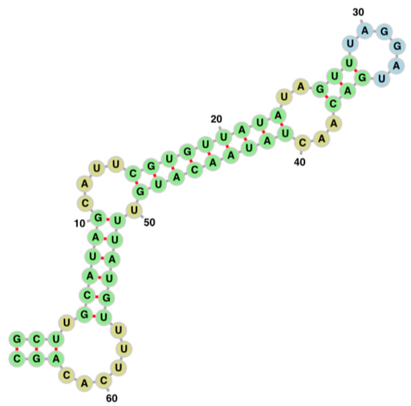

Start Position : 255
End Position : 362
Sequence Size : 108 nucleotides
Minimum Free Energy : -30.8 kcal/mol
Hairpin
((((((. . . . . ((. . (((((. . (. . ((((((((. . . . . . ((((((. . . .
((.............)).))))))....))))))))..).)))))..)).))))))
Sequence
UUAGGAUGACAACUAUAACAUGUUUAUGUUUUCACAGCUUAAUGCUACCAAGGUGAAGGAUUGGGAGACAGUAGCAGCCAUGUGA
AAAAUUUACAUGAAAUUUCCUAA

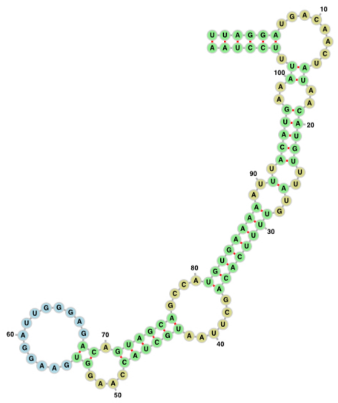

Start Position : 280
End Position : 389
Sequence Size : 110 nucleotides
Minimum Free Energy : -30.5 kcal/mol
Hairpin
(((. (((((. . . . . ((. ((((((. . . . . . ((((((((((. . . . .
(((((((.....)))))))...))))))))))...)))))).)).)))))....)))
Sequence
AUGUUUUCACAGCUUAAUGCUACCAAGGUGAAGGAUUGGGAGACAGUAGCAGCCAUGUGAAAAAUUUACAUGAAAUUUCCUAAUU
GCAUUUGGUUGCCUGAAAUAUGCAU

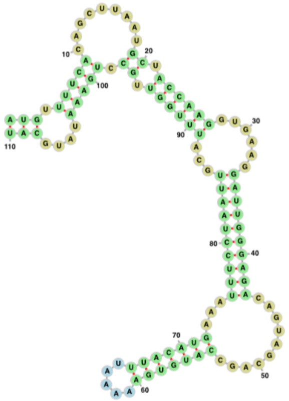

Start Position : 375
End Position : 462
Sequence Size : 88 nucleotides
Minimum Free Energy : -23.9 kcal/mol
Hairpin
((((..((((.(((((((........(((((((((((.
((........))...))))))))))).)))))))........)))).))))
Sequence
GCCUGAAAUAUGCAUUUAUAAUAACAGGGUUUUUUUCACUAAUAAAAGAGAAAGGAAGAAAUCUCUAGAUGUUGAAGCCUAUUUG
GGC

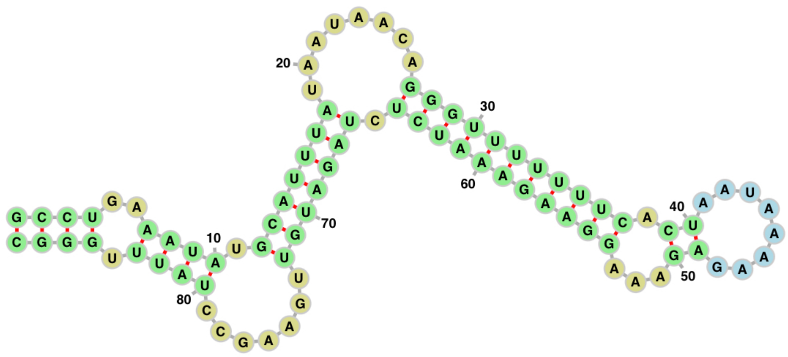

Start Position : 470
End Position : 582
Sequence Size : 113 nucleotides
Minimum Free Energy : -25.7 kcal/mol
Hairpin
(((((.(((...(..((.((((..(((((((.((.((((...((((.
(.....).))))...))))....)).))))))).......)))).))..)...))).....)))))
Sequence
GAACACUUAGAAUGACUUCUGUUAUUCAAAACUAUUUCUCAUAGGGUUUUUAUGUUCUUCAUAGAGUAUCUGAUUUUGAAAGCUA
UUACAGUGGAAAGGAUAAAAAAAUGUUC

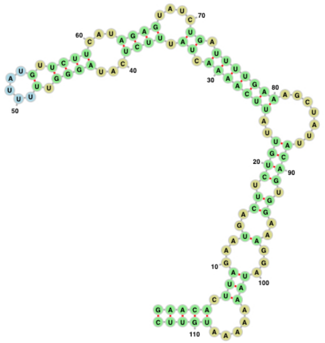

Start Position : 627
End Position : 725
Sequence Size : 99 nucleotides
Minimum Free Energy : -22 kcal/mol
Hairpin
(((.((((.(((...(..(((((((...(((......((((..
(((((....)))))).....)))).))).)))))))..).)))....))))...)))
Sequence
CAGCAUAAUGAUAAAUCAUGAGCCAAUGAGCAGAAAAUGCUAAGAAAUAAACAUUUUAAUUGAGUAGGUUAUGGCUUACAAAGUC
CCACUUAUACCCUG

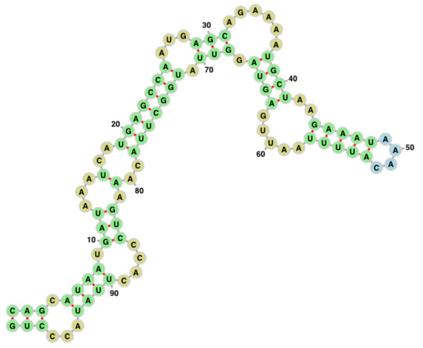

Start Position : 685
End Position : 785
Sequence Size : 101 nucleotides
Minimum Free Energy : -29.7 kcal/mol
Hairpin
((((((((.(.....(((........(((...(((...(.(((((.((((((.
(.....).))))))))))).)...)))...))).))).).))))))))
Sequence
AUUGAGUAGGUUAUGGCUUACAAAGUCCCACUUAUACCCUGACCAUGGUACUAUUGUUGAGAGUACCUGGUCUGCACAUAUCUAG
GAGGCACAUGCUUAAU

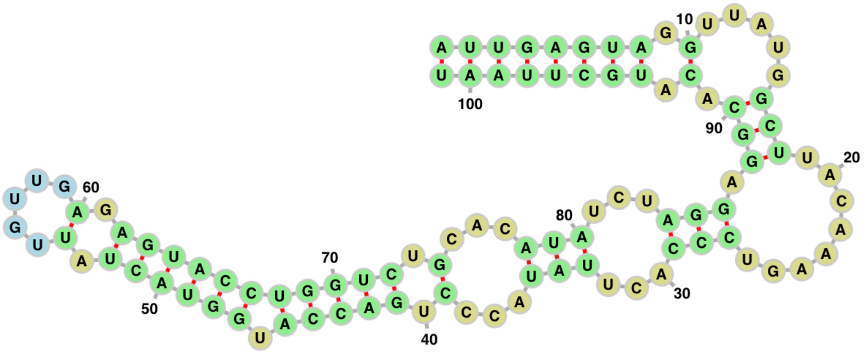

Start Position : 775
End Position : 841
Sequence Size : 67 nucleotides
Minimum Free Energy : -15.5 kcal/mol
Hairpin
((((((.(((((.((((((...........(((....)))..)))))).....)))))...))))))
Sequence
ACAUGCUUAAUAACCUUCUAAAAUAUUAUUUGAUUCCUCAUAGGAGGGAGAACUAUUACCUGUAUGU

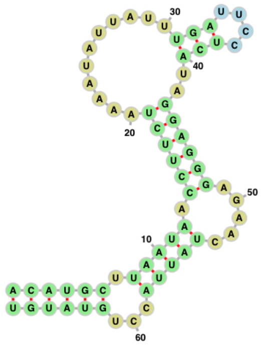

Start Position : 833
End Position : 929
Sequence Size : 97 nucleotides
Minimum Free Energy : -23.2 kcal/mol
Hairpin
(((((........(((......(((((....((..((((((((.((..(((.
(....).)))..))..))))))))..))..))))).))).)))))
Sequence
CCUGUAUGUAGUAUCUAUAUUGUUUCUGAAAGAUAAUAUGUUUCAUAUAUUUCUGUUGCAGUCAGUUCAAACAUAUACUCAAGGA
AAGGGAGACAGG

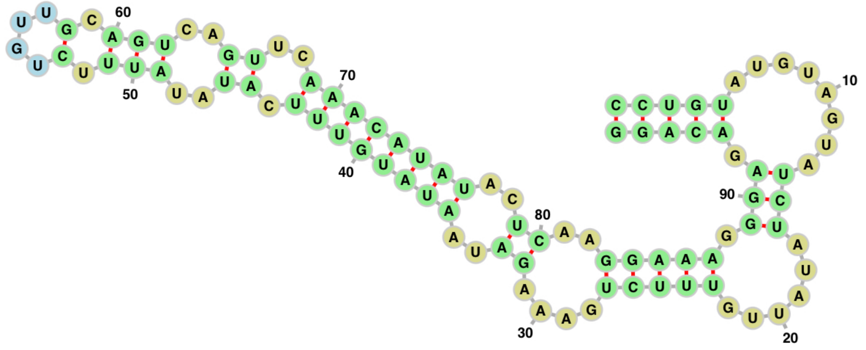

Start Position : 889
End Position : 992
Sequence Size : 104 nucleotides
Minimum Free Energy : -31 kcal/mol
Hairpin
((.(((((((((..((((((.(.((((((...((...((.((..
(((....)))...)).)).)).......)))))).)..))))))....))))))))).))
Sequence
UGCAGUCAGUUCAAACAUAUACUCAAGGAAAGGGAGACAGGCACCUCAACAGAGAAAGCAUGACCAGGAAGAUUUUUGUGCCGUG
UGUCUGCGAACUGGCUUUA

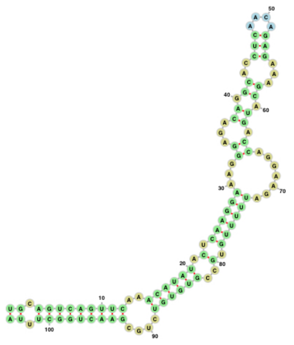

Start Position : 948
End Position : 1062
Sequence Size : 115 nucleotides
Minimum Free Energy : -27.1 kcal/mol
Hairpin
((((((.((...((((.((.((((..((((...((((((.((.........
(((.........)))....))..)))))))...)))).)))).....))..))))..)).))))))
Sequence
AUGACCAGGAAGAUUUUUGUGCCGUGUGUCUGCGAACUGGCUUUAUGCUACCCACUUUAGACUGGACUCAGAACAGUUUCAAAAU
ACUGGUUUUCUUAUUAAGUAACUAGGUUAU

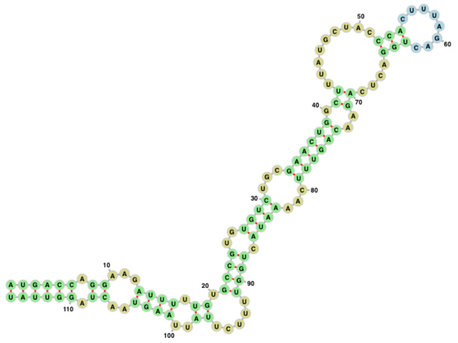

Start Position : 963
End Position : 1077
Sequence Size : 115 nucleotides
Minimum Free Energy : -24.8 kcal/mol
Hairpin
(((((...((.(((((...((((.((((.(((..(((...((.((((((..
(...)..)))))).))......)))).....))).)))).....))))...)))))))..)))))
Sequence
UUUGUGCCGUGUGUCUGCGAACUGGCUUUAUGCUACCCACUUUAGACUGGACUCAGAACAGUUUCAAAAUACUGGUUUUCUUAUU
AAGUAACUAGGUUAUAAGGCAACAAACAAA

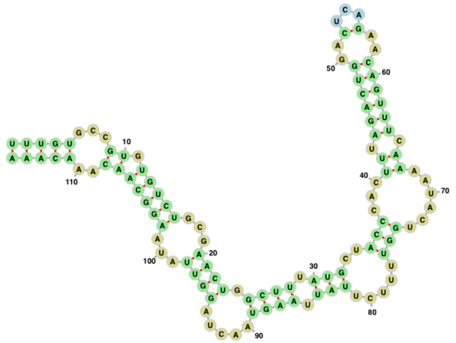

Start Position : 1023
End Position : 1114
Sequence Size : 92 nucleotides
Minimum Free Energy : -17.6 kcal/mol
Hairpin
((((((..((.(((((..(((((..........(((..((..
(........)..))..))).))))).......)))))...))..))))))
Sequence
GUUUCAAAAUACUGGUUUUCUUAUUAAGUAACUAGGUUAUAAGGCAACAAACAAAUUUCCUUUAAGACUGUGCUAUCAGAUAAUC
CUGGAAU

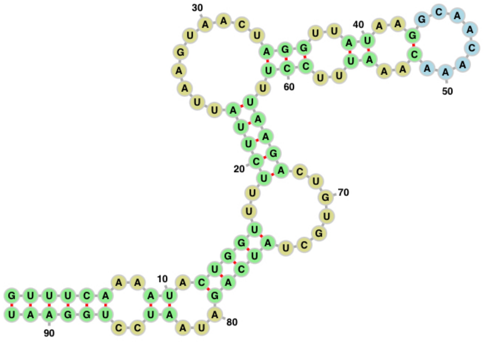

Start Position : 1098
End Position : 1197
Sequence Size : 100 nucleotides
Minimum Free Energy : -22.9 kcal/mol
Hairpin
((((((..((.((.(.((((.......((((((..((...
(((((..............)))))..))..))))))..)))).)..))..))..))))))
Sequence
UCAGAUAAUCCUGGAAUAGAUUUGCCUUAUUUAUAAACAAUCUUGAGAAAACAAAAAGGCAAGAAAUUGCUAAGUGCUUCUGCUU
ACAAUGACAGUCUGA

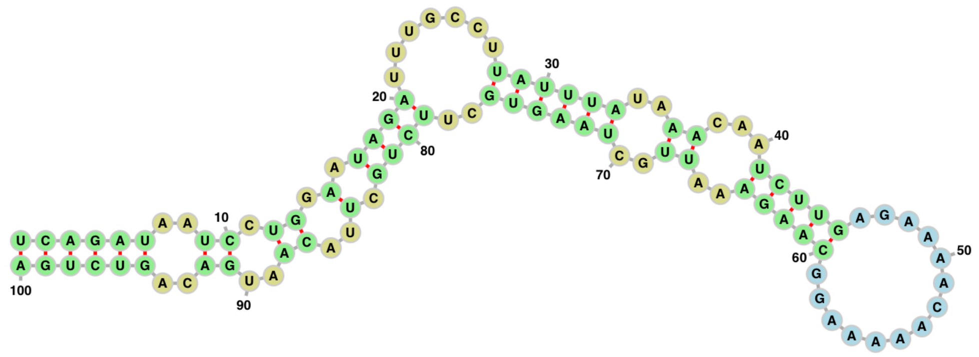

Start Position : 1125
End Position : 1239
Sequence Size : 115 nucleotides
Minimum Free Energy : -25.1 kcal/mol
Hairpin
((((((...(.((((...((((((((...(((..(((.((((..
((((......)))).)))).....)))...))).......)))))))).)))).).........))))))
Sequence
UAUUUAUAAACAAUCUUGAGAAAACAAAAAGGCAAGAAAUUGCUAAGUGCUUCUGCUUACAAUGACAGUCUGACCCUAAAGACAG
UGUUUUCUAGGUUUUGAAACAGCUUGAAUA

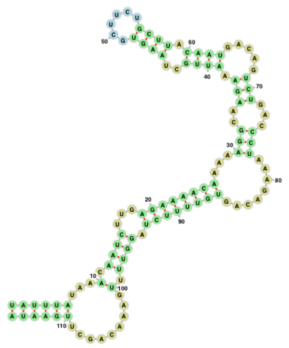

Start Position : 1205
End Position : 1305
Sequence Size : 101 nucleotides
Minimum Free Energy : -25.9 kcal/mol
Hairpin
((((((.((((..(((.(((((((.(((.(((.(((.(.
(.......).).)))....)))...)))...))))))).......)))..)))).))))))
Sequence
GACAGUGUUUUCUAGGUUUUGAAACAGCUUGAAUACAACAUCGAAAUUUUGGUGCCAAUUACCUGCUAGUUUUUAAAUUUUUUUU
CCUUUAAAAGGCUGUC

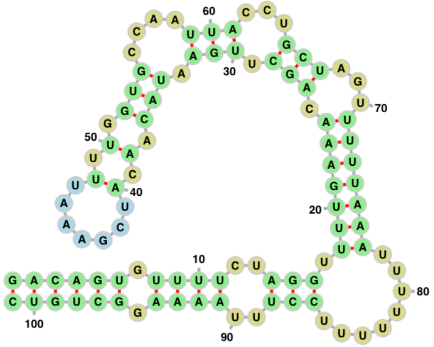

Start Position : 1235
End Position : 1338
Sequence Size : 104 nucleotides
Minimum Free Energy : -21.8 kcal/mol
Hairpin
(((((.(.((((.(..((..(((((.........(.((((((((.
((...........)).)))))))).)...)))))..))..)....))))...).)))))
Sequence
GAAUACAACAUCGAAAUUUUGGUGCCAAUUACCUGCUAGUUUUUAAAUUUUUUUUCCUUUAAAAGGCUGUCCCAGCGCCCUAACA
UAACAGAUGCACUAUAUUU

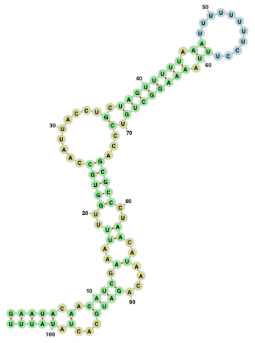

Start Position : 1297
End Position : 1380
Sequence Size : 84 nucleotides
Minimum Free Energy : -16.1 kcal/mol
Hairpin
(((((.((...((((..(((((.........((..(..(((.......)))....)..))...))))).)))).))...)))))
Sequence
AAGGCUGUCCCAGCGCCCUAACAUAACAGAUGCACUAUAUUUUCUACUAAUUCCCGAGGCUCAGUUAGUUGCUCACUGUGUCUU

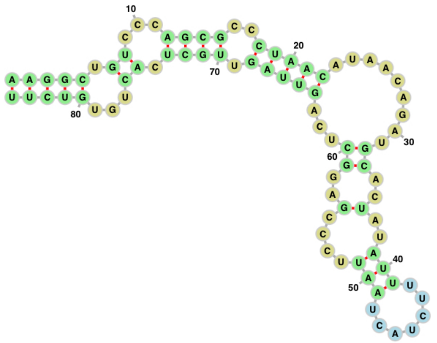

Start Position : 1423
End Position : 1514
Sequence Size : 92 nucleotides
Minimum Free Energy : -23.1 kcal/mol
Hairpin
(((..(((.((....((((....(.(((...(.(((((.(((.(.
(....).).))).))))).)...))).).)))))....)).))).)))
Sequence
AGACUGAUUGGUACAGCUGCUCAGUAAGUGUAACUACUCAGAUUCCCAAAGAAUUCUAAGUGGAUGUUCUUCCACAGUGUCUCUU
GUUCUCU

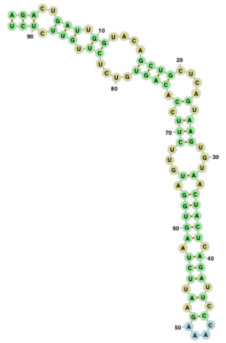

Start Position : 1462
End Position : 1567
Sequence Size : 106 nucleotides
Minimum Free Energy : -27.6 kcal/mol
Hairpin
((((((..((.((((...(((((((((...((..(.((((....(.
(((......))).)....)))).)..))...)))))))))...)))).))....))))))
Sequence
AGAUUCCCAAAGAAUUCUAAGUGGAUGUUCUUCCACAGUGUCUCUUGUUCUCUCUAAUCAUCAUCAUUUUAAAAUUUCAUCCACU
UUUCAUUCCUUAAUAGAAUUU

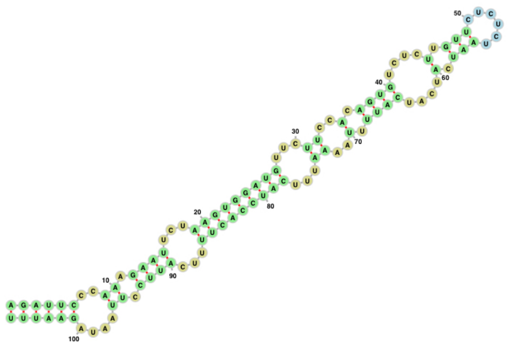

Start Position : 1523
End Position : 1618
Sequence Size : 96 nucleotides
Minimum Free Energy : -17.5 kcal/mol
Hairpin
(((...((((..(...((....(((....((((((.(.((((((.
(.........).)))))).)...))))))..))).)).)..))))...)))
Sequence
CAUCAUUUUAAAAUUUCAUCCACUUUUCAUUCCUUAAUAGAAUUUUCCUUAGUCCACAGUUCUCUGGAAAGGAAGUAGGCUGCUC
AUAAACAGCUG

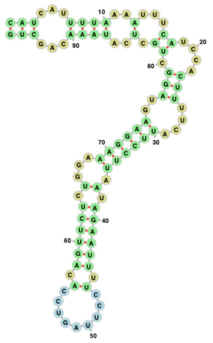

Start Position : 1684
End Position : 1786
Sequence Size : 103 nucleotides
Minimum Free Energy : -23.1 kcal/mol
Hairpin
(((((((((............((((((.((((....(((.........(((((.
((........)).)))))...))).)))).)))))))..)))))))))
Sequence
AUGAAUGAAAUGCUACAUAUUUUUCCAUUUUAAAAGACUAAAUAUGCACACAUUAUUCCAAUUUAAAAAAUGUUCAUAGAUUGAC
AUGGAGGCGUUCGUUCAU

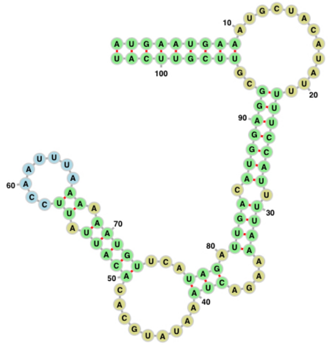

Supplement: Supplementary file 5 — Supplementary Material 5 [file 41598_2026_44326_MOESM5_ESM.pdf]
